# Supplementary material for: 1,2,4,5-Tetrazine-tethered probes for fluorogenically imaging superoxide in live cells with ultrahigh specificity
Source: Nat Commun. 2023 Mar 14;14:1401. doi: 10.1038/s41467-023-37121-8 (PMC10014963; doi:10.1038/s41467-023-37121-8)
Supplement: Supplementary file 1 — Supplementary information [file 41467_2023_37121_MOESM1_ESM.pdf]

Supplementary Table 1. Yields of O1 and the residue of Tz1 when Tz1 reacted with various analytes.

Group	Analytes	Tz1 Residue (%)	Yield of O1 (%)
**0**	Blank	100	0
**1**	KO_2	0	100
**2**	H_2O_2	98.77	1.23
**3**	$^3O_2$	98.48	1.52
**4**	ClO^-	87.52	1.18
**5**	*t*BHP	98.89	1.11
**6**	$ONOO^-$	70.06	7.80
**7**	NO	90.72	2.99
**8**	GSH	94.48	2.95

Supplementary Table 2. Stereoelectronic effects on the reactivity of tetrazine towards superoxide. All reactions were carried out in MeCN. Compounds were used at 100 μM final concentration and 0.5 eq $O_2^{\bullet-}$ was administrated. Measurement was carried out after 30 min of incubation. Conversion rate was calculated as the reduction of the peak area of the starting material.

Compound	Reduction Potentials	Conversion Rate (%)
**Tz1**	-0.887	10.6
**Tz2**	-0.946	6.03
**Tz3**	-0.744	33.73
**Tz4**	-	22.34

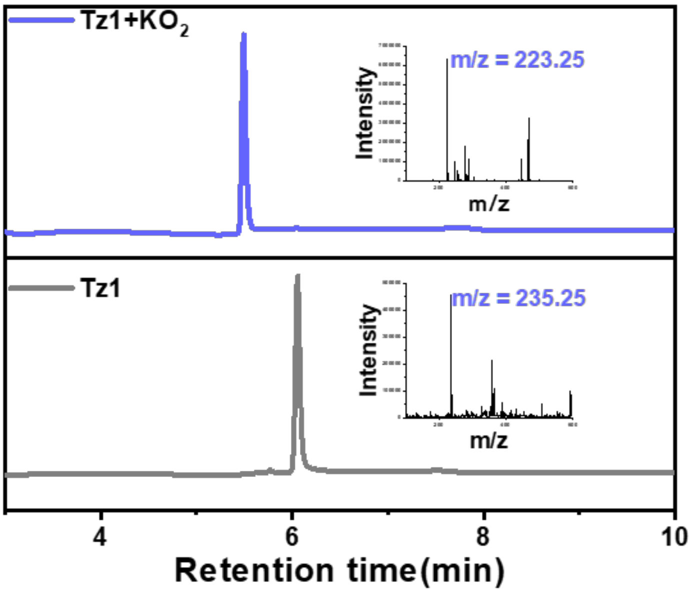

Supplementary Figure 1. LCMS traces of Tz1 (100 μM) before/after the treatment of KO_2. The reactions were carried out in MeCN at room temperature for 30 min.

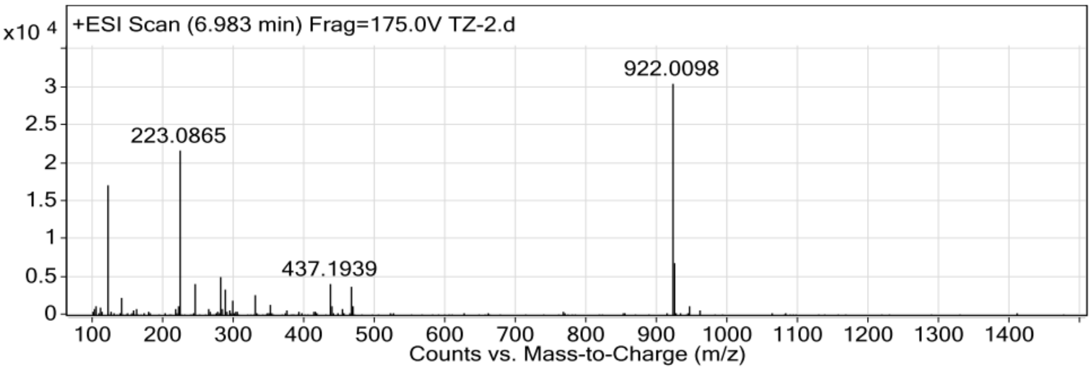

Supplementary Figure 2. The HRMS spectra of the product after Tz1 reacted with KO_2. ESI-HRMS (*m/z*): $[M+H]^+$ calc'd. for $C_{14}H_{11}N_2O$ 223.0871; found: 223.0865.

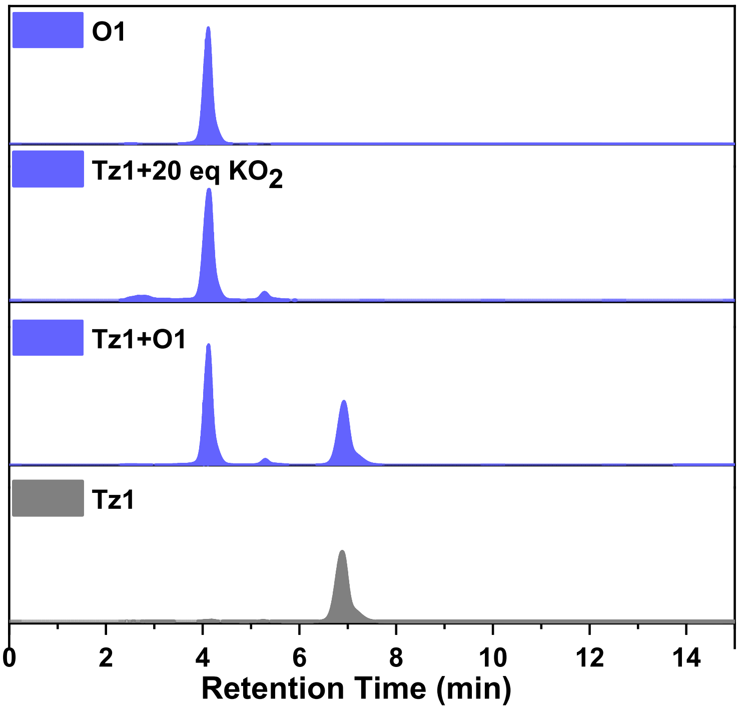

Supplementary Figure 3. HPLC traces of Tz1 before/after the treatment of KO_2 in comparison to O1. HPLC analysis was conducted with method A monitored at 280 nm.

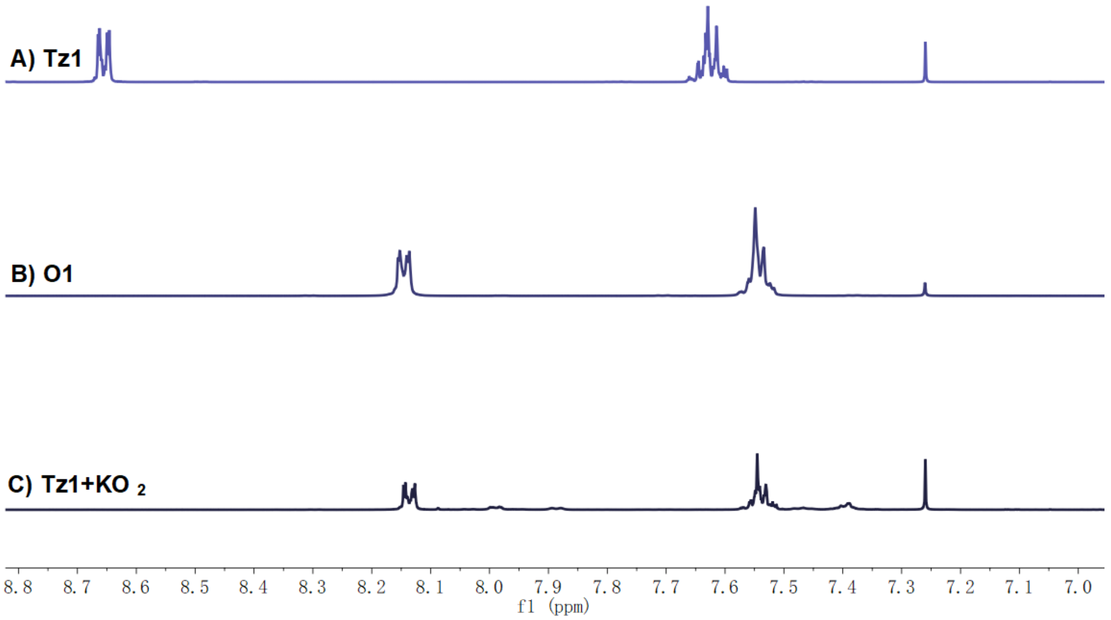

Supplementary Figure 4. NMR traces of Tz1 after the treatment of KO_2. A) ^{1}H-NMR traces of **Tz1**. B) ^{1}H-NMR traces of **O1**. C) ^{1}H-NMR traces of Tz1 treated with KO_2 without further purification. ^{1}H NMR (500 MHz, $CDCl_3$).

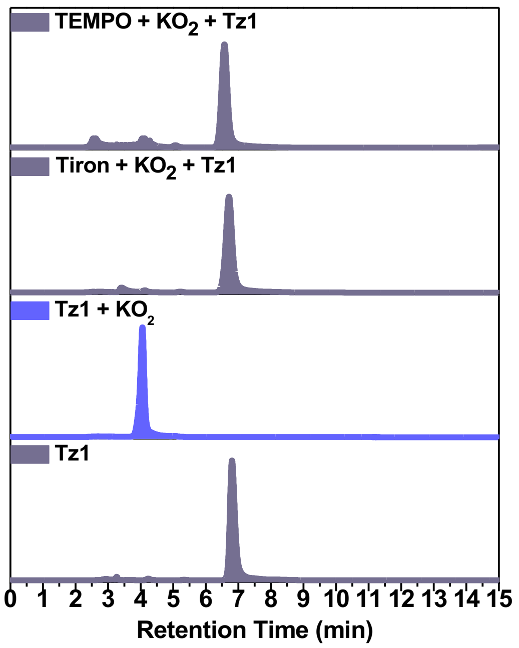

Supplementary Figure 5. HPLC traces of Tz1 after KO_2 treatment with/without the presence of Tiron or TEMPO. KO_2 was used at 20 eq, Tiron at 20 eq and TEMPO at 70 eq.

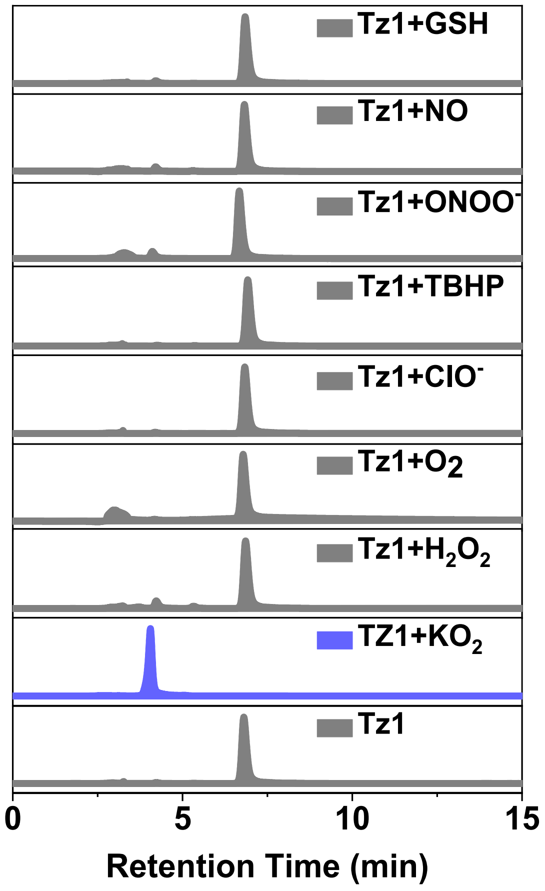

Supplementary Figure 6. HPLC traces of Tz1 after the treatment of various species. Tz1 was used at 100 µM. The reaction was carried out in a mixed solution of PBS (pH 7.4) and acetonitrile (50/50, v/v) for 30 min and then analyzed by HPLC method A monitored at 280 nm.

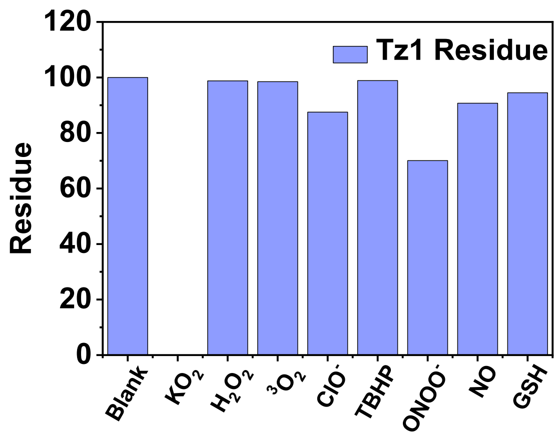

Supplementary Figure 7. The residual Tz1 after the treatment of various species as analyzed by HPLC. Tz1 was used at 100 µM. The reaction was carried out in a mixed solution of PBS (pH 7.4) and acetonitrile (50/50, v/v) for 30 min and then analyzed by HPLC method A monitored at 280 nm.

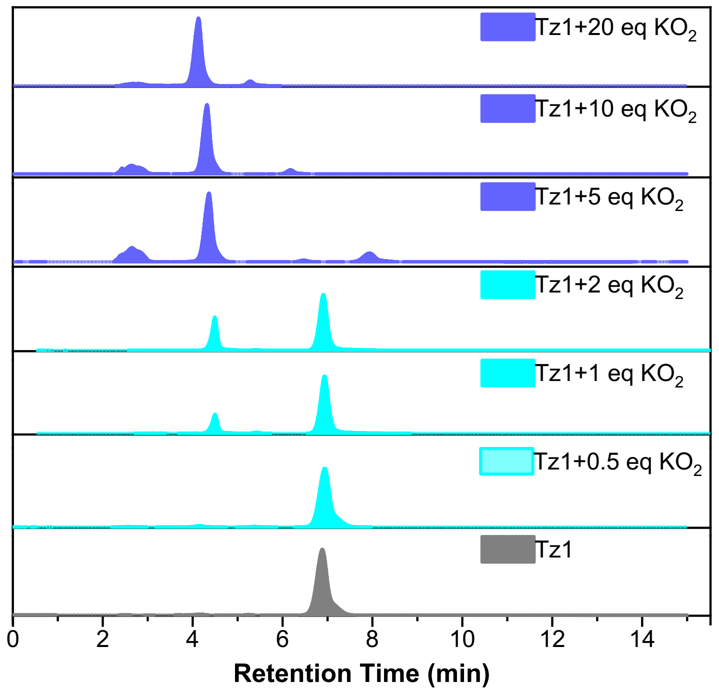

Supplementary Figure 8. HPLC traces of Tz1 after the treatment of various doses of KO_2. Tz1 was used at 100 µM. The reaction was carried out in a mixed solution of PBS (10 mM, pH 7.4) and acetonitrile (50/50, v/v) for 30 min then analyzed by HPLC method A monitored at 280 nm.

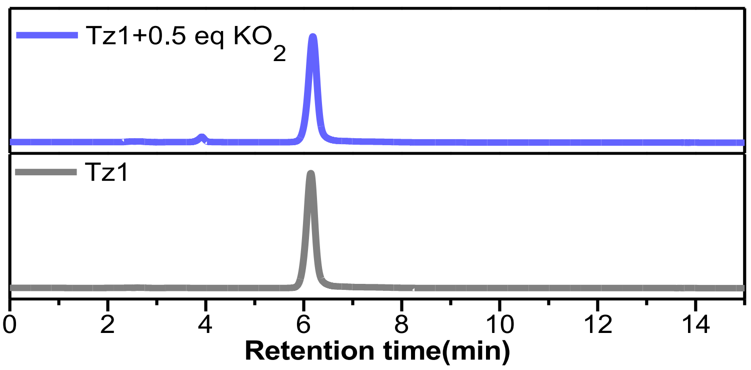

Supplementary Figure 9. HPLC traces of Tz1 before/after the treatment of KO_2. Tz1 was used at 100 µM, and was incubated with KO_2 (50 µM) in acetonitrile for 30 min. HPLC was performed with method A and monitored at 280 nm.

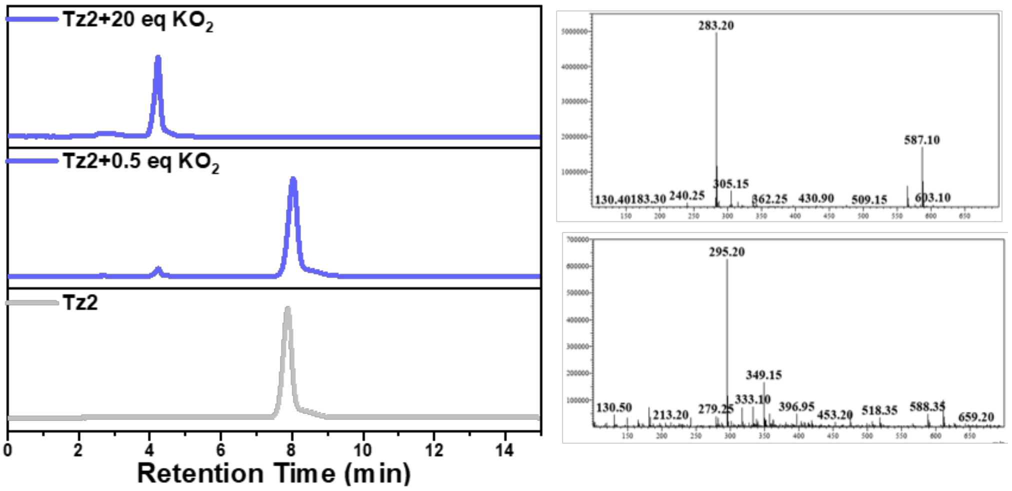

Supplementary Figure 10. HPLC traces and LCMS data of Tz2 before/after the treatment of KO_2. Tz2 was used at 100 µM, and was incubated with KO_2 (50 µM or 2 mM) in acetonitrile for 30 min. HPLC was performed with method A and monitored at 325 nm. The sample (**Tz2** group and **Tz**2 with 20 eq KO_2 group) was simultaneously diluted and analyzed by LCMS, with the mass spectra of the main peak shown in the right panel.

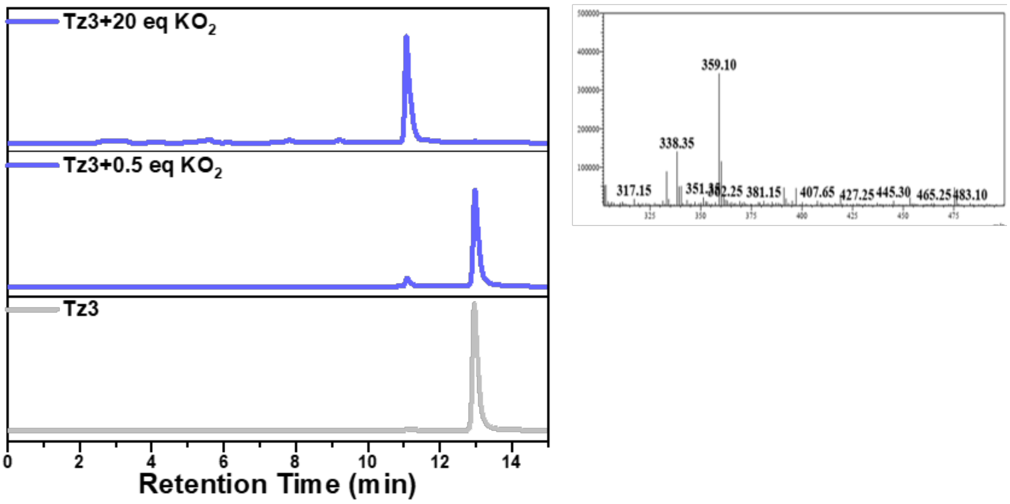

Supplementary Figure 11. HPLC traces and LCMS data of Tz3 before/after the treatment of KO_2. **Tz3** was used at 100 μM, and was incubated with KO_2 (50 μM or 2 mM) in acetonitrile for 30 min. HPLC was performed with method B and monitored at 280 nm. The samples (**Tz3** group and **Tz3** with 20 eq KO_2 group) were simultaneously diluted and analyzed by LCMS, with the mass spectra of the main peak shown in the right panel.

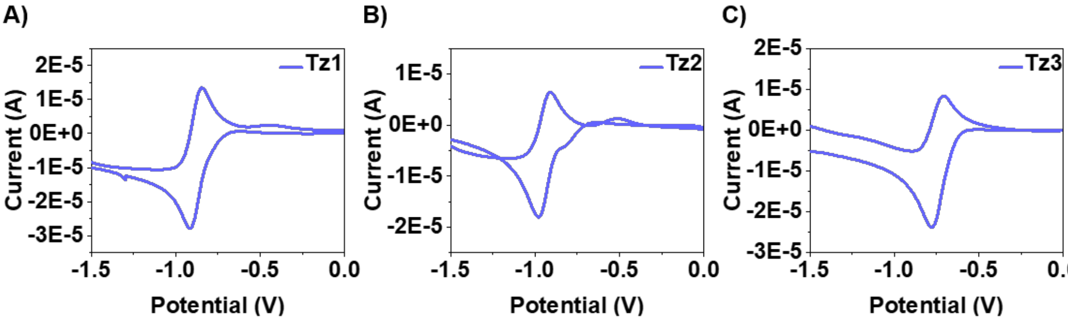

Supplementary Figure 12. Cyclic voltammetry (CV) curves of Tz1-Tz3. Tz1-Tz3 (1 mM) were measured in 0.1 M Et_4NClO_4 solution of CH_3CN.

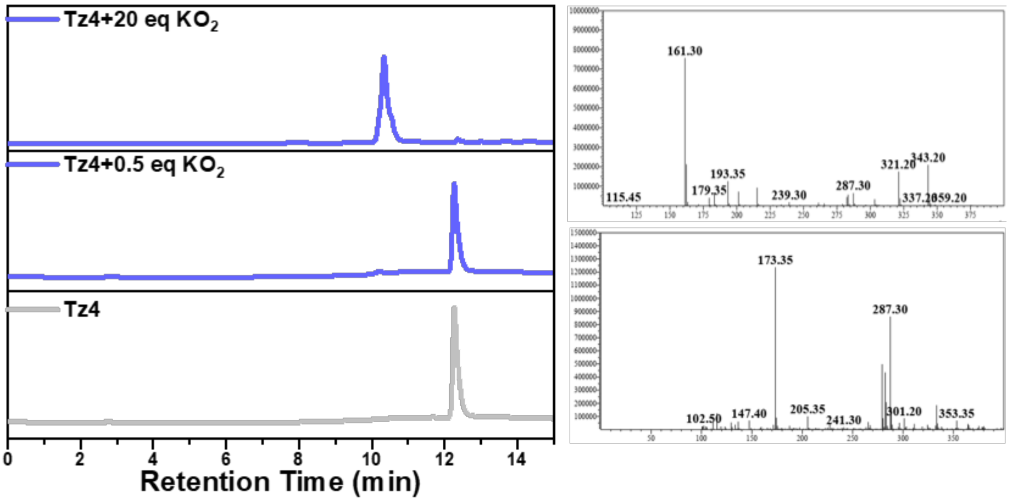

Supplementary Figure 13. HPLC traces and LCMS data of Tz4 before/after the treatment of KO_2. **Tz4** was used at 100 µM, and was incubated with KO_2 (50 µM or 2 mM) in acetonitrile for 30 min. HPLC was performed with method C and monitored at 254 nm. The sample (**Tz4** group and **Tz4** with 20 eq KO_2 group) was simultaneously diluted and analyzed by LCMS, with the mass spectra of the main peak shown in the right panel.

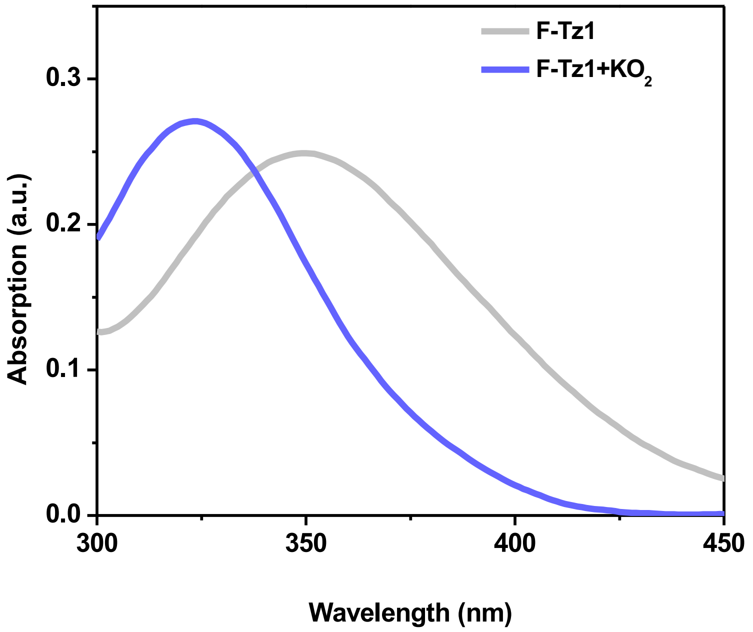

Supplementary Figure 14. UV-Vis absorption spectra of F-Tz1 before/after the treatment of KO_2. **F-Tz1** was used at 20 µM. 20 eq KO_2 was used. The incubation time was 30 min. (a u., absorption unit)

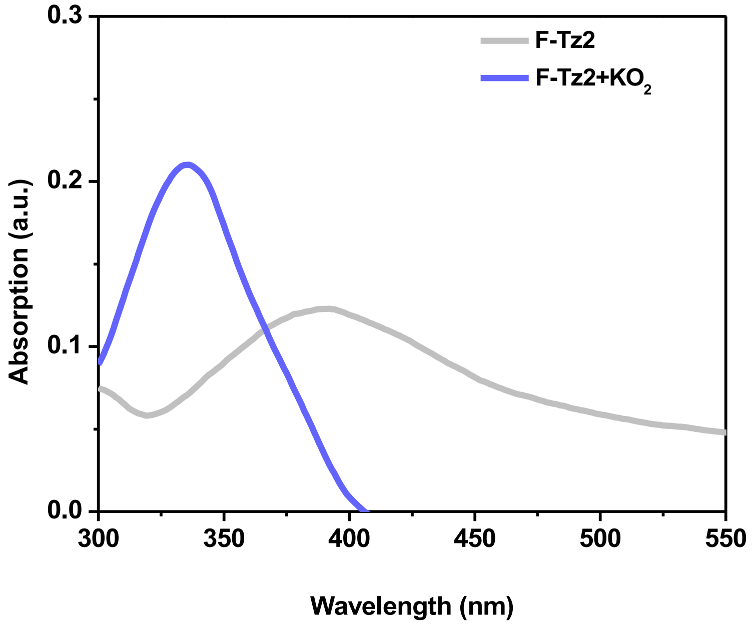

Supplementary Figure 15. UV-Vis absorption spectra of F-Tz2 before/after the treatment of KO_2. **F-Tz2** was used at 20 μM. 20 eq KO_2 was used. The incubation time was 30 min. (a u., absorption unit)

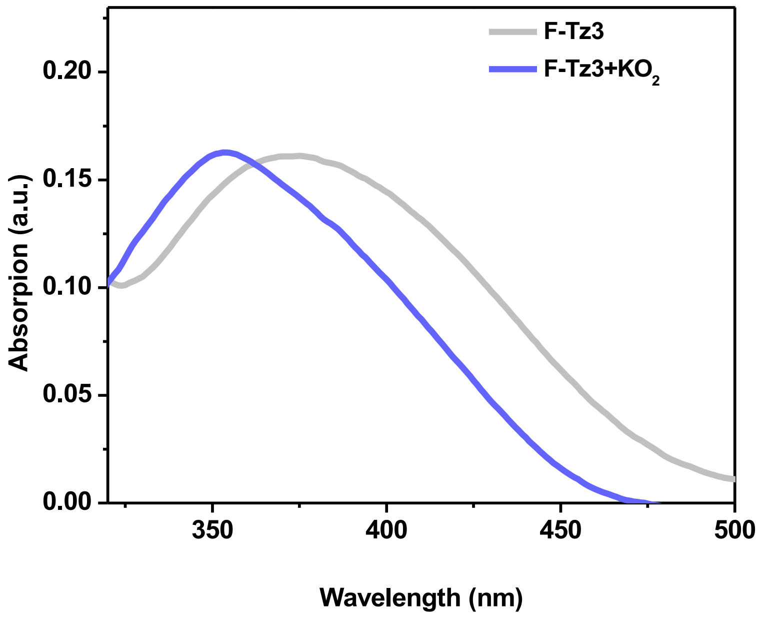

Supplementary Figure 16. UV-Vis absorption spectra of F-Tz3 before/after the treatment of KO_2. **F-Tz3** was used at 20 μM. 20 eq KO_2 was used. The incubation time was 30 min. (a u., absorption unit)

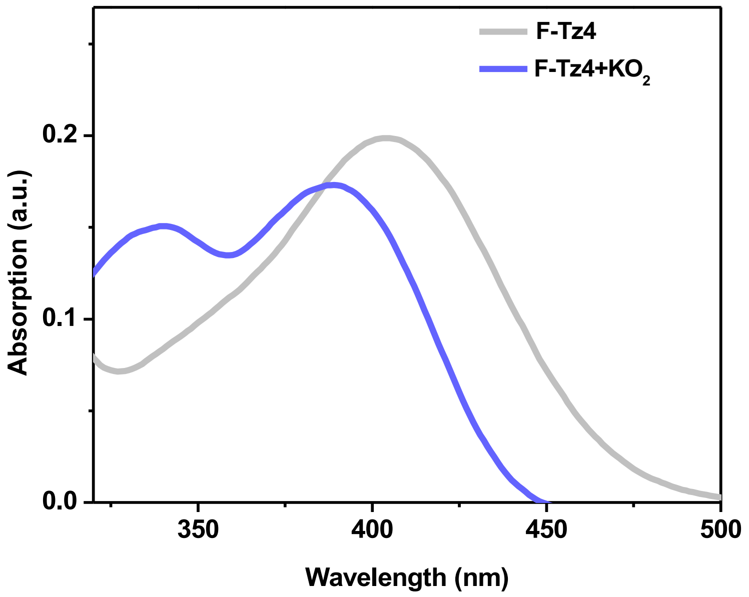

Supplementary Figure 17. UV-Vis absorption spectra of F-Tz4 before/after the treatment of KO_2. **F-Tz4** was used at 20 μM. 20 eq KO_2 was used. The incubation time was 30 min. (a u., absorption unit)

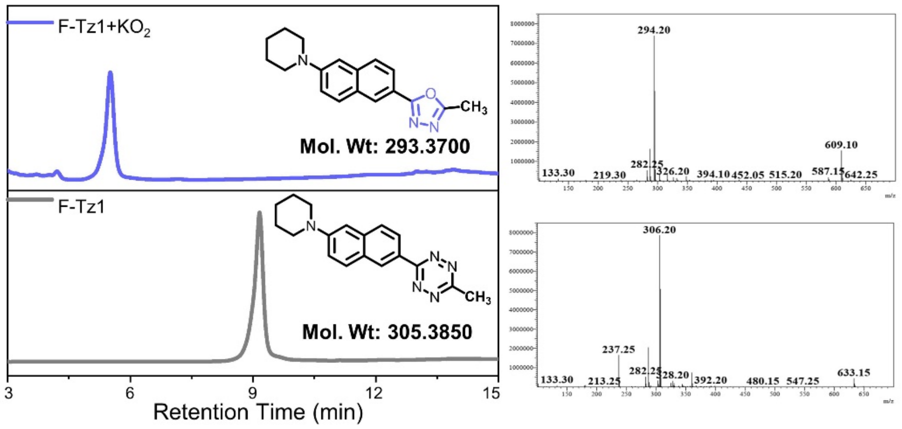

Supplementary Figure 18. HPLC and LCMS data of F-Tz1 before/after the treatment of KO_2. F-Tz1 was used at 100 μM, and treated with 20 eq KO_2 in acetonitrile for 30 min. HPLC was performed with method D monitored at 300 nm. The sample was simultaneously diluted and analyzed by LCMS, with the mass spectra of the main peak shown in the right panel.

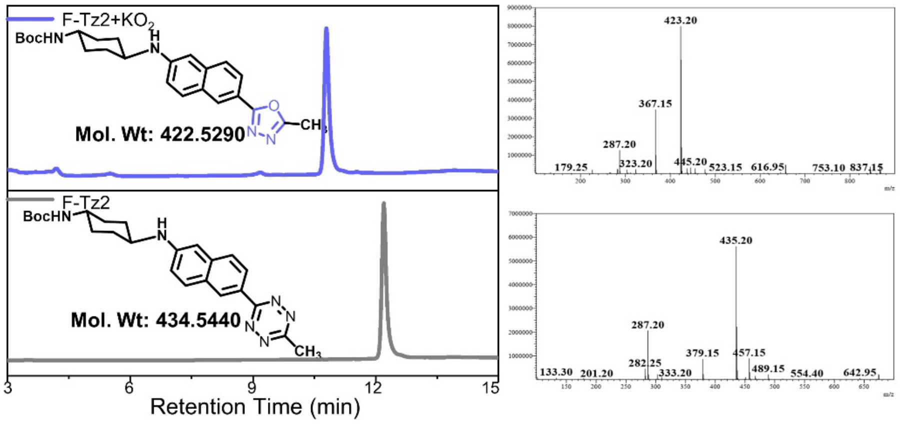

Supplementary Figure 19. HPLC and LCMS data of F-Tz2 before/after the treatment of KO_2. F-Tz2 was used at 100 μM, and treated with 20 eq KO_2 in acetonitrile for 30 min. HPLC was performed with method D monitored at 300 nm. The sample was simultaneously diluted and analyzed by LCMS, with the mass spectra of the main peak shown in the right panel.

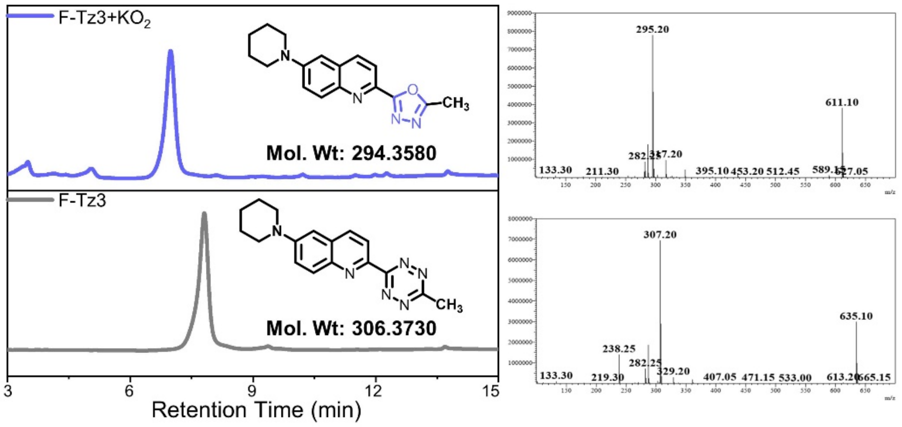

Supplementary Figure 20. HPLC and LCMS data of F-Tz3 before/after the treatment of KO_2. F-Tz3 was used at 100 μM, and treated with 20 eq KO_2 in acetonitrile for 30 min. HPLC was performed with method D monitored at 300 nm. The sample was simultaneously diluted and analyzed by LCMS, with the mass spectra of the main peak shown in the right panel.

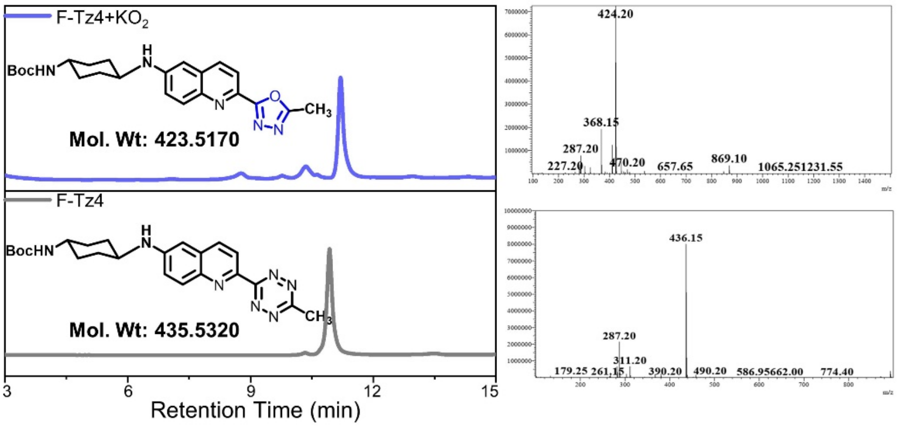

Supplementary Figure 21. HPLC and LCMS data of F-Tz4 before/after the treatment of KO_2. F-Tz4 was used at 100 μM, and treated with 20 eq KO_2 in acetonitrile for 30 min. HPLC was performed with method D monitored at 300 nm. The sample was simultaneously diluted and analyzed by LCMS, with the mass spectra of the main peak shown in the right panel.

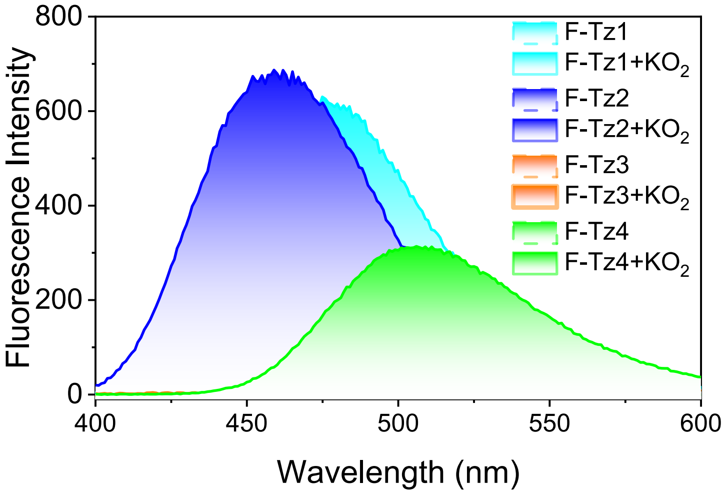

Supplementary Figure 22. Fluorescence spectra of F-Tz1-F-Tz4 before/after the treatment of $O_2^{\bullet-}$. The probes were used at 5 μM, and were treated with 100 μM $O_2^{\bullet-}$ for 30 min.

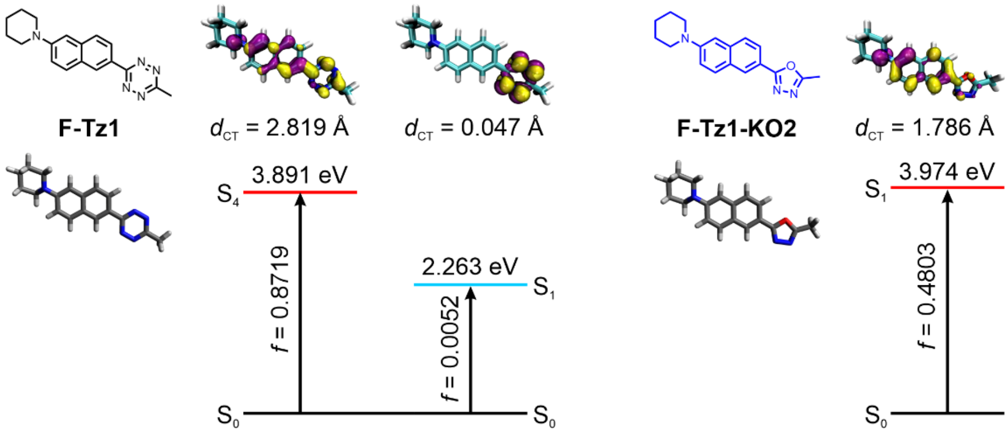

Supplementary Figure 23. Quantum chemical calculations of F-Tz1. Molecular structures, optimized geometries, and electron-hole distributions of the selected vertical excited states of **F-Tz1** before (the left panel) and after reactions (the right panel) with superoxide. The insets show the vertical excitation energy, corresponding oscillator strength (f), and charge transfer distance (d_{CT}) of these states.

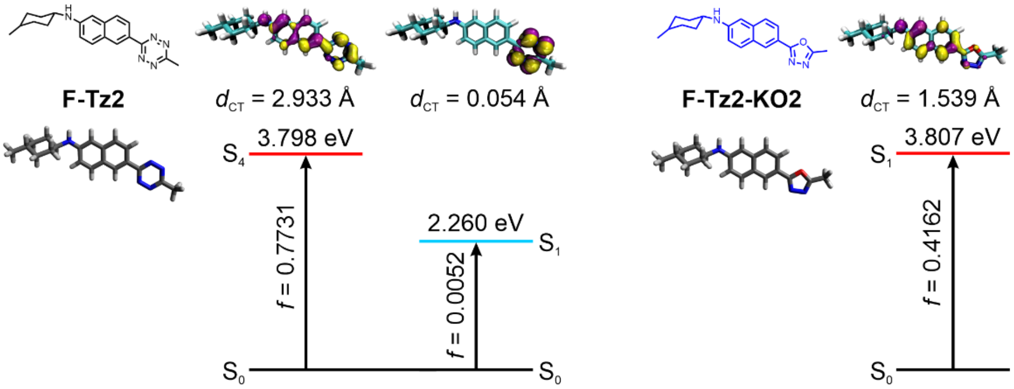

Supplementary Figure 24. Quantum chemical calculations of F-Tz2. Molecular structures, optimized geometries, and electron-hole distributions of the selected vertical excited states of **F-Tz2** before (the left panel) and after reactions (the right panel) with superoxide. The insets show the vertical excitation energy, corresponding oscillator strength (f), and charge transfer distance (d_{CT}) of these states.

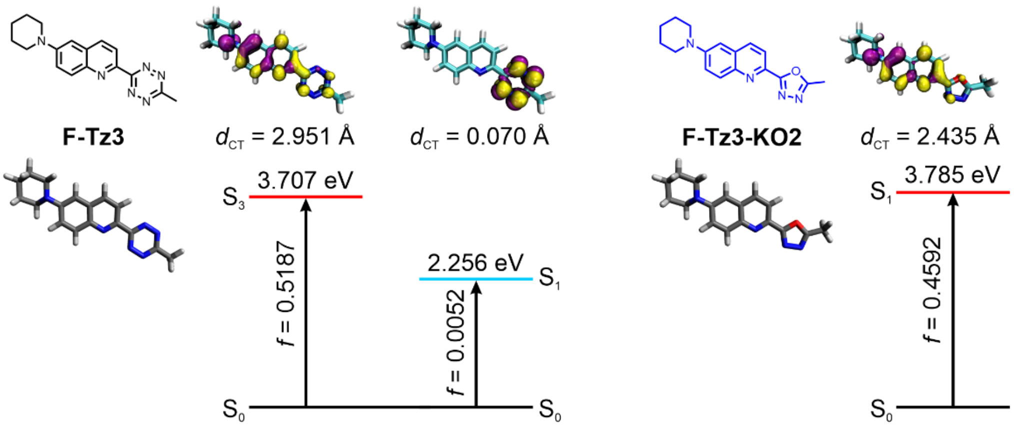

Supplementary Figure 25. Quantum chemical calculations of F-Tz3. Molecular structures, optimized geometries, and electron-hole distributions of the selected vertical excited states of **F-Tz3** before (the left panel) and after reactions (the right panel) with superoxide. The insets show the vertical excitation energy, corresponding oscillator strength (f), and charge transfer distance (d_{CT}) of these states.

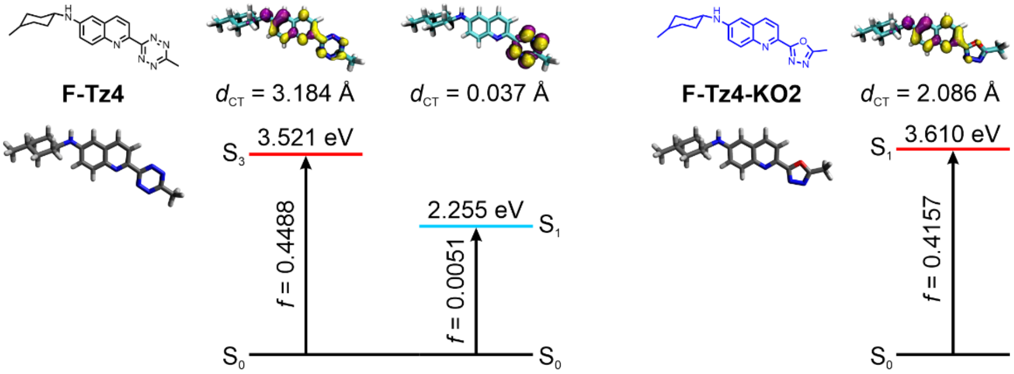

Supplementary Figure 26. Quantum chemical calculations of F-Tz4. Molecular structures, optimized geometries, and electron-hole distributions of the selected vertical excited states of **F-Tz4** before (the left panel) and after reactions (the right panel) with superoxide. The insets show the vertical excitation energy, corresponding oscillator strength (f), and charge transfer distance (d_{CT}) of these states.

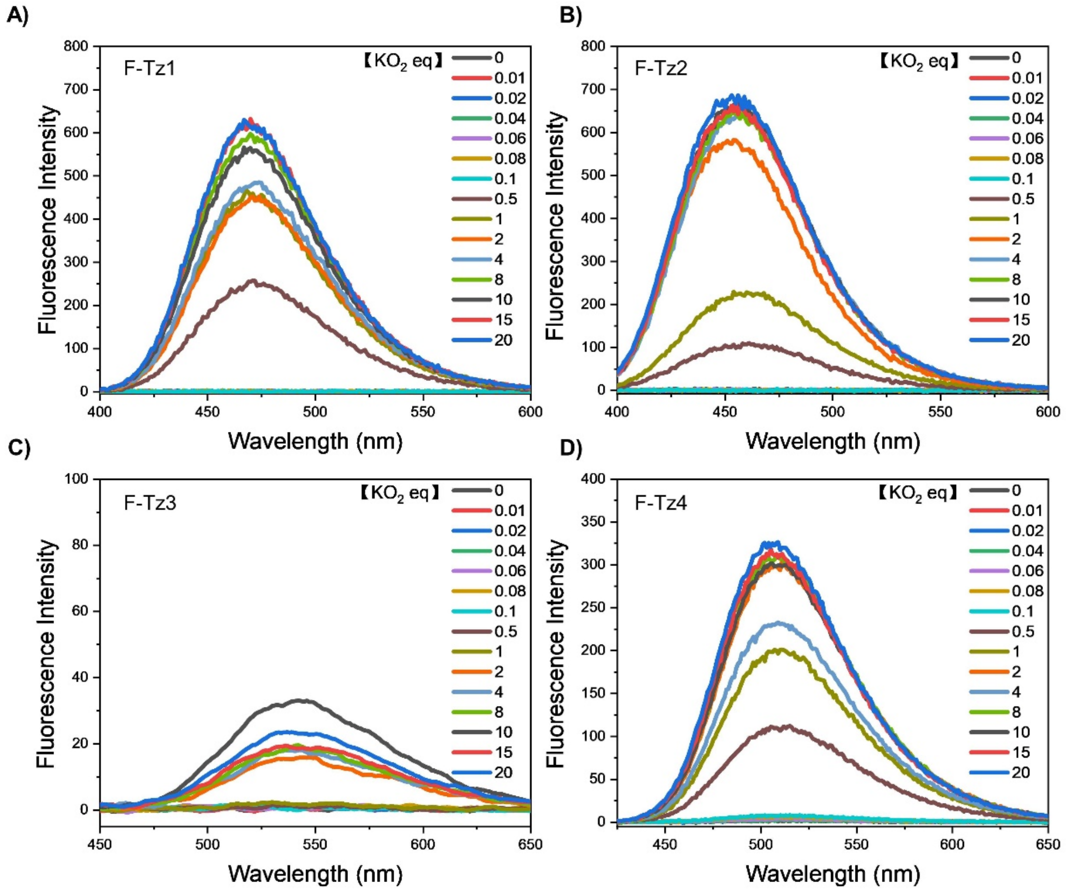

Supplementary Figure 27. Fluorescence spectra of F-Tz1-F-Tz4 after the treatment of $O_2^{\bullet-}$. The probes were used at 5 μM and were treated with 0-20 eq $O_2^{\bullet-}$ for 30 min. Then the spectra were recorded.

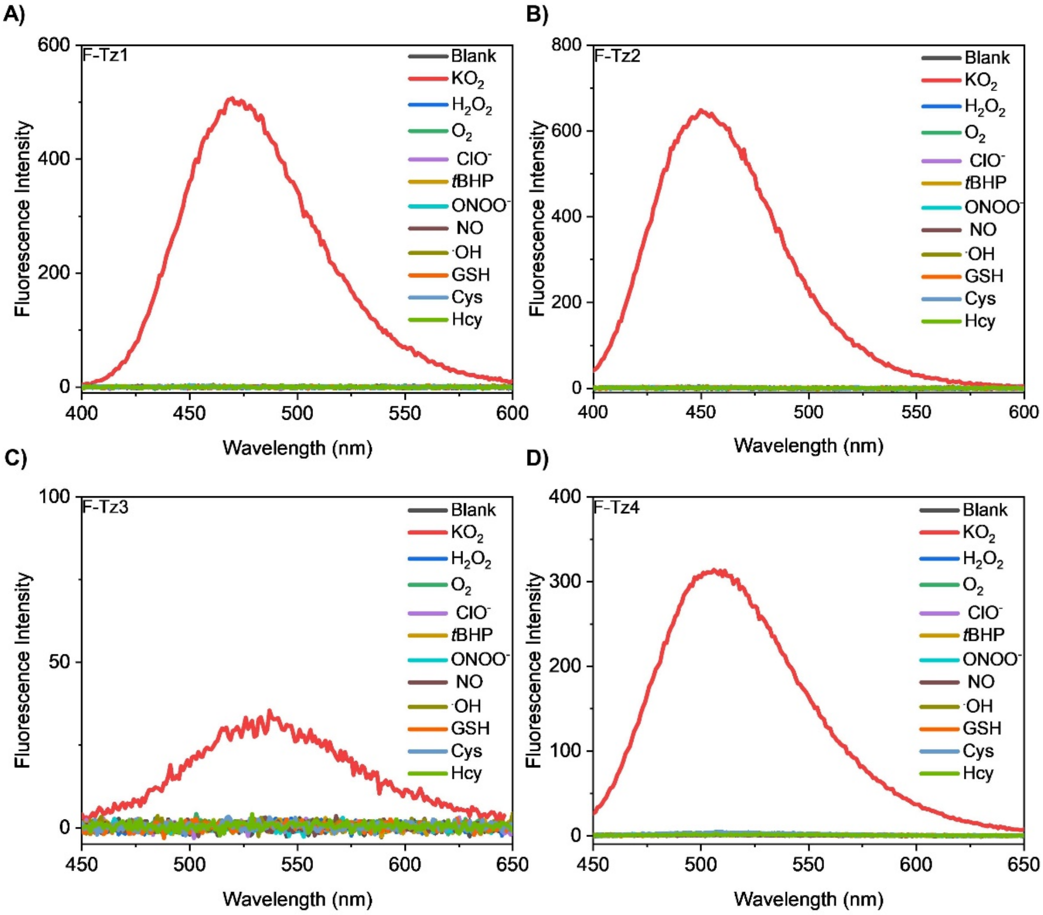

Supplementary Figure 28. Fluorescence spectra of F-Tz1-F-Tz4 after the treatment of various analytes. All probes were used at 5 μM and the reactive species were used at 100 μM except $ONOO^-$ (10 μM) and NO (20 μM). The reactions were carried out at ambient temperature for 30 min. Then the spectra were recorded for A) **F-Tz1**, B) **F-Tz2**, C) **F-Tz3**, D) **F-Tz4**.

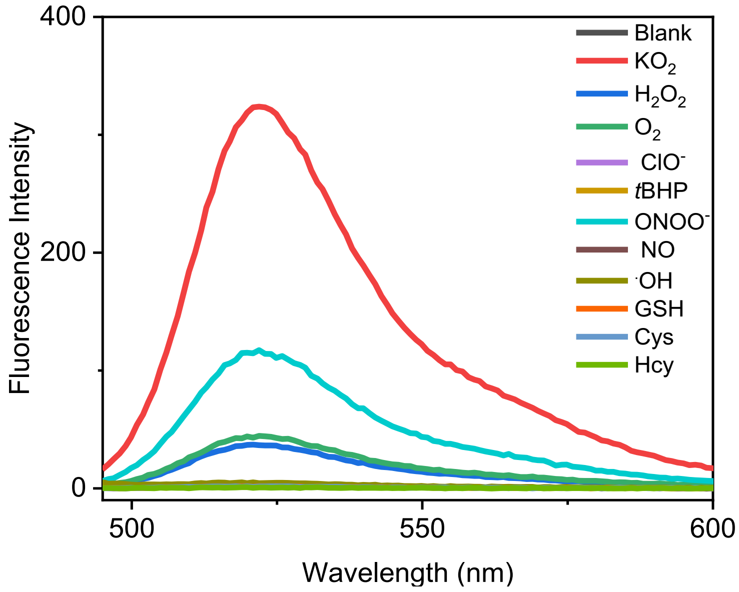

Supplementary Figure 29. Fluorescence spectra of DCFHDA after the treatment of various analytes. All probes were used at 5 μM and the reactive species were used at 100 μM except $ONOO^-$ (10 μM) and NO (20 μM). The reactions were carried out at ambient temperature for 30 min. Then the spectra were recorded.

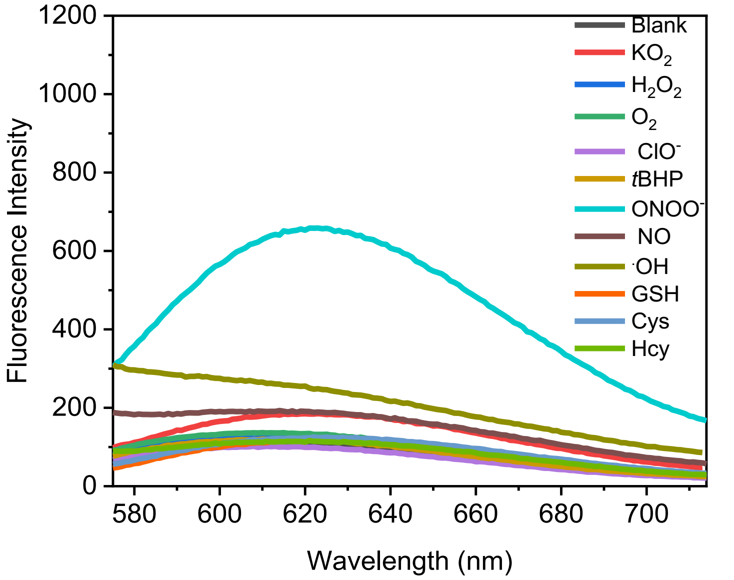

Supplementary Figure 30. Fluorescence spectra of DHE after the treatment of various analytes. All probes were used at 5 μM and the reactive species were used at 100 μM except $ONOO^-$ (10 μM) and NO (20 μM). The reactions were carried out at ambient temperature for 30 min. Then the spectra were recorded.

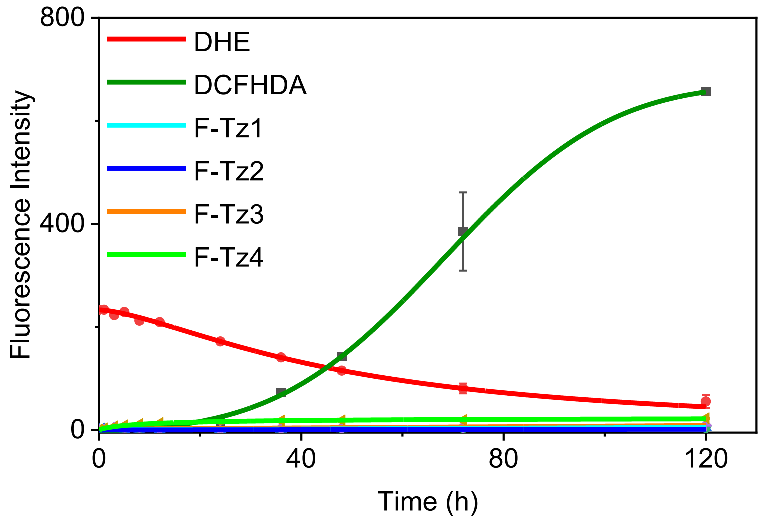

Supplementary Figure 31. Storage stability of F-Tz1-F-Tz4 in comparison to DCFHDA and DHE. Probes **F-Tz1**- **F-Tz4**, DCFHDA, and DHE were dissolved in PBS buffer (10 mM, pH 7.4) to make 5 μM solutions, and were kept at ambient temperature in the dark room. Their fluorescence spectra were recorded at 0, 1, 3, 5, 8, 12, 24, 36, 48, 72, and 120 h time points, and the intensities at their emission maxima were plotted against storage time. Data were presented as mean value ± SD, n = 3 independent experiments. (**F-Tz1**, λ_{ex} /λ_{em} = 323/470 nm; **F-Tz2**, λ_{ex} /λ_{em} = 384/460 nm; **F-Tz3**, λ_{ex} /λ_{em} = 350/530 nm; **F-Tz4**, λ_{ex} /λ_{em} = 385/510 nm; DCFHDA, λ_{ex} /λ_{em} = 488/525 nm; DHE, λ_{ex} /λ_{em} = 370/420 nm) (DHE is a ratiometric probe that emits blue light without ROS).

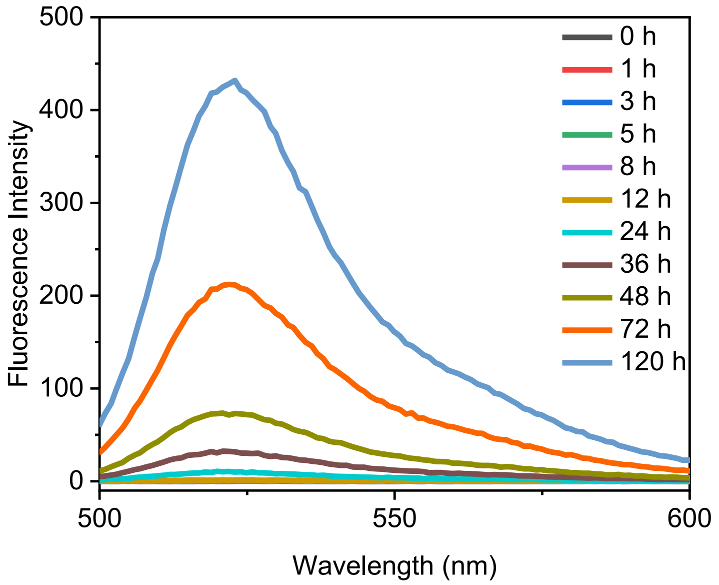

Supplementary Figure 32. Storage stability of DCFHDA. Data shown were the fluorescence spectra of DCFHDA in PBS buffer (10 mM, pH 7.4) after different storage time.

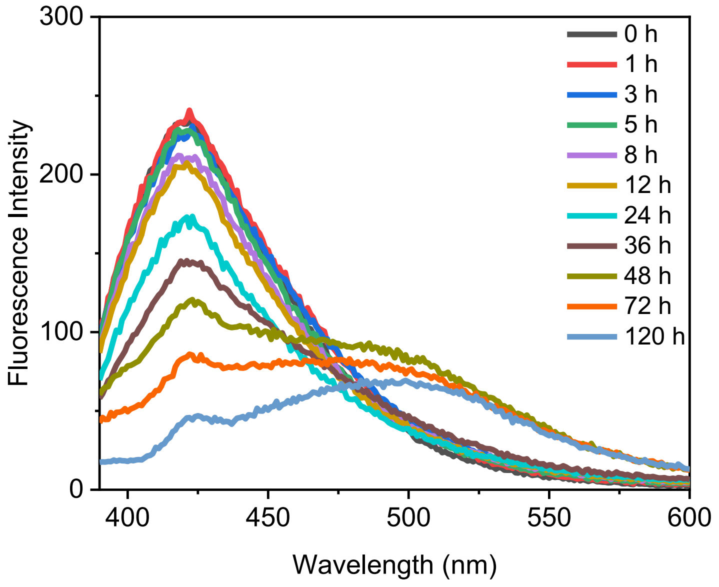

Supplementary Figure 33. Storage stability of DHE. Data shown were the fluorescence spectra of DHE in PBS buffer (10 mM, pH 7.4) after different storage time.

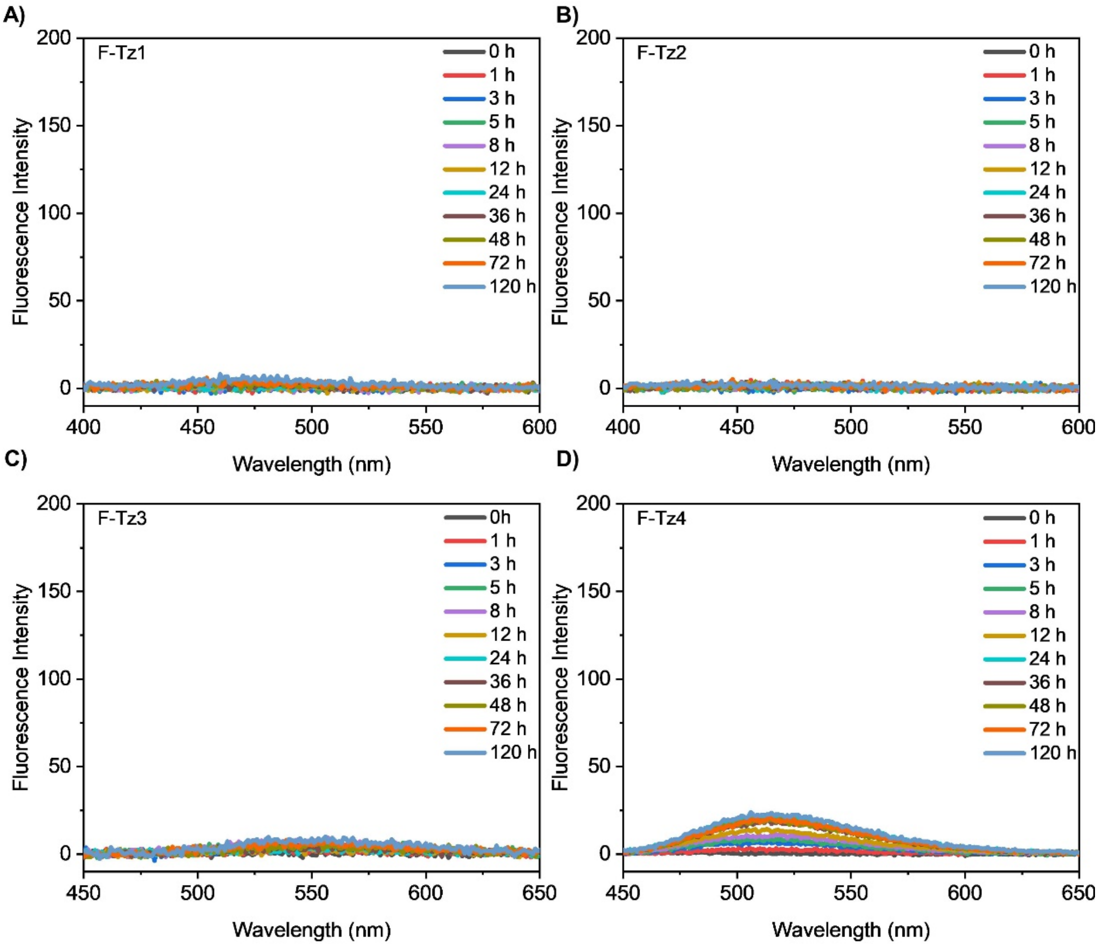

Supplementary Figure 34. Storage stability of F-Tz1-F-Tz4. Data shown were the fluorescence spectra of A) **F-Tz1**, B) **F-Tz2**, C) **F-Tz3**, D) **F-Tz4** in PBS buffer (10 mM, pH 7.4) after different storage time.

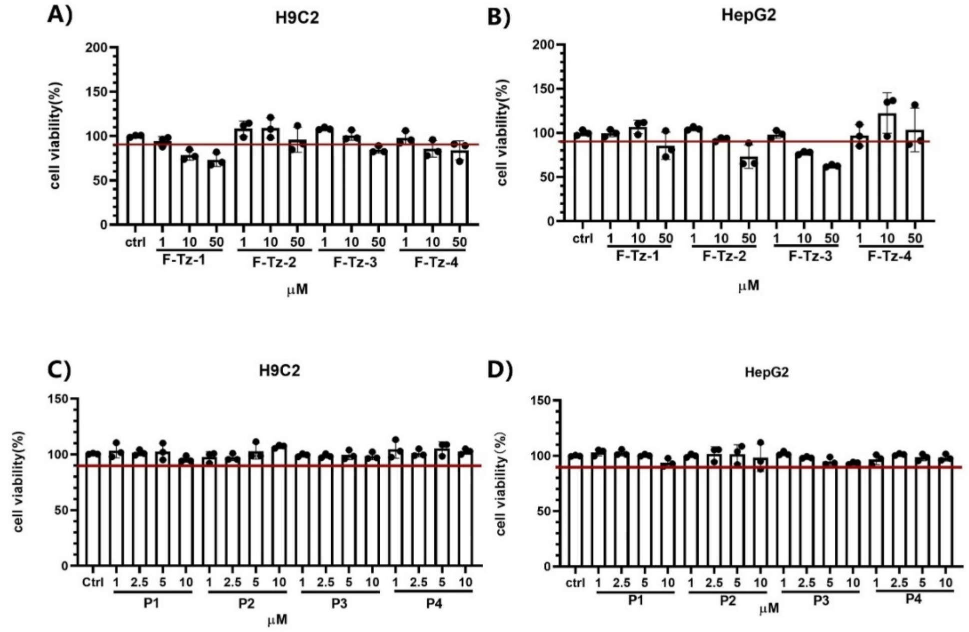

Supplementary Figure 35. Cytotoxicity of F-Tz1-F-Tz4 and their oxadiazole-derivatives by MTT assay. A, C) Cytotoxicity assay of probes (A) and oxadiazoles (C) against H9C2 cells. After co-incubating different concentrations of compounds with H9C2 cells for 24 h, the cell viability was evaluated by MTT assay. B, D) Cytotoxicity assay of probes (B) and oxaiazoles (D) against HepG2 cells. After co-incubating different concentrations of probes with HepG2 cells for 24 h, the cell viability was evaluated by MTT assay. Data shown were mean ± SD, n = 3 biologically independent experiments.

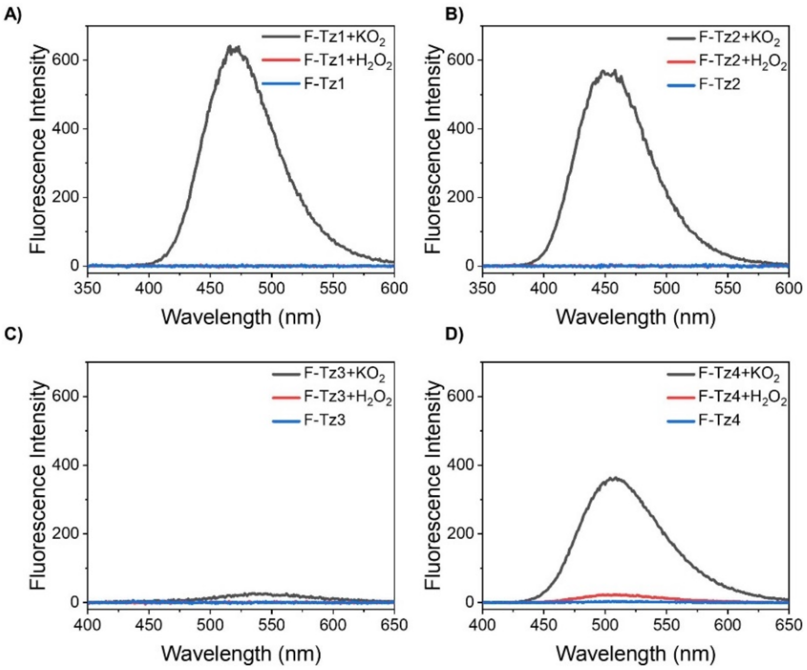

Supplementary Figure 36. Fluorescence spectra of F-Tz1-F-Tz4 after the treatment of H_2O_2/KO_2. All probes were used at 5 μM. H_2O_2 was used at 2 mM. KO_2 was used at 100 μM. The reactions were carried out at ambient temperature for 30 min.

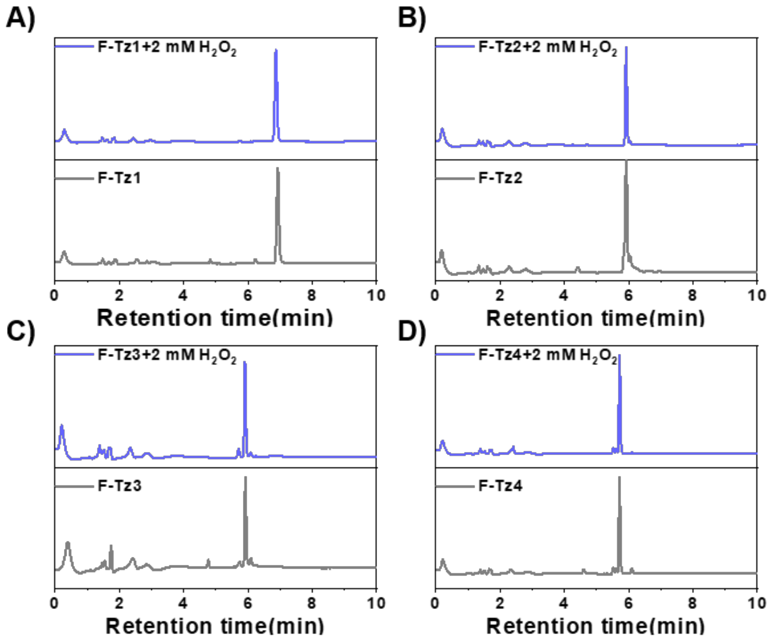

Supplementary Figure 37. LC traces of F-Tz1-F-Tz4 before/after the the treatment of H_2O_2. All probes were used at 100 μM. H_2O_2 was used at 2 mM and the reactions were carried out at ambient temperature for 30 min. Then the mixtures were analyzed by LCMS.

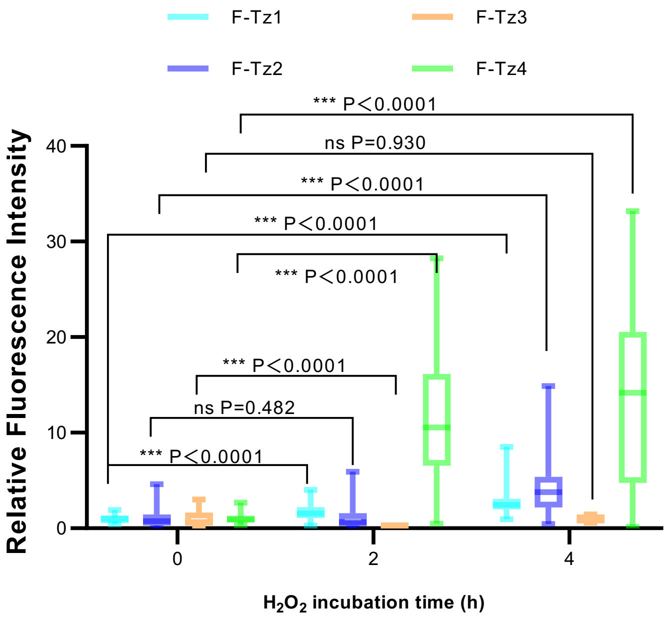

Supplementary Figure 38. Quantified relative mean fluorescence intensity of the cells in Figure 2F. HepG2 cells pretreated with H_2O_2 (2 mM) for various time were stained with either the naphthalene-tetrazines (**F-Tz1** or **F-Tz2**) or quinoline-tetrazines (**F-Tz3** or **F-Tz4**). Probes were used at a final concentration of 5 μM and were incubated with the cells for 30 min before imaging. Data were normalized to the negative control group. P values were analyzed by two-tailed unpaired t-test, 95% Confidence interval. **F-Tz1**: n = 34 cells for 2 mM H_2O_2 0 h, n = 58 cells for 2 mM H_2O_2 2 h, n = 62 cells for 2 mM H_2O_2 4 h; **F-Tz2**: n = 81 cells for 2 mM H_2O_2 0 h, n = 89 cells for 2 mM H_2O_2 2 h, n = 84 cells for 2 mM H_2O_2 4 h; **F-Tz3**: n = 76 cells for 2 mM H_2O_2 0 h, n = 57 cells for 2 mM H_2O_2 2 h, n = 73 cells for 2 mM H_2O_2 4 h; **F-Tz4**: n =88 cells for 2 mM H_2O_2 0 h, n =128 cells for 2 mM H_2O_2 2 h, n =58 cells for 2 mM H_2O_2 4 h. All cell numbers are over 3 indenpendent experiments. For the boxplots, the top and bottom lines of each box represent the 75th and 25th percentiles of the samples, respectively. The line inside each box represents the median of the samples. The upper and lower lines above and below the boxes are the whiskers. ns: not significant, ***P < 0.001 versus untreated cells.

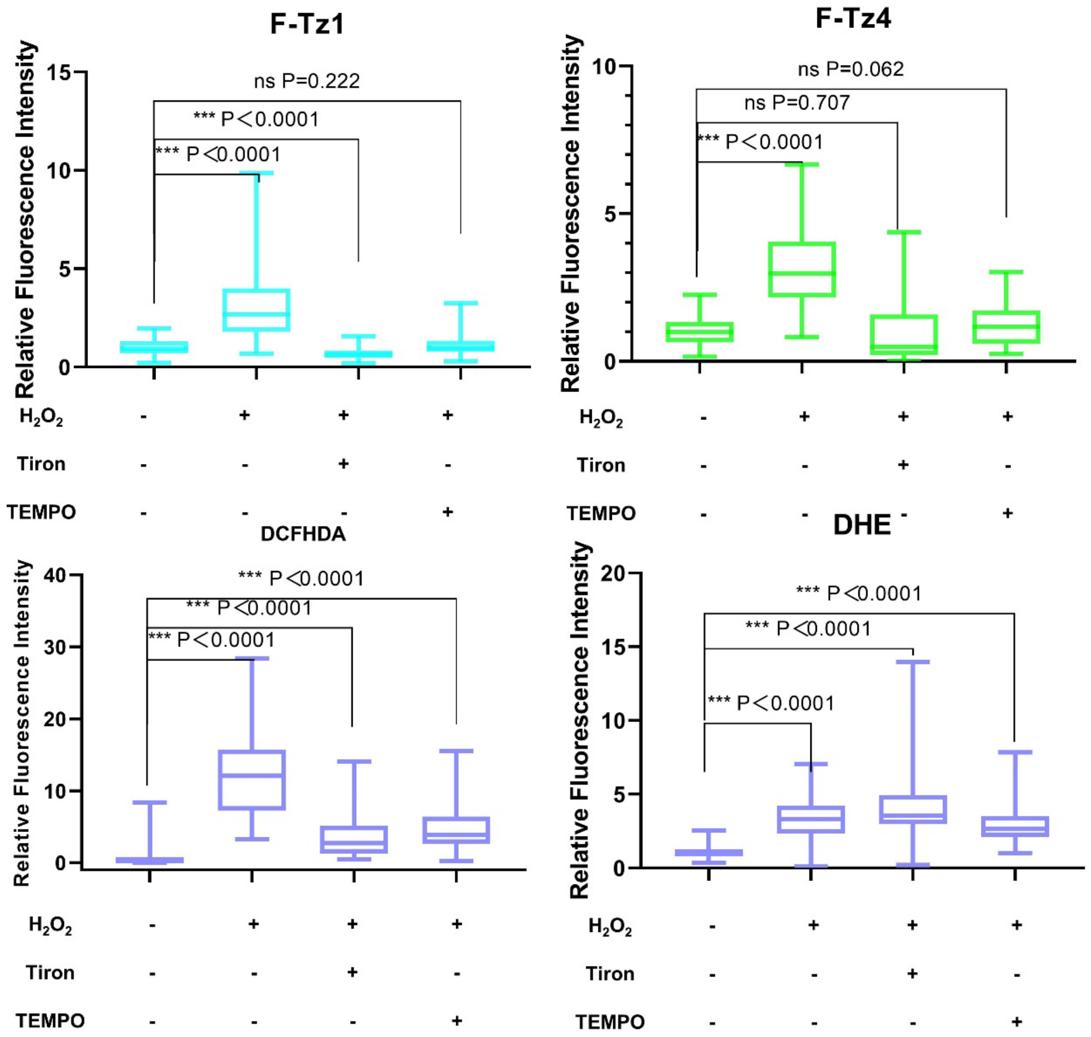

Supplementary Figure 39. Quantified relative mean fluorescence intensity of the cells in Figure 2G. Before being stained with the probes, cells were intact (control), or pretreated with H_2O_2 (2 mM) for 2 h, or first pretreated with Trion (100 μM) or TEMPO (300 μM) for 1 h and then the co-treatment of Tiron (100 μM) or TEMPO (300 μM) together with H_2O_2 (2 mM) for 2 h. Cells were then stained with **F-Tz1**, **F-Tz4**, DCFHDA or DHE (each 5 μM, 30 min), and then imaged. P values were analyzed by two-tailed unpaired t-test, 95% Confidence interval. **F-Tz1**: n =44 cells for control group, n =67 cells for H_2O_2 group, n =58 cells for Tiron group, n =44 cells for TEMPO group; **F-Tz4**: n =51 cells for control group, n =80 cells for H_2O_2 group, n =79 cells for Tiron group, n =93 cells for TEMPO group; DCFHDA: n =43 cells for control group, n =46 cells for H_2O_2 group, n =67 cells for Tiron group, n =53 cells for TEMPO group; DHE: n =62 cells for control group, n =113 cells for H_2O_2 group, n =58 cells for Tiron group, n =63 cells for TEMPO group. All cell numbers are over 3 indenpendent experiments. For the boxplots, the top and bottom lines of each box represent the 75th and 25th percentiles of the samples, respectively. The line inside each box represents the median of the samples. The upper and lower lines above and below the boxes are the whiskers. ns: not significant, ***$P < 0.001$ versus untreated cells.

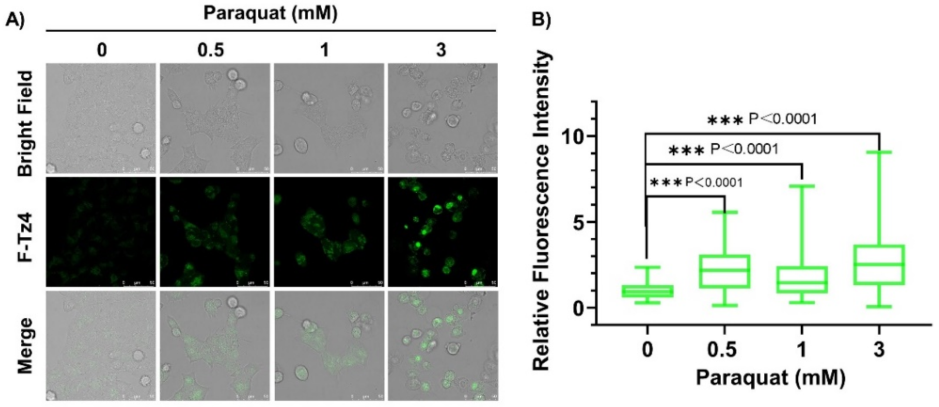

Supplementary Figure 40. F-Tz4 imaging of endogenous superoxide in live HepG2 cells under oxidative stress conditions stimulated by Paraquat. A) Confocal microscopy images of HepG2 cells treated with different concentrations (0 - 3 mM) of paraquat for 24 h, and then stained with **F-Tz4** (5 μM) for 30 min before imaging (Scale bar: 50 μm). B) Quantified relative mean fluorescence intensity of the cells. Data were normalized to the negative control group. P values were analyzed by two-tailed unpaired t-test, 95% Confidence interval. n =191 cells for 0 mM Paraquat, n =144 cells for 0.5 mM Paraquat, n =108 cells for 1 mM Paraquat, n =140 cells for 3 mM Paraquat. All cell numbers are over 3 indenpendent experiments. For the boxplots, the top and bottom lines of each box represent the 75th and 25th percentiles of the samples, respectively. The line inside each box represents the median of the samples. The upper and lower lines above and below the boxes are the whiskers. ns: not significant, ***P < 0.001 versus untreated cells.

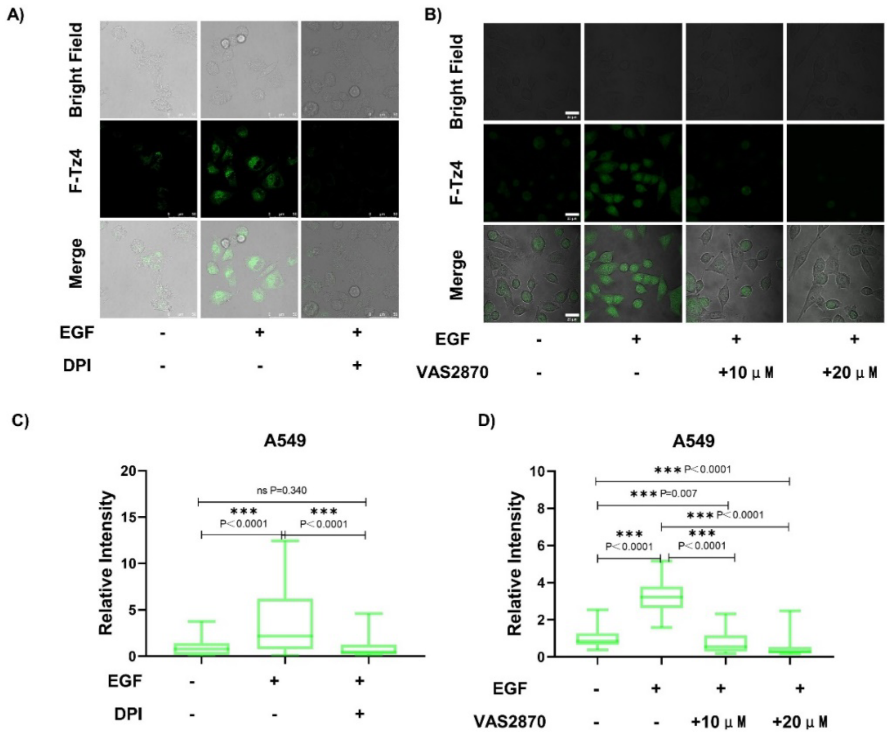

Supplementary Figure 41. F-Tz4 imaging of endogenous superoxide in live A549 cells stimulated by EGF. A) Confocal microscopy images of A549 cells first treated with 0.5 μg/mL EGF for 30 min, then stained with **F-Tz4** (5 μM) for 30 min before imaging (Scale bar: 50 μm). The cells were either pretreated with DPI (at a concentration of 5 μM) for 30 min prior to EGF treatment or left untreated as a control. B) Confocal microscopy images of A549 cells treated with 0.5 μg/mL EGF for 30 min, then stained with **F-Tz4** (5 μM) for 30 min before imaging (Scale bar: 25 μm). The cells were either pretreated with VAS2870 (at concentrations of 10 and 20 μM) for 60 min prior to EGF treatment or left untreated as a control. C-D) Quantified relative mean fluorescence intensities of the cells. P values were analyzed by two-tailed unpaired t-test, 95% Confidence interval. C: n =45 cells for without treatment group, n =89 cells for EGF group, n =58 cells for DPI group; D: n =113 cells for without treatment group, n =109 cells for EGF group, n =72 cells for 10 μM VAS2870 group, n =85 cells for 20 μM VAS2870 group. All cell numbers are over 3 indenpendent experiments. For the boxplots, the top and bottom lines of each box represent the 75th and 25th percentiles of the samples, respectively. The line inside each box represents the median of the samples. The upper and lower lines above and below the boxes are the whiskers. ns: not significant, ***$P < 0.001$ versus untreated cells.

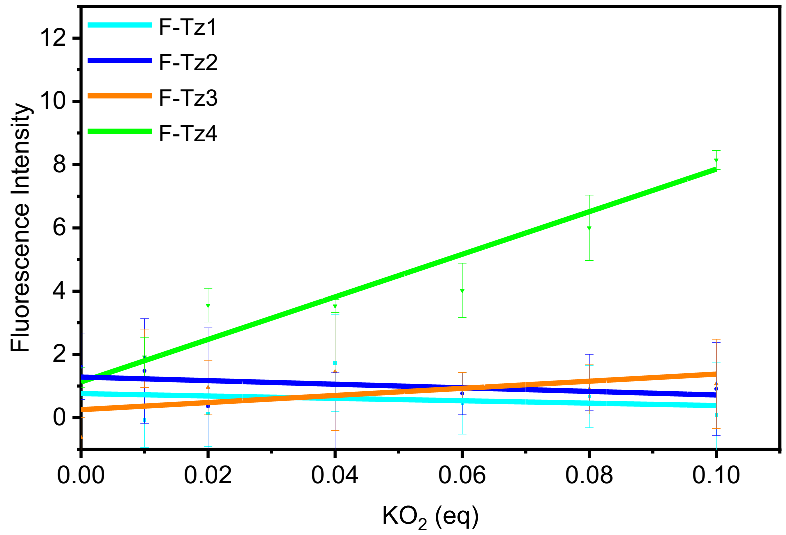

Supplementary Figure 42. Plot of probe fluorescence intensity at their maximum emission against superoxide doses. Fluorescence intensity of the probes (5 μM) after the treatment of low doses of superoxide (0-0.1 eq) for 30 min. Data were presented as mean value ± SD, n = 3 independent experiments.

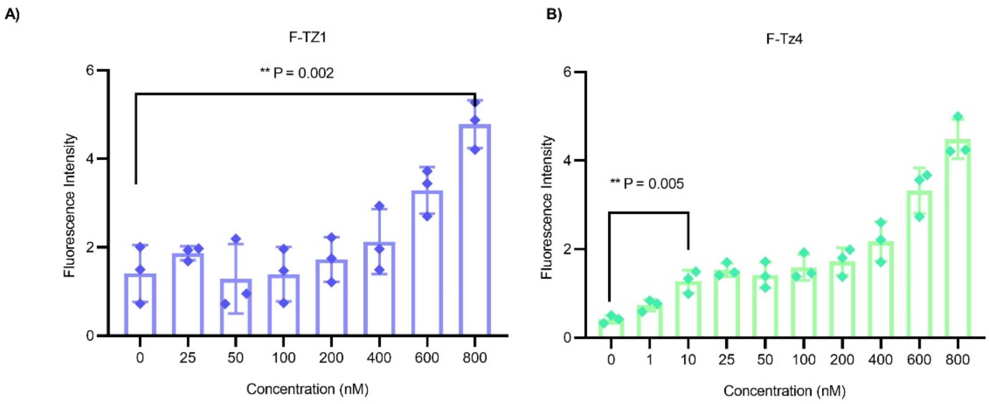

Supplementary Figure 43. Determining the limit of detection (LOD) of probes F-Tz1 and F-Tz4 towards $O_2^{\bullet-}$. Probe (5.0 μM) was treated with various concentrations of $O_2^{\bullet-}$ for 30 min and the fluorescence intensity at their maximum emission was recorded and the detection limit was determined to be the concentration of $O_2^{\bullet-}$ that induced a 3-fold fluorescence increase. Experiments were done in triplicate. (P values were analyzed with unpaired two-tailed t test, 95% Confidence interval. **P < 0.01 versus probe-blank. Data were presented as mean value ± SD, n = 3 independent experiments.

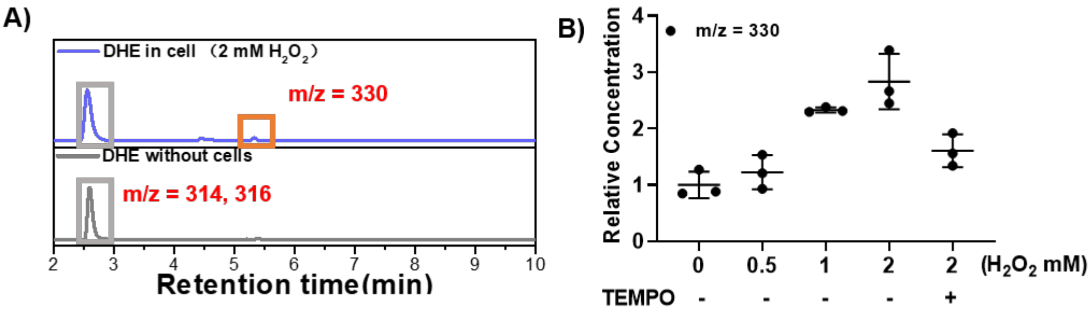

Supplementary Figure 44. Determining cellular superoxide levels by the formation of 2-hydroxyethidium. A) LC traces of DHE in no-cell group (10 µM, PBS) or in cell lysates. B) The quantification of cellular 2-hydroxyethidium with different treatment based on the MS peak area and protein concentration. For the TEMPO group, cells pretreaed with TEMPO were stilulated with H_2O_2 (2 mM) in the presence of TEMPO. Data were presentrd as mean value ± SD, n = 3 indenpendent wells of cells/group.

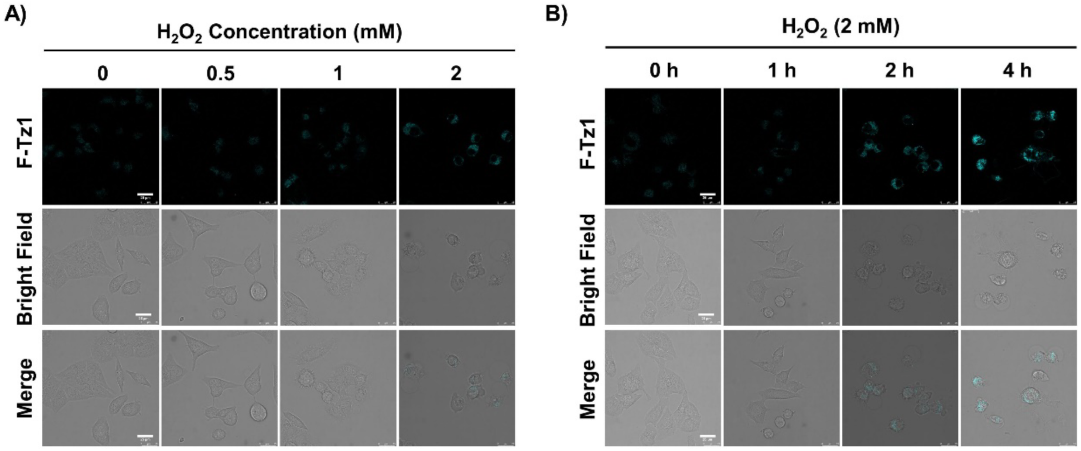

Supplementary Figure 45. Imaging cellular superoxide levels with probe F-Tz1. A) Cells were pretreated with various doses of H_2O_2 for 2 h and then stained with **F-Tz1** (5 µM, 30 min). B) Cells were pretreated with 2 mM H_2O_2 for various times and then stained with **F-Tz1** (5 µM, 30 min). Scale bar: 25 µm. Representative images are shown from n = 3 independent experiments.

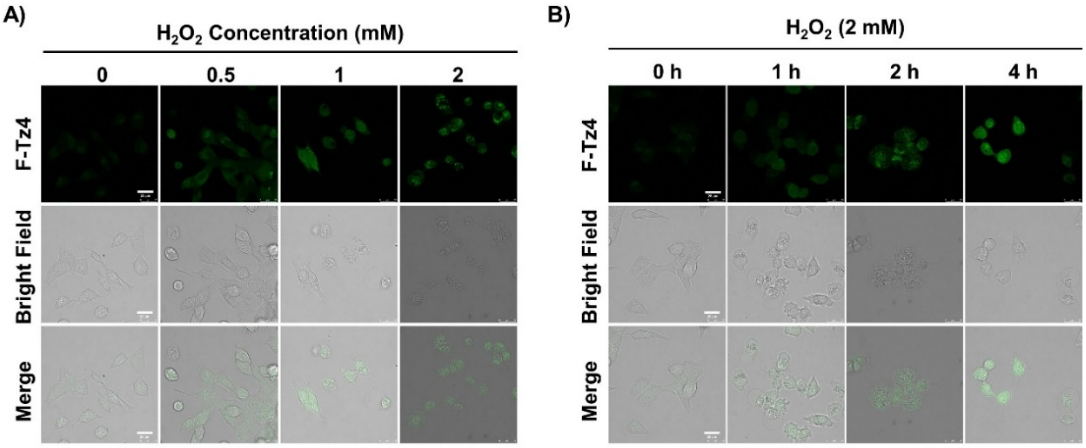

Supplementary Figure 46. Imaging cellular superoxide levels with probe F-Tz4. A) Cells were pretreated with various doses of H_2O_2 for 2 h and then stained with **F-Tz4** (5 µM, 30 min). B) Cells were pretreated with 2 mM H_2O_2 for various times and then stained with **F-Tz4** (5 µM, 30 min). Scale bar: 25 µm. Representative images are shown from n = 3 independent experiments.

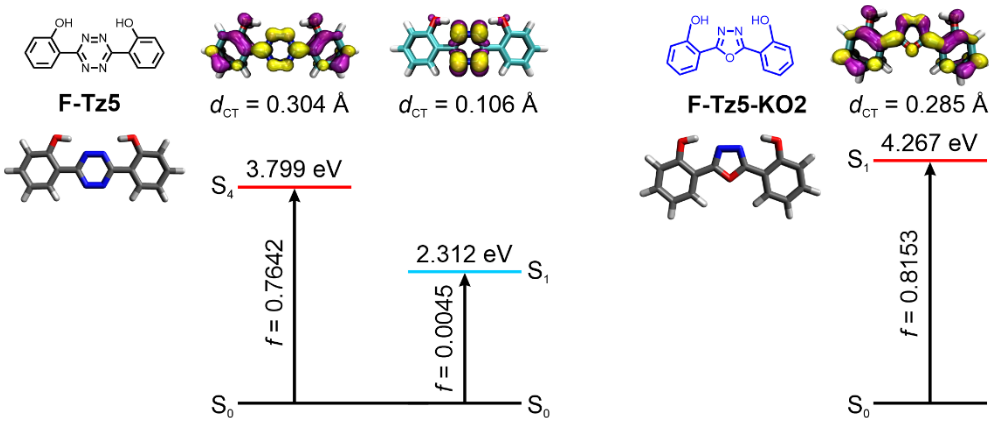

Supplementary Figure 47. Quantum chemical calculations of F-Tz5. Molecular structures, optimized geometries, and electron-hole distributions of the selected vertical excited states of **F-Tz5** before (the left panel) and after reactions (the right panel) with superoxide. The insets show the vertical excitation energy, corresponding oscillator strength (f), and charge transfer distance (d_{CT}) of these states.

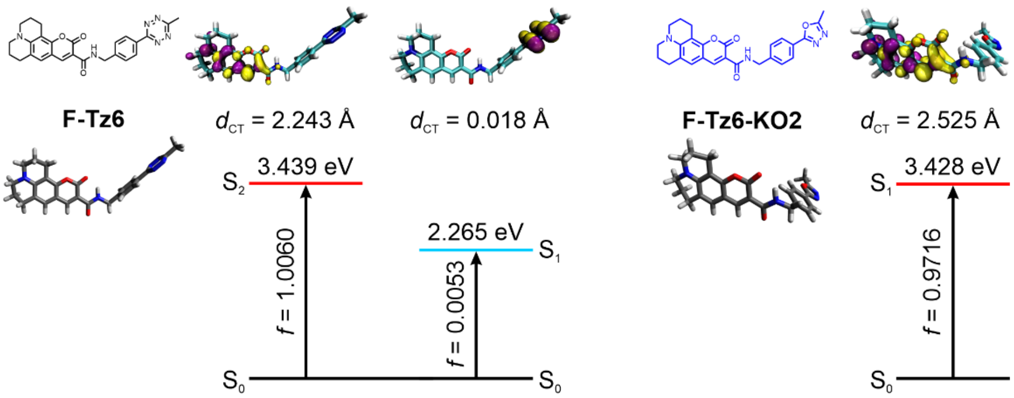

Supplementary Figure 48. Quantum chemical calculations of F-Tz6. Molecular structures, optimized geometries, and electron-hole distributions of the selected vertical excited states of **F-Tz6** before (the left panel) and after reactions (the right panel) with superoxide. The insets show the vertical excitation energy, corresponding oscillator strength (f), and charge transfer distance (d_{CT}) of these states.

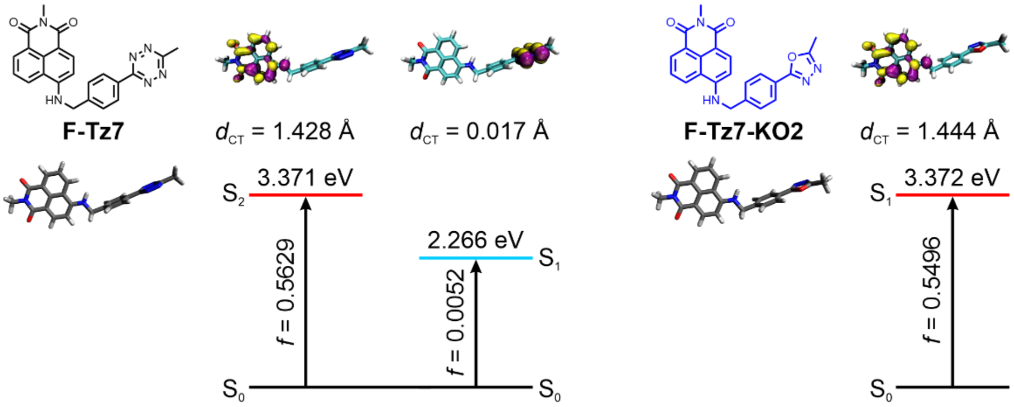

Supplementary Figure 49. Quantum chemical calculations of F-Tz7. Molecular structures, optimized geometries, and electron-hole distributions of the selected vertical excited states of **F-Tz7** before (the left panel) and after reactions (the right panel) with superoxide. The insets show the vertical excitation energy, corresponding oscillator strength (f), and charge transfer distance (d_{CT}) of these states.

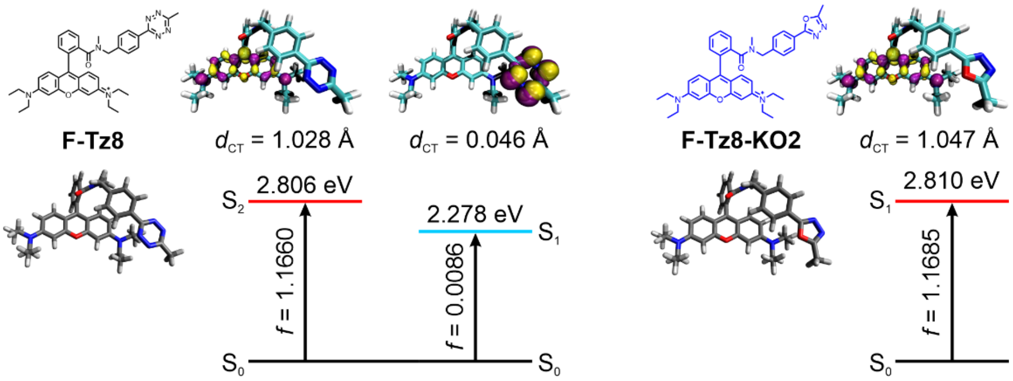

Supplementary Figure 50. Quantum chemical calculations of F-Tz8. Molecular structures, optimized geometries, and electron-hole distributions of the selected vertical excited states of **F-Tz8** before (the left panel) and after reactions (the right panel) with superoxide. The insets show the vertical excitation energy, corresponding oscillator strength (f), and charge transfer distance (d_{CT}) of these states.

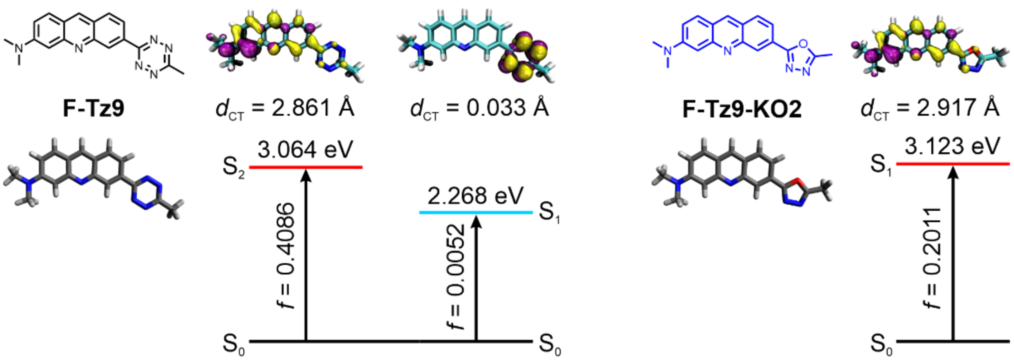

Supplementary Figure 51. Quantum chemical calculations of F-Tz9. Molecular structures, optimized geometries, and electron-hole distributions of the selected vertical excited states of **F-Tz9** before (the left panel) and after reactions (the right panel) with superoxide. The insets show the vertical excitation energy, corresponding oscillator strength (f), and charge transfer distance (d_{CT}) of these states.

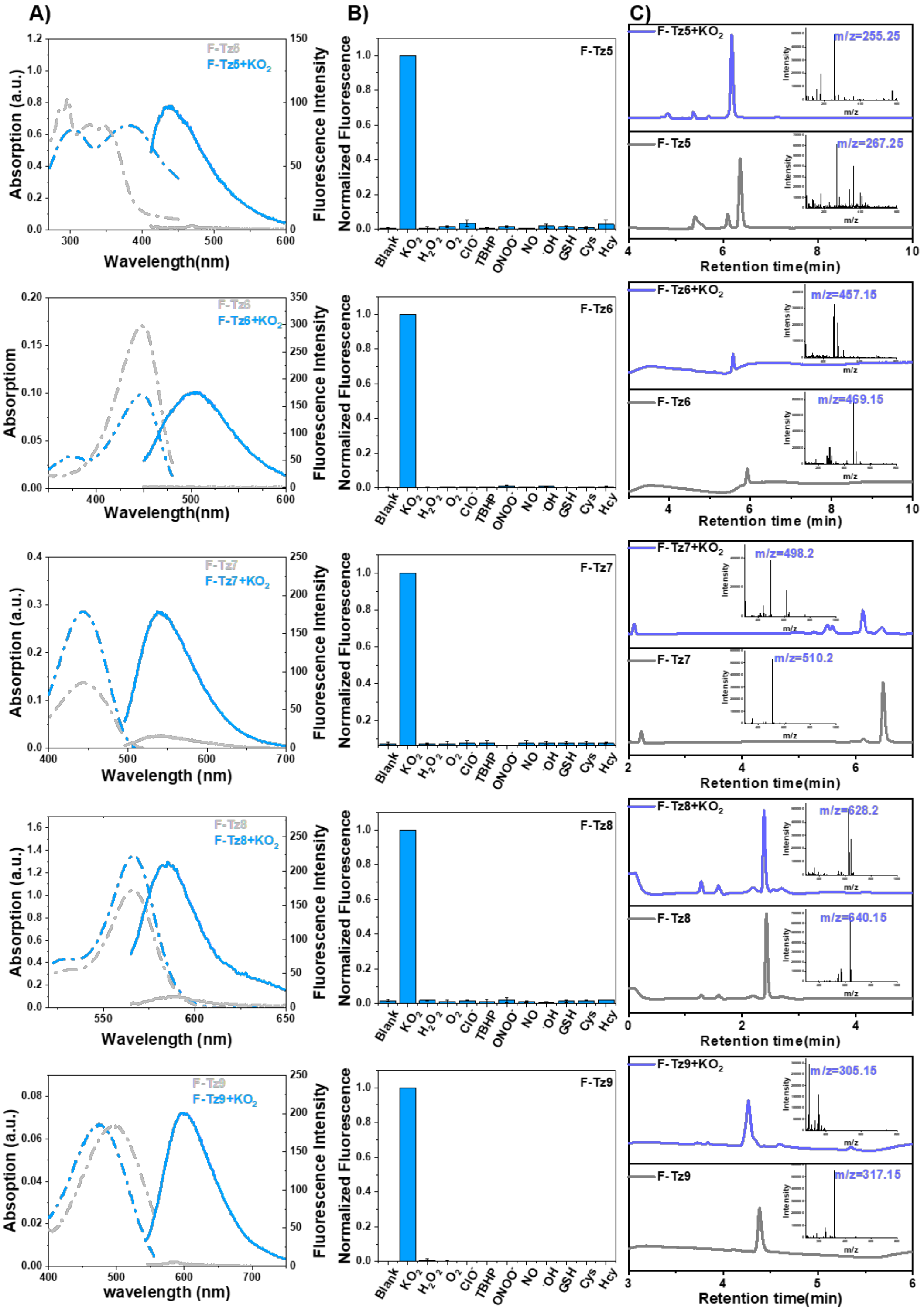

Supplementary Figure 52. Extension of Tz to other fluorophores to tune probe emission color. A) UV-Vis (dotted) and fluorescence (solid) spectra of the probes before or after the treatment of superoxide. B) Normalized fluorescence intensity at their maximum emission of the probes before or after the treatment of various analytes. Data were presented as mean value ± SD, n = 3 independent experiments. C) LCMS traces of probe before and after the treatment of 20 eq KO_2. The probe was used at 100 μM, and treated with 20 eq KO_2 in acetonitrile for 30 min. The sample was analyzed by LCMS, with the mass spectra of the main peak shown in the inner panel.

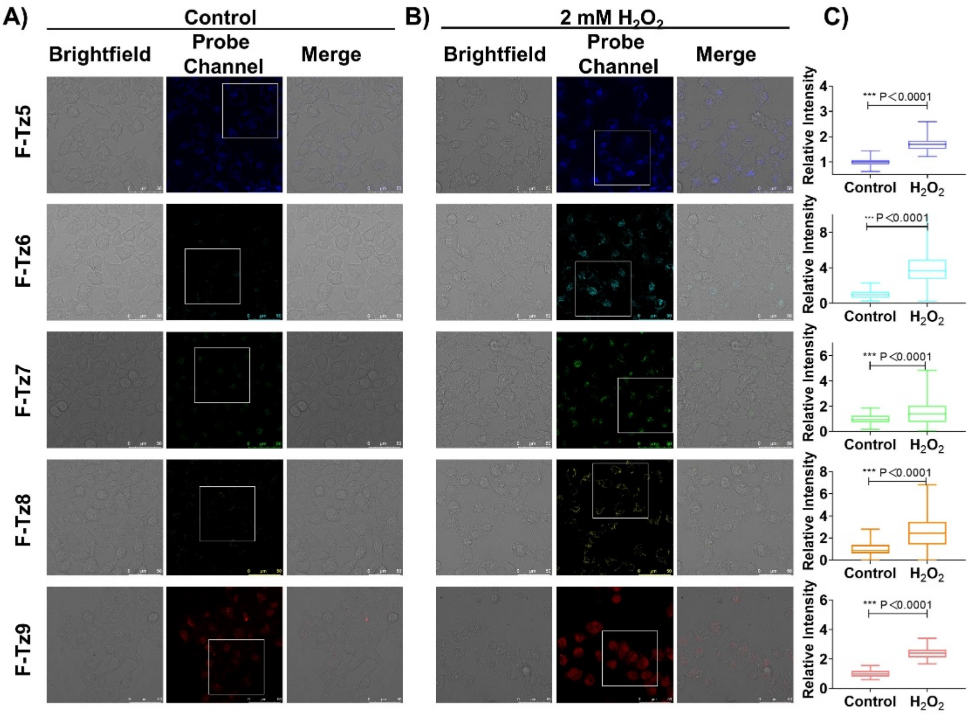

Supplementary Figure 53. Imaging superoxide in live cells with probe F-Tz5 - F-Tz9. A) Cells were stained with **F-Tz5** – **F-Tz8** (5 µM, 30 min), or **F-Tz9** (5 µM, 4 h). The selected area in the white box is the display area in Supplementary Figure 4D. B) Cells were pretreated with 2 mM H_2O_2 for 2 h (F-Tz9: 4 h) and then stained with **F-Tz5** – *F-Tz8* (5 µM, 30 min) (**F-Tz9**: 4 h). Scale bar: 50 µm. The selected area in the white box is the display area in Supplementary Figure 4D. C) Quantified relative mean fluorescence intensity of the cells. P values were analyzed by two-tailed unpaired t-test, 95% Confidence interval. **F-Tz5**: n =66 cells for control group, n =55 cells for H_2O_2 group; **F-Tz6**: n =66 cells for control group, n =56 cells for H_2O_2 group; **F-Tz7**: n =90 cells for control group, n =127 cells for H_2O_2 group; **F-Tz8**: n =235 cells for control group, n =141cells for H_2O_2 group; **F-Tz9**: n =62 cells for control group, n =49 cells for H_2O_2 group. All cell numbers are over 3 indenpendent experiments. For the boxplots, the top and bottom lines of each box represent the 75th and 25th percentiles of the samples, respectively. The line inside each box represents the median of the samples. The upper and lower lines above and below the boxes are the whiskers. ***P < 0.001 versus untreated cells.

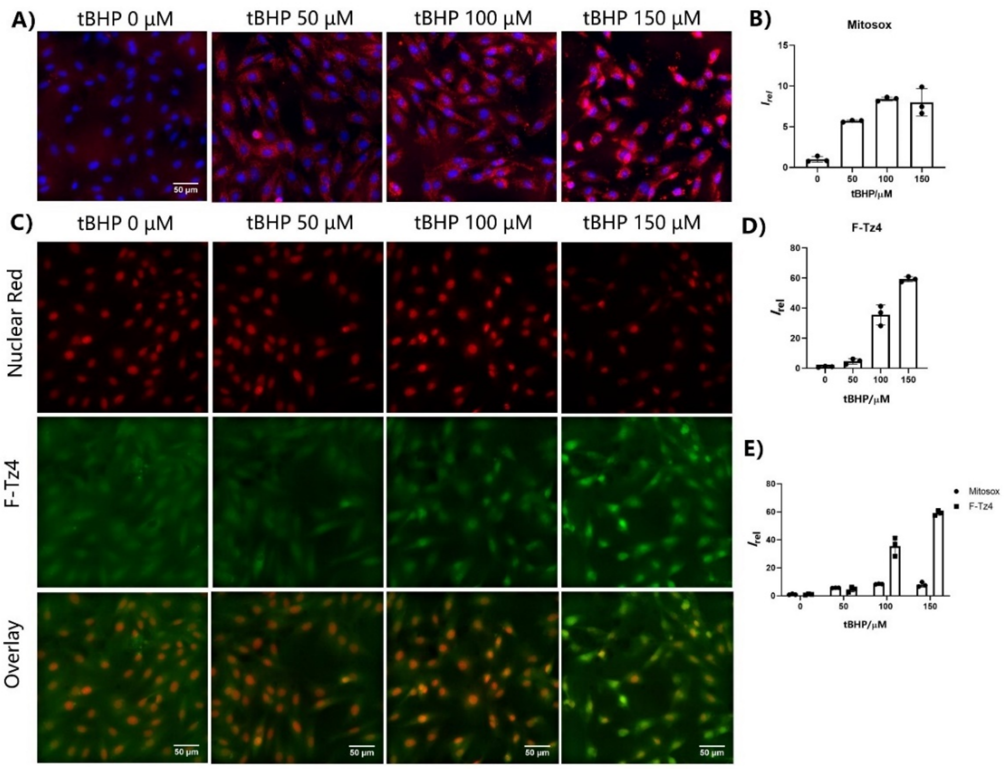

Supplementary Figure 54. Imaging *t*BHP-induced cellular superoxide in H9C2 cells with Mitosox or F-Tz4. A) Fluorescence images of H9C2 cells stained with Mito Sox (1 μM, 30 min). Before being stained with Mito Sox, cells were intact (control), or pretreated with 50, 100, or 150 μM *t*BHP for 2 h. B) The statistically quantified data on the cellular fluorescence intensity in A. The data were the mean ± SD and were normalized to the control group. C) Fluorescence images of H9C2 cells co-stained with Nuclear Red (1 μM, 10 min) and **F-Tz4** (1 μM, 30 min). Before being stained with the probes, cells were intact (control), or pretreated with 50, 100, or 150 μM *t*BHP for 2 h. D) The statistically quantified data on the cellular fluorescence intensity in C. The data were the mean ± SD and were normalized to the control group. E) Comparison of Mito Sox and F-Tz4 staining results. The statistically quantified data on the cellular fluorescence intensity in A and C. The data were the mean ± SD and were normalized to the control group. n = 3 independent wells of cells. Scale bar: 50 μm.

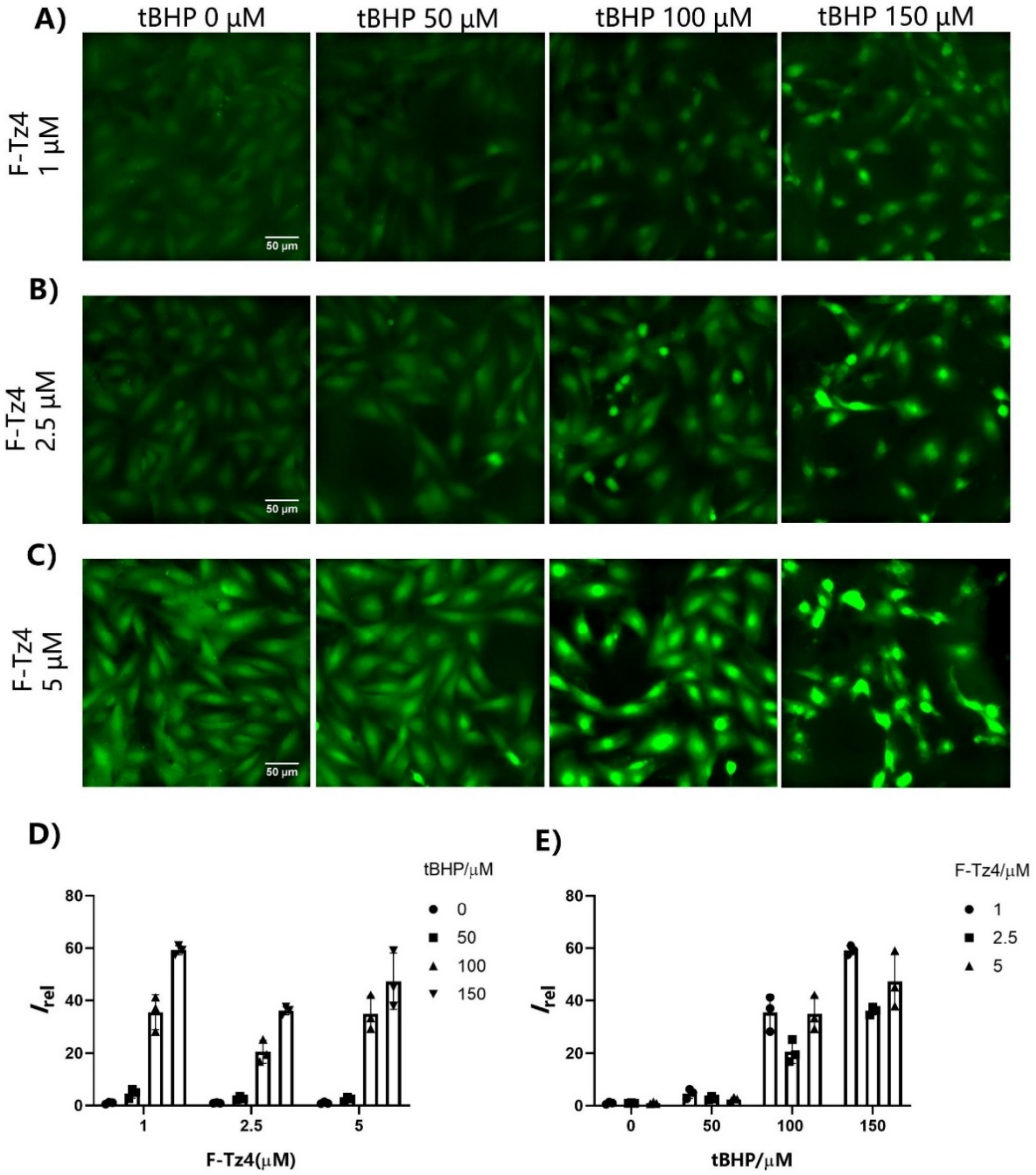

Supplementary Figure 55. Optimization of the concentrations of *t*BHP and F-Tz4 for the screen model. A-C) Fluorescence images of H9C2 cells stained with 1, 2.5, 5 μM of **F-Tz4** for 30 min respectively. Before staining, cells were intact (control), or pretreated with 50, 100 or 150 μM tBHP for 2 h. Scale bar: 50 μm. Data shown were mean ±SD, n = 3 independent wells of cells /group. D) The statistically quantified data on the cellular fluorescence intensity in A-C. The data were the mean ± SD and were normalized to the control group. n = 3 independent wells of cells/group. E) The statistical results of parallel triplicates of experiments in A-C. All data were the mean ± SD and were normalized to the control group.

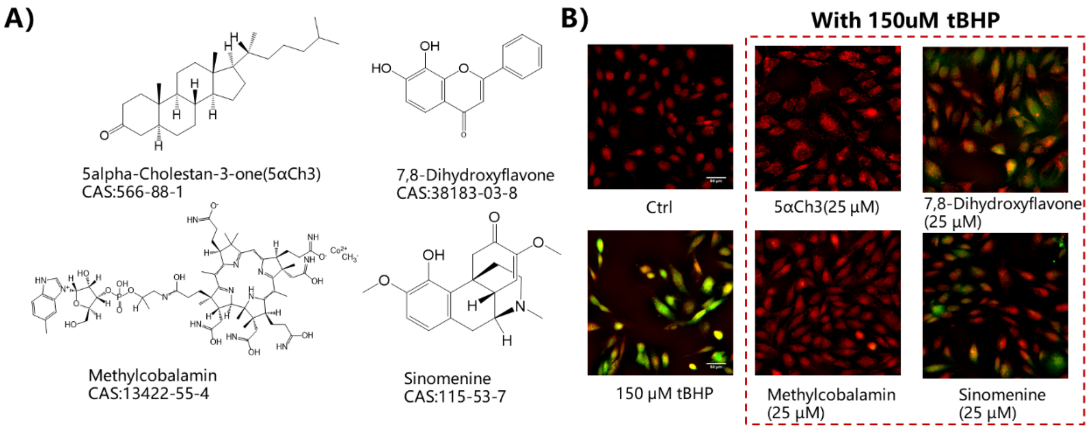

Supplementary Figure 56. Structures of the lead compounds screened out by the model, and their effects on decreasing cellular superoxide overexpression as indicated by F-Tz4 staining. N = 3 independent wells of cells/group. Scale bar: 50 μm.

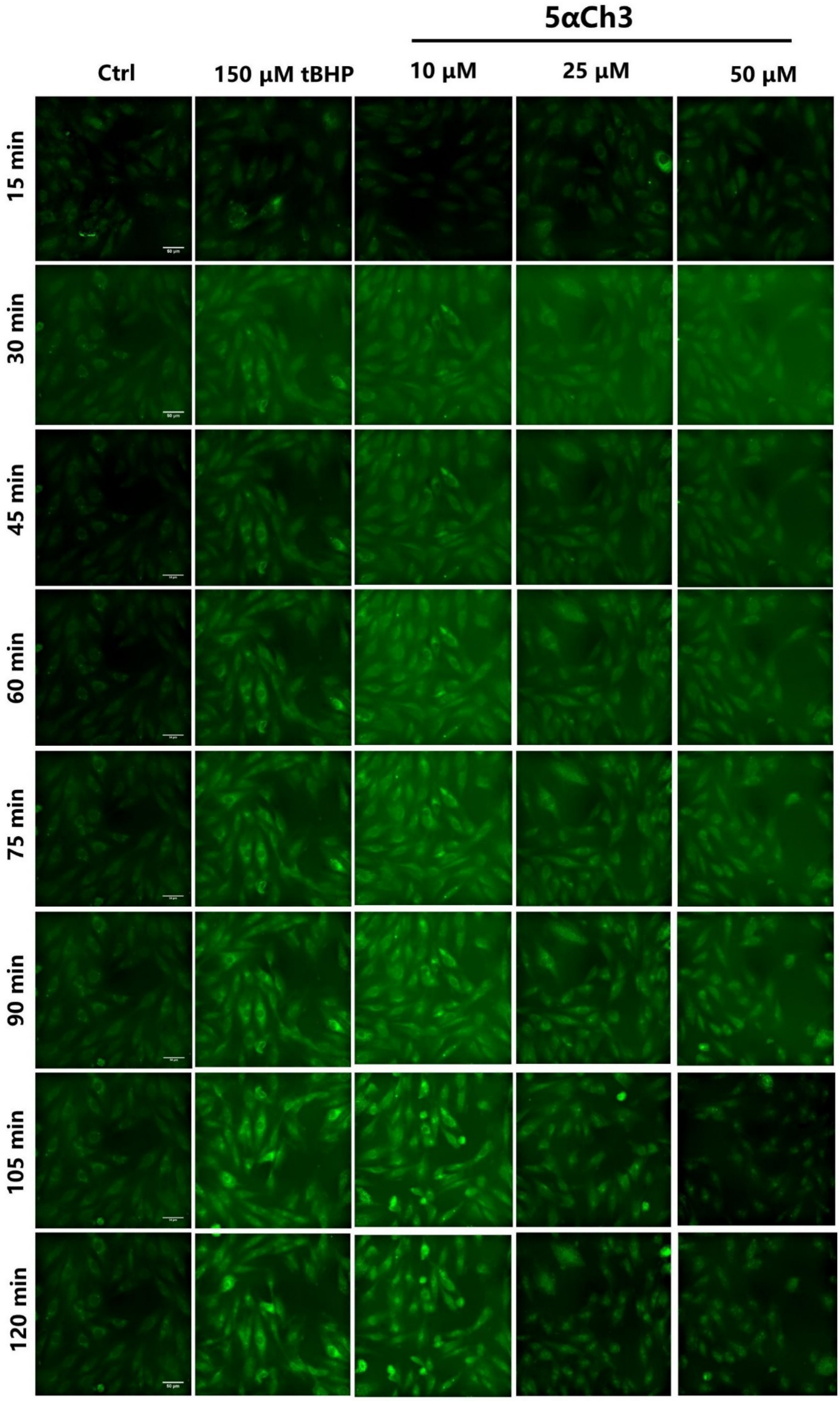

Supplementary Figure 57. Representative time-lapsed images of H9C2 cells co-treated with *t*BHP and F-Tz4 (1 μM). Cells were intact (control and model) or pretreated with coprostanone (10, 25, 50

μM) for 24 h and all groups then treated with *t*BHP (150 μM) except the cells in the control group. At the same time, all cells were co-treated with the **F-Tz4**. n = 3 independent wells/group. Scale bar: 50 μm.

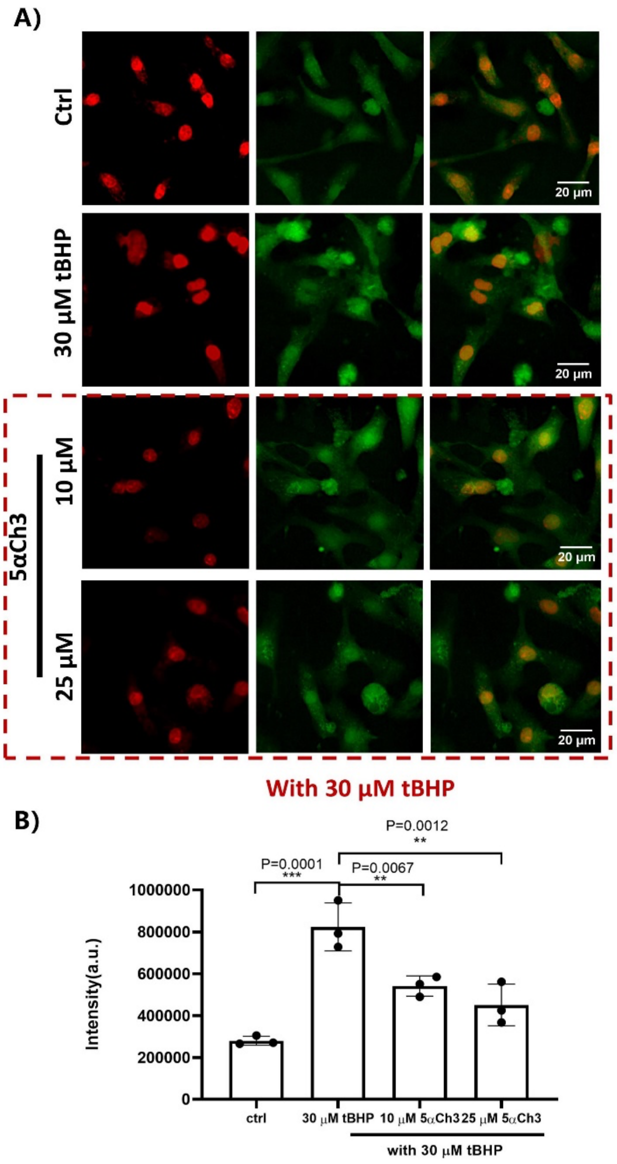

Supplementary Figure 58. Protective effect of 5αCh3 on *t*BHP-injured primary cardiomyocytes. A) Fluorescence images of primary cardiomyocytes cells stained with 10 μM of **F-Tz4** for 30 min respectively. Before staining, cells were intact (control) or pretreated with coprostanone (10 or 25 μM) for 24 h. All groups were then treated with *t*BHP (30 μM) except the cells in control group for 2 h, and then stained with F-Tz4 for 30 min. Images were acquired using an ImageXpress Micro Confocal High-Content Imaging System (Molecular Devices), with a 40 × PlanFluor objective. Scale bar: 20 μm. B) The statistically quantified data on the cellular fluorescence intensity in A. The data were the mean ± SD and were normalized to the control group. n = 3 independent wells of cells/group. All data were analyzed using one-way ANOVA with Dunnett's multiple comparisons test and data were expressed as means ± SD, **P < 0.01, ***P < 0.001, versus *t*BHP group.

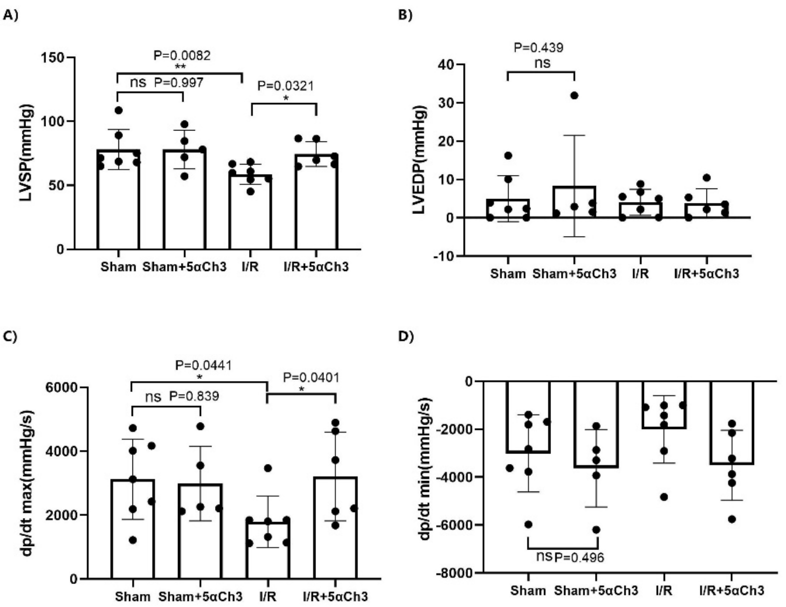

Supplementary Figure 59. Hemodynamic parameters in mice subjected to cardiac I/R injury with 5αCh3 pretreatment. (A) LVSP, left ventricular systolic pressure; (B) LVEDP, left ventricular end diastolic pressure; (C) dp/dt max: the maximum rate of left ventricular pressure change; (D) dp/dt min: the minimum rate of left ventricular pressure change (n = 7 mice for Sham and I/R group, n = 5 mice for Sham +5αCh3 group and n = 6 mice for I/R+5αCh3 group). Data were expressed as mean ± SD and analyzed with one-way ANOVA followed by uncorrected Fisher's LSD multiple comparisons test. *P < 0.05, ** P < 0.01 vs. I/R group.

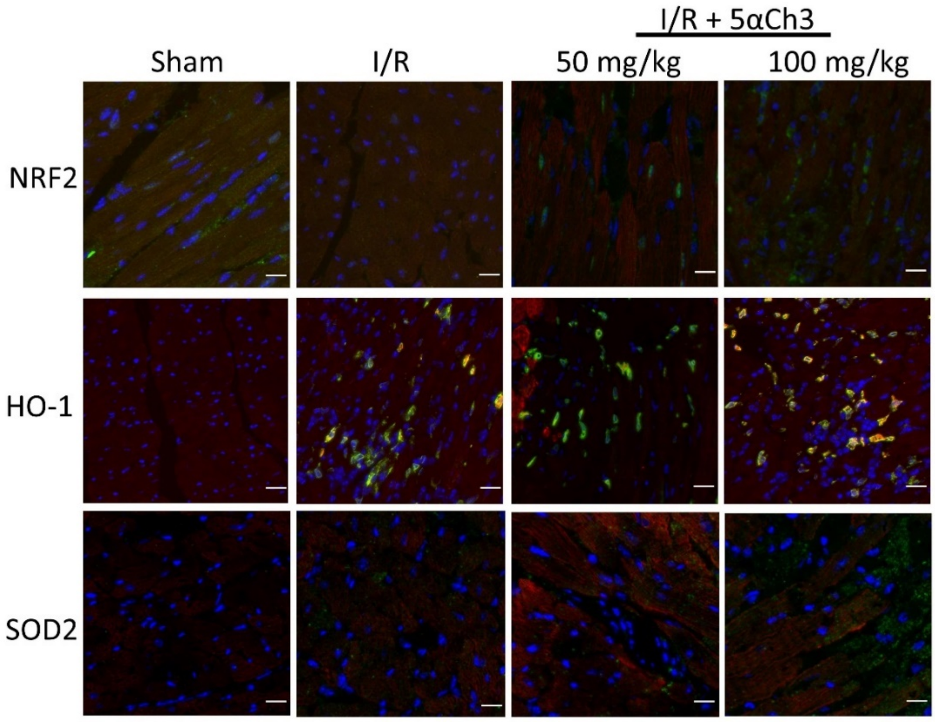

Supplementary Figure 60. Representative images of immunofluorescence staining of NRF2, HO-1 and SOD2 in the heart tissues of mice in sham operation group, I/R group, 5αCh3 low dose group (50 mg/kg), 5αCh3 high dose group (100 mg/kg) respectively (n = 4 mice for each group). Green represents the target protein, blue represents the nucleus, and red represents cTnT in these images. Scale bar = 20 μm.

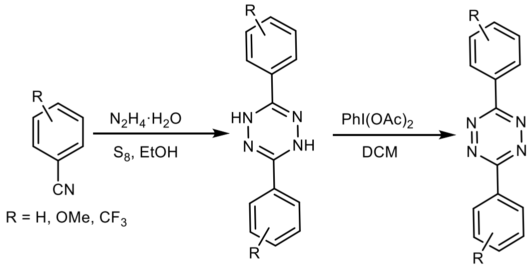

Supplementary Figure 61. General procedures (A) for synthesizing symmetric tetrazines.

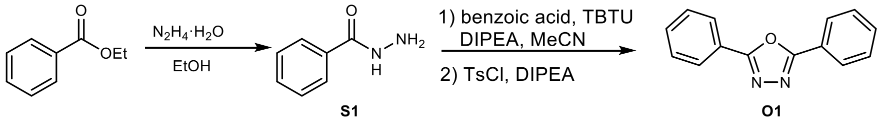

Supplementary Figure 62. Synthesis of 2,5-diphenyl-1,3,4-oxadiazole (O1).

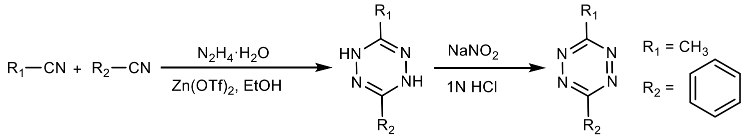

Supplementary Figure 63. General procedures (B) for synthesizing asymmetric tetrazines.

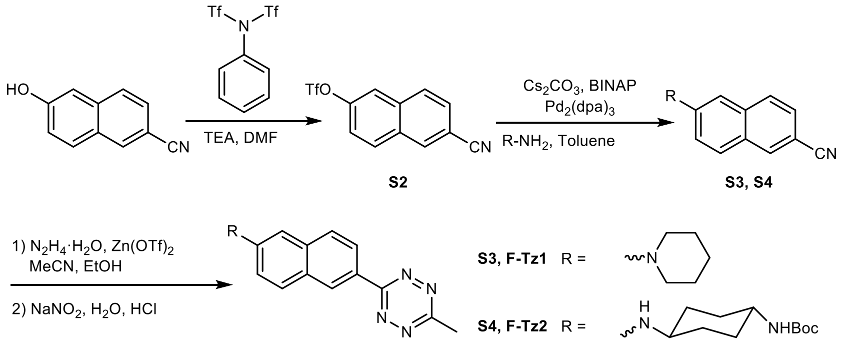

Supplementary Figure 64. Synthesis of probes F-Tz1 and F-Tz2.

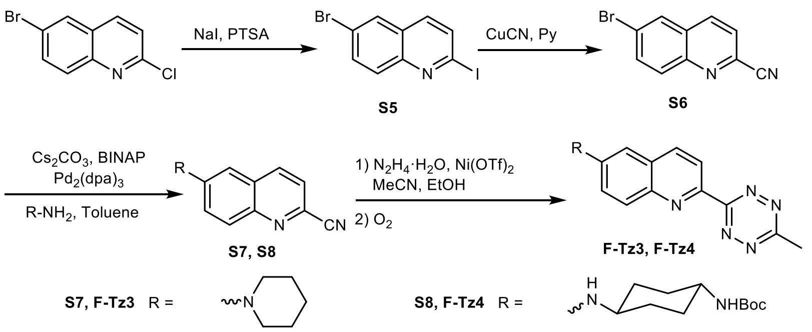

Supplementary Figure 65. Synthesis of probes F-Tz3 and F-Tz4.

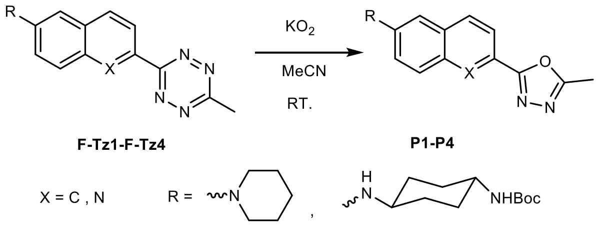

Supplementary Figure 66. Synthesis of oxadiazole derivatives of F-Tz1-F-Tz4 (P1-P4).

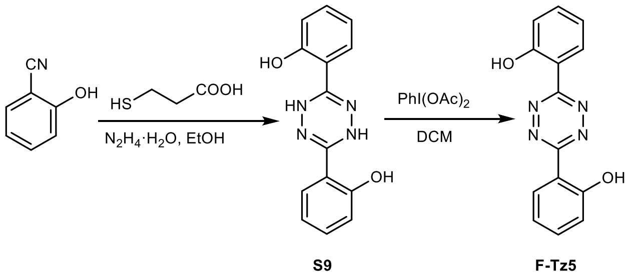

Supplementary Figure 67. Synthesis of probe F-Tz5.

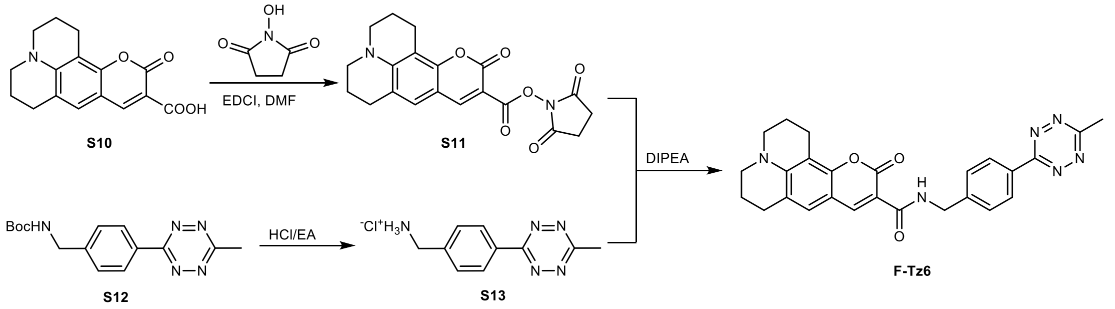

Supplementary Figure 68. Synthesis of probe F-Tz6.

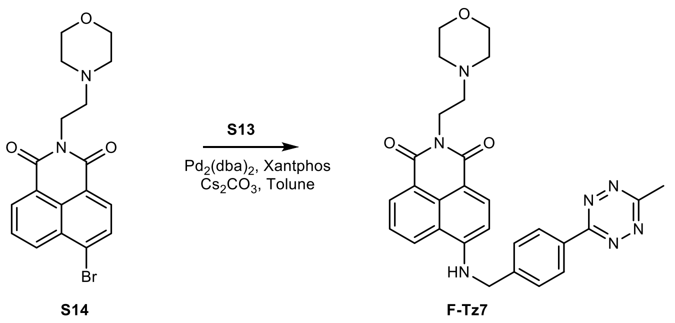

Supplementary Figure 69. Synthesis of probe F-Tz7.

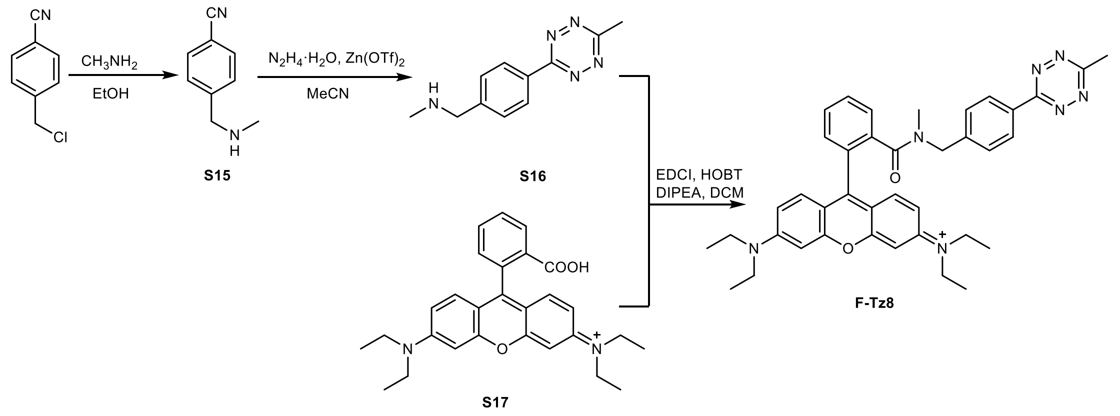

Supplementary Figure 70. Synthesis of probe F-Tz8.

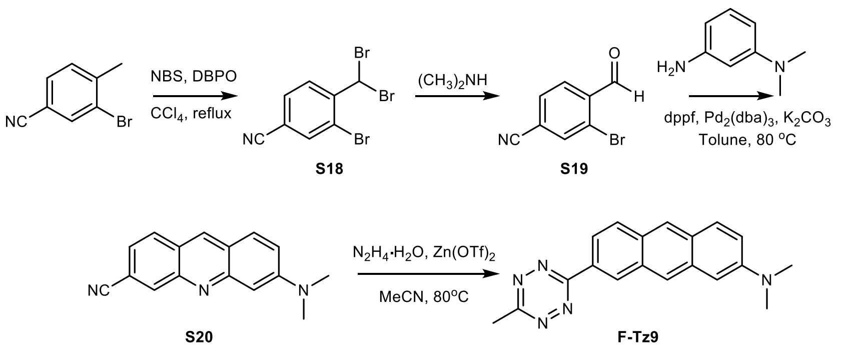

Supplementary Figure 71. Synthesis of probe F-Tz9.

General chemistry methods

All chemicals were from commercial supplies and used without further purification except otherwise indicated. ^{1}H NMR spectra were recorded on a Bruker 500 Fourier transform spectrometer (500 MHz). ^{13}C NMR spectra were obtained on a Bruker 500 Fourier transform spectrometer (126 MHz) spectrometer. Chemical shifts (δ) for ^{1}H and ^{13}C NMR spectra were given in ppm. The residual solvent signals were used as references for ^{1}H and ^{13}C NMR spectra and the chemical shifts were converted to the TMS scale ($CDCl_3$, 7.26 ppm for ^{1}H NMR and 77.16 ppm for ^{13}C NMR; CD_3OD, 3.31 ppm for ^{1}H NMR and 49.00 ppm for ^{13}C NMR; $(CD_3)_2SO$, 2.50 ppm for ^{1}H NMR and 39.52 ppm for ^{13}C NMR). All chemical shifts were reported in parts per million (ppm) and coupling constants (J) in Hz. The following abbreviations were used to explain the multiplicities: d = doublet, t = triplet, m = multiplet, dd = doublet of doublets. High-resolution mass spectra (HRMS) for new compounds were measured on an Agilent 6224 TOF LC/MS spectrometer using ESI-TOF (electrospray ionization-time of flight). Liquid chromatography equipped with a low resolution mass detector (LC-MS) was conducted on a SHIMADZU LCMS-2020 spectrometer. High Performance Liquid Chromatography (HPLC) was measured on an Agilent Technologies 1260 Infinity system. Fluorescence spectra were measured on an Agilent Cary Eclipse Fluorescence Spectrophotometer. Absorption spectra were collected using a Hitachi U-3010 spectrophotometer. Cyclic voltammograms were measured on an Shanghai YueCi CHI660E Electrochemical workstation. The confocal cell imaging experiments were carried out on a Leica TCS SP8 live cell fluorescence microscope. High-content screening was conducted on an ImageXpress Micro Confocal High-Content Imaging System.

Synthetic procedures and structure characterization.

General procedures (A) for synthesizing symmetric tetrazines (Supplementary Figure S61)[1]

Anhydrous ethanol (3.00 mL), nitrile (1.00 g), and sulfur (0.200 g) were added to a two-neck flask, and the apparatus was flushed with a nitrogen atmosphere. Hydrazine hydrate (80%, 2.00 mL) was added dropwise to the solution at 0 °C. And the resulting mixture was allowed to stir for 2 h while warming to room temperature. The mixture was then refluxed overnight, cooled to 0 °C, and filtered. The filtered solid was rinsed with cold ethanol (3 × 5 mL) to afford the dihydrotetrazine intermediate, which was used in the subsequent oxidation step without further purification.

Transfer the dihydrotetrazine intermediate to a round-bottomed flask. Dichloromethane (20.0 mL) was added, and the suspended mixture was stirred. To the stirring suspension was added phenyliodonium diacetate (1.50 eq). The reaction mixture was allowed to stir until all ingredients were oxidized as shown by TLC analysis. And then the crude product was purified by silica gel chromatography to yield the tetrazine as a pink solid.

Synthesis of Tz1. Tz1 was synthesized by the general procedure A and was purified as a pink solid after silica column chromatography (PE : DCM = 3 : 2 – 0 : 1).

Yield: 26.5%

Rf = 0.13 (PE : DCM = 3 : 1)

^{1}H NMR (500 MHz, $CDCl_3$): δ 8.68 – 8.63 (m, 4H), 7.66 – 7.59 (m, 6H)

^{13}C NMR (126 MHz, $CDCl_3$): δ 164.09, 132.82, 131.89, 129.45, 128.10

ESI-MS (*m/z*): $[M+H]^+$ calc'd. for $C_{14}H_{11}N_4$ 235.10, found 235.25

Synthesis of Tz2. Tz2 was synthesized by general procedure A and was purified as a pink solid after silica column chromatography (PE : DCM = 2 : 1 – DCM : EA = 10 : 1).

Yield: 13.6%
Rf = 0.13 (PE : DCM = 3 : 1)
^{1}H NMR (500 MHz, $CDCl_3$): δ 8.65 – 8.52 (m, 4H), 7.15 – 7.05 (m, 4H), 3.93 (s, 6H)
^{13}C NMR (126 MHz, $CDCl_3$): δ 163.43, 163.39, 129.75, 124.64, 114.92, 55.67
ESI-MS (*m/z*): $[M+H]^+$ calc'd. for $C_{16}H_{15}N_4O_2$ 295.12, found 295.20

Synthesis of Tz3. Tz3 was synthesized by general procedure A and was purified as a pink solid after silica column chromatography (PE : DCM = 1 : 1– DCM : EA = 2 : 1).
Yield: 49.8%
Rf = 0.57 (PE : DCM = 3 : 1)
^{1}H NMR (500 MHz, $CDCl_3$): δ 8.82 (d, J = 8.0 Hz, 4H), 7.91 (d, J = 7.9 Hz, 4H)
^{13}C NMR (126 MHz, $CDCl_3$): δ 163.47, 134.79, 134.39, 128.46, 126.34, 126.31

Synthesis of 2,5-diphenyl-1,3,4-oxadiazole (O1) (Supplementary Figure S62)[2]

Synthesis of S1[3,4]. Ethanol (10.0 mL), ethyl benzoate (2.00 g, 13.3 mmol), and hydrazine hydrate (80%, 0.75 ml, 16.0 mmol) were added into a 50 ml round-bottomed flask. The mixture was stirred under reflux overnight until consumption of ethyl benzoate as evidenced by TLC analysis. Then the mixture was poured into a mixture of water (50 mL) and DCM (50 mL). The organic phase was washed twice with 50 ml water, once with 50 mL brine, and dried over Na_2SO_4. The solvent was evaporated to afford benzohydrazide as a white solid (1.60 g, 89.0%). And the crude product was used in the subsequent step without further purification.
Rf = 0.13 (DCM : MeOH = 20 : 1)
^{1}H NMR (500 MHz, $CDCl_3$) δ 8.09 (s, 1H), 7.78 – 7.72 (m, 2H), 7.52 – 7.46 (m, 1H), 7.43 – 7.37 (m, 2H), 4.16 (s, 2H).
^{13}C NMR (126 MHz, $CDCl_3$) δ 168.81, 132.69, 131.95, 128.76, 127.01.
ESI-MS (*m/z*): $[M+H]^+$ calc'd. for $C_7H_9N_2O$ 137.07, found 137.35

Synthesis of O1. To a mixture of benzoic acid (708 mg, 5.80 mmol), benzohydrazide (800 mg, 5.80 mmol), and diisopropylethylamine (2.20 g, 17.4 mmol) in acetonitrile (20.0 ml) at room temperature was added TBTU (2.00 g, 6.40 mmol). The mixture was stirred for 5 h. Diisopropylethylamine (1.40 g, 11.6 mmol) was successively added, followed by 4-methylbenzenesulfonyl chloride (3.30 g, 17.4 mmol). The resulting reaction mixture was stirred for 12 h. Then it was poured into a 14% NH_3 aqueous solution. The crude mixture was stirred at room temperature for 20 min, then it was extracted with dichloromethane. The organic solution was washed with water, dried over anhydrous Na_2SO_4, filtered and concentrated upon rotary evaporation. The crude product was purified by silica gel chromatography (PE : EA = 5 : 1) to yield O1 as a white solid (1.00 g, 78.0%).
Rf = 0.47 (PE : EA = 5 : 1)
M. P.: 138-140 °C
^{1}H NMR (500 MHz, $CDCl_3$): δ 8.18 – 8.12 (m, 4H), 7.58 – 7.50 (m, 6H)
^{13}C NMR (126 MHz, $CDCl_3$) : δ 164.64, 131.80, 129.15, 127.00, 124.00.
ESI-MS (*m/z*): $[M+H]^+$ calc'd. for $C_{14}H_{11}N_2O$ 223.09, found 223.20

General procedures (B) for synthesizing asymmetric tetrazines (Supplementary Figure S63)[5]

To a high-pressure reaction tube equipped with a stir bar was added 5.0 mL of anhydrous EtOH, 0.500

mmol of $Zn(OTf)_2$, 10.0 mmol of R_1CN, 1.00 mmol of R_2CN, and hydrazine hydrate (80%,, 50.0 mmol). The vessel was sealed and the mixture was stirred in an oil bath at 60 °C for 24 h. The reaction solution was cooled to room temperature and the seal was removed. Sodium nitrite (7.00 mmol) in 5.00 mL of water was slowly added to the solution, followed by the slow addition of 1 N HCl during which the solution turned bright red in color and gas evolved. The addition of HCl was continued until gas evolution ceased and the pH value reached 2-3. (Caution! This step generates a large amount of toxic nitrogen oxide gasses and should be performed in a well-ventilated fume hood). The mixture was extracted with EtOAc and the organic phase was dried over sodium sulfate. The EtOAc was removed using rotary evaporation and the residue was purified using silica column chromatography.

Synthesis of Tz4[6]. Tz4 was synthesized by general procedure B and was purified as a pink solid after silica column chromatography (PE : EA =5 : 1 – 2 :1).

Yield: 11.0%

Rf = 0.60 (PE : EA = 5 : 1)

^{1}H NMR (500 MHz, $CDCl_3$): δ 8.64 – 8.50 (m, 2H), 7.66 – 7.51 (m, 3H), 3.09 (s, 3H)

^{13}C NMR (126 MHz, $CDCl_3$) : δ 167.39, 164.24, 132.68, 131.91, 129.36, 128.04, 21.29.

ESI-MS (*m/z*): $[M+H]^+$ calc'd. for $C_9H_9N_4$ 173.08, found 173.35

Synthesis of probes F-Tz1 and F-Tz2 (Supplementary Figure S64)

Synthesis of S2[7]. 6-Hydroxy-2-naphthonitrile (1.00 g, 5.95 mmol) was dissolved in 5.00 mL DMF. *N*, *N* -bis(trifluoromethanesulfonyl)aniline (2.55 g, 7.13 mmol) and TEA (1.20 g, 11.9 mmol) were added to the solution. The mixture was stirred at room temperature for 6 h under a N_2 atmosphere. The mixture was poured into a mixture of water (50 mL) and ethyl acetate (25 mL). The organic phase was washed 5 times with water, once with brine, and dried over $NaSO_4$. The solvent was evaporated and the crude product was purified by silica gel chromatography (PE : EA = 10 : 1) to yield **S2** as a white solid (1.70 g, 95.5%).

Rf = 0.53 (PE : EA = 5 : 1)

^{1}H NMR (500 MHz, $CDCl_3$): δ 8.30 (s, 1H), 8.03 (d, *J* = 9.0 Hz, 1H), 7.99 (d, *J* = 8.5 Hz, 1H), 7.83 (d, *J* = 2.4 Hz, 1H), 7.73 (dd, *J* = 8.6, 1.6 Hz, 1H), 7.52 (dd, *J* = 9.0, 2.4 Hz, 1H).

^{13}C NMR (126 MHz, $CDCl_3$) : δ 149.14, 134.98, 134.09, 131.51, 131.35, 129.57, 128.25, 121.80, 119.75, 118.89 (q, *J* = 121.0 Hz), 111.18.

Synthesis of S3. A mixture of **S2** (800 mg, 2.66 mmol), piperidine (451 mg, 5.30 mmol), $Pd_2(dba)_3$ (146 mg, 0.16 mmol), BINAP (198 mg, 0.32 mmol) and Cs_2CO_3 (2.16 g, 6.65 mmol) was dissolved in 15.0 mL of dry toluene, and the reaction mixture was stirred under reflux overnight under N_2 atmosphere. Upon consumption of **S2** as evidenced by TLC analysis, the solvent was evaporated and the crude product was extracted with EtOAc and the organic phase was washed 3 times with water, once with brine, and dried over Na_2SO_4. The crude product was used in the subsequent step without further purification.

Rf = 0.67 (PE : EA = 5 : 1)

M. P.: 82-84 °C

IR (KBr): 3031, 2994, 2857, 2222 cm^{-1}

ESI-HRMS (*m/z*): $[M+H]^+$ calc'd. for $C_{16}H_{17}N_2$ 237.1392, found 237.1338

Synthesis of S4. A mixture of **S2** (1.00 g, 3.32 mmol), *trans*-*N*-Boc-1,4-cyclohexanediamine (1.07 g, 4.97 mmol), $Pd_2(dba)_3$ (183 mg, 0.200 mmol), BINAP (248 mg, 0.400 mmol) and Cs_2CO_3 (2.70 g, 8.30

mmol) was dissolved in 15.0 mL of dry toluene. The reaction mixture was stirred under reflux overnight under a N_2 atmosphere. Upon consumption of **S2** as evidenced by TLC analysis, the solvent was evaporated and the crude product was purified by silica gel chromatography (PE : EA = 2 : 1) to yield **S4** as a white solid (500 mg, 41.7%).

Rf = 0.13 (PE : EA = 5 : 1)

M. P.: 160-162 °C

IR (KBr): 3376, 3353, 3057, 3977, 2934, 2857, 2218, 1680, 1620, 1523, 1169 cm^{-1}

^{1}H NMR (500 MHz, $CDCl_3$): δ 7.98 (d, J = 1.5 Hz, 1H), 7.61 (d, J = 8.9 Hz, 1H), 7.58 (d, J = 8.6 Hz, 1H), 7.43 (dd, J = 8.5, 1.7 Hz, 1H), 6.88 (dd, J = 8.9, 2.3 Hz, 1H), 6.72 (d, J = 2.4 Hz, 1H), 4.54 – 4.41 (m, 1H), 4.07 (s, 1H), 3.51 (s, 1H), 3.42 – 3.33 (m, 1H), 2.35 – 2.16 (m, 2H), 2.16 – 1.98 (m, 2H), 1.46 (s, 9H), 1.35 – 1.21 (m, 4H)

^{13}C NMR (126 MHz, $CDCl_3$): δ 155.35, 147.34, 137.20, 133.91, 129.84, 127.20, 126.61, 125.90, 120.35, 119.57, 104.17, 103.88, 79.51, 51.15, 49.33, 32.18, 31.78, 28.55

ESI-HRMS (*m/z*): $[M+H]^+$ calc'd. for $C_{22}H_{28}N_3O_2$ 366.2182, found 366.2191

Synthesis of F-Tz1. To a high pressure reaction tube was added **S3** (200 mg, 0.840 mmol), MeCN (446 µL, 8.47 mmol), $Zn(OTf)_2$ (153 mg, 0.420 mmol) and hydrazine hydrate (80%, 2.10 mL, 42.4 mmol). The tube was sealed and heated to 60 °C for 24 h. After that, the reaction solution was cooled to room temperature. An aqueous solution of $NaNO_2$ (465 mg in 5.0 mL H_2O) was added to the mixture, followed by dropwise addition of HCl (1 N), until the pH reached 2~3 and gases stopped evolving, at which point the mixture turned bright red. The product was extracted with EtOAc (3 × 50 mL), and the combined organic layers were washed with H_2O (2 × 20 mL) and brine (10 mL), dried over Na_2SO_4, filtered, and concentrated upon rotary evaporation. The crude product was purified by silica gel chromatography (PE : EA = 5 : 1) to yield **F-Tz1** as a red solid (25.0 mg, 10.0%).

Rf = 0.50 (PE : EA = 5 : 1)

M. P.: 168-170 °C

IR (KBr): 3021, 2991, 2934, 2845, 2823, 1617, 1491 cm^{-1}

^{1}H NMR (500 MHz, $CDCl_3$): δ 9.05-8.94 (m, 1H), 8.49 (dd, J = 8.6, 1.8 Hz, 1H), 7.86 (d, J = 9.1 Hz, 1H), 7.80 (d, J = 8.7 Hz, 1H), 7.34 (dd, J = 9.3, 2.4 Hz, 1H), 7.13 (s, 1H), 3.41-3.33 (m, 4H), 3.07 (s, 3H), 1.82-1.72 (m, 4H), 1.69-1.62 (m, 2H)

^{13}C NMR (126 MHz, $CDCl_3$): δ 166.70, 164.54, 151.59, 137.36, 130.43, 128.75, 127.64, 125.79, 124.27, 120.03, 109.18, 50.14, 25.77, 24.48, 21.25

ESI-HRMS (*m/z*): $[M+H]^+$ calc'd. for $C_{18}H_{20}N_5$ 306.1719, found 306.1719

Synthesis of F-Tz2. To a high pressure reaction tube was added **S4** (250 mg, 0.680 mmol), MeCN (360 µL, 6.84 mmol), $Zn(OTf)_2$ (124 mg, 0.270 mmol) and hydrazine hydrate (80%, 1.70 mL, 34.2 mmol). The tube was sealed and heated to 60 °C for 24 h. After cooled to room temperature, $NaNO_2$ (318 mg, 4.79 mmol) in H_2O (5.0 mL) was added to the mixture, followed by dropwise addition of HCl (1.0 N) until the pH reached 2~3 and gases stopped evolving, at which point the mixture had turned bright red. The product was extracted with EtOAc (3 × 50 mL), and the combined organic layers were washed with H_2O (2 × 20 mL) and brine (10 mL), dried over Na_2SO_4, filtered, and concentrated upon rotary evaporation. The crude product was purified by silica gel chromatography (DCM : MeOH = 100 : 1) to yield F-Tz2 as a red solid (55.0 mg, 18.5%).

Rf = 0.10 (PE : EA = 5 : 1)

M. P.: 246-247 °C

IR (KBr): 3355, 3302, 3030, 2969, 2939, 2876, 2857, 1705, 1619, 1531 cm^{-1}

^{1}**H NMR** (500 MHz, $CDCl_3$): δ 8.95 (d, J = 1.7 Hz, 1H), 8.47 (dd, J = 8.7, 1.8 Hz, 1H), 7.77 (d, J = 8.8 Hz, 1H), 7.72 (d, J = 8.7 Hz, 1H), 6.89 (dd, J = 8.8, 2.3 Hz, 1H), 6.81 (s, 1H), 4.45 (s, 1H), 3.97 (s, 1H), 3.51 (s, 1H), 3.46 – 3.34 (m, 1H), 3.06 (s, 3H), 2.30 – 2.20 (m, 2H), 2.16 – 2.09 (m, 2H), 1.46 (s, 9H), 1.37 – 1.30 (m, 4H)

^{13}**C NMR** (126 MHz, $CDCl_3$): δ 166.62, 164.55, 155.36, 143.67, 137.81, 131.01, 130.30, 129.06, 126.86, 124.54, 119.04, 109.78, 79.50, 49.35, 37.77, 32.26, 31.86, 28.57, 21.25

ESI-HRMS (*m/z*): $[M+H]^+$ calc'd. for $C_{24}H_{31}N_6O_2$ 435.2508, found 435.2502

Synthesis of probes F-Tz3 and F-Tz4 (Supplementary Figure S65)

Synthesis of S5. To a round-bottomed flask with a stir bar, 6-bromo-2-chloroquinoline (2.00 g, 8.25 mmol), NaI (3.90 g, 24.8 mmol), *p*-toluenesulfonic acid (0.280 g, 1.65 mmol), and acetone (30.0 mL) were added. The reaction mixture was heated to reflux and stirred overnight, then cooled to room temperature. Water (100 mL) was added to the flask and the reaction mixture was extracted with ethyl acetate (3 × 50 mL). The organic layers were combined, dried with Na_2SO_4, and filtered and the solvent was then removed by rotary evaporation to give a yellow solid (2.37 g, 86.0%) which was used for the next step directly without purification.

Rf = 0.70 (PE : EA = 5 : 1)

ESI-MS (*m/z*): $[M+H]^+$ calc'd. for C_9H_6BrIN 333.87, found 333.90, 335.90

Synthesis of S6[8]. To a round-bottomed flask with a stir bar, **S5** (2.37 g, 7.12 mmol), CuCN (0.77 g, 8.54 mmol), and pyridine (20.0 mL) were added. The reaction mixture was heated to 80 °C under a N_2 atmosphere. Upon consumption of **S5** as evidenced by TLC analysis, the reaction was cooled to room temperature. 1M HCl was added to the flask and the reaction mixture was extracted with ethyl acetate (3 × 50 mL), and the combined organic layers were washed with H_2O (2 × 20 mL) and brine (10 mL), dried over Na_2SO_4, filtered, and concentrated upon rotary evaporation. The crude product was purified by silica gel chromatography (PE : EA = 2 : 1) to yield **S6** as a faint yellow solid (740 mg, 44.6%).

Rf = 0.50 (PE : EA = 5 : 1)

^{1}**H NMR** (500 MHz, $CDCl_3/CD_3OD$ = 10/1) δ 8.21 (d, J = 8.5 Hz, 1H), 8.04 (s, 1H), 7.98 (d, J = 9.0 Hz, 1H), 7.86 (dd, J = 9.2, 2.4 Hz, 1H), 7.69 (d, J = 8.2 Hz, 1H)

^{13}**C NMR** (126 MHz, $CDCl_3/CD_3OD$ = 10/1) δ 146.71, 136.78, 135.10, 133.76, 131.40, 129.94, 129.70, 124.27, 124.14, 117.22.

ESI-MS (*m/z*): $[M+H]^+$ calc'd. for $C_{10}H_6BrN_2^+$ 232.97, found 233.10, 235.10

Synthesis of S7. A mixture of **S6** (267 mg, 1.15 mmol), piperidine (195 mg, 2.30 mmol), $Pd_2(dba)_3$ (63.0 mg, 0.07 mmol), BINAP (86.0 mg, 0.14 mmol) and Cs_2CO_3 (0.930 g, 2.90 mmol) was dissolved in 15.0 mL dry toluene. The reaction solution was stirred under reflux overnight under a N_2 atmosphere. Upon consumption of **S6** as evidenced by TLC analysis, the solvent was evaporated and the crude product was purified by silica gel chromatography (PE : EA = 10 : 1) to yield a yellow solid (160 mg, 59.0%).

Rf = 0.53 (PE : EA = 5 : 1)

M. P.: 109-111 °C

IR (KBr): 3003, 2928, 2851, 2224, 1610, 1505 cm^{-1}

^{1}H NMR (500 MHz, $CDCl_3$): *δ* 7.98 (d, *J* = 8.5 Hz, 1H), 7.93 (d, *J* = 9.4 Hz, 1H), 7.56 (dd, *J* = 9.5, 2.8 Hz, 1H), 7.52 (d, *J* = 8.4 Hz, 1H), 6.93 (d, *J* = 2.8 Hz, 1H), 3.48 – 3.31 (m, 4H), 1.77 – 1.70 (m, 4H), 1.70 – 1.64 (m, 2H)

^{13}C NMR (126 MHz, $CDCl_3$): *δ* 151.47, 143.37, 134.76, 130.86, 130.64, 128.99, 123.86, 123.79, 118.48, 106.84, 49.51, 25.58, 24.37

ESI-HRMS (*m/z*): $[M+H]^+$ calc'd. for $C_{15}H_{16}N_3$ 238.1344, found 288.1357

Synthesis of S8. A mixture of **S6** (560 mg, 2.40 mmol), *trans*-*N*-Boc-1,4-cyclohexanediamine (771 mg, 3.60 mmol), $Pd_2(dba)_3$ (137 mg, 0.150 mmol), BINAP (180 mg, 0.290 mmol) and Cs_2CO_3 (2.30 g, 7.20 mmol) was dissolved in 15.0 mL dry toluene, and the reaction solution was stirred under reflux overnight under N_2 atmosphere. Upon consumption of **S6** as evidenced by TLC analysis, the solvent was evaporated and the crude product was purified by silica gel chromatography (PE : EA = 5 : 1 – 2 : 1) to yield a yellow solid (0.630 g, 71.5%).

Rf = 0.27 (PE : EA = 2 : 1)

M. P.: 182-185 °C

IR (KBr): 3359, 3024, 2983, 2934, 2900, 2854, 2231, 1681, 1621, 1544 cm^{-1}

^{1}H NMR (500 MHz, $CDCl_3$): *δ* 7.88 (d, *J* = 8.5 Hz, 1H), 7.80 (d, *J* = 9.1 Hz, 1H), 7.45 (d, *J* = 8.5 Hz, 1H), 7.09 (dd, *J* = 9.2, 2.6 Hz, 1H), 6.58 (d, *J* = 2.5 Hz, 1H), 4.62 – 4.50 (m, 1H), 4.41 (brs, 1H), 3.47 (s, 1H), 3.40 – 3.28 (m, 1H), 2.25 – 2.14 (m, 2H), 2.13 – 2.05 (m, 2H), 1.42 (s, 9H), 1.34 – 1.26 (m, 4H)

^{13}C NMR (126 MHz, $CDCl_3$): *δ* 155.34, 147.42, 143.05, 133.86, 131.43, 130.92, 127.61, 123.90, 123.53, 118.52, 101.59, 79.40, 51.12, 49.22, 32.02, 31.52, 28.47

ESI-HRMS (*m/z*): $[M+H]^+$ calc'd. for $C_{21}H_{27}N_4O_2$ 367.2134, found 367.2123

Synthesis of F-Tz3. To a high pressure reaction tube was added **S7** (0.100 g, 0.420 mmol), MeCN (221 μL, 4.20 mmol), $Ni(OTf)_2$ (43.8 mg, 0.210 mmol) and hydrazine hydrate (80%, 1.0 mL, 21.0 mmol). The tube was sealed and heated to 70 °C for 24 h. Upon consumption of **S7** as evidenced by TLC analysis, the solvent was evaporated and the crude product was purified by silica gel chromatography (PE : EA = 5 : 1). The resulting yellow solid was further dissolved in 10 mL methanol and the air was continuously pumped into the solution to oxidize the intermediate into tetrazine. Then the solvent was evaporated and the crude product was purified by recrystallization (dichloromethane/petroleum ether) as a red solid (25.0 mg, 19.4%).

Rf = 0.37 (PE : EA = 2 : 1)

M. P.: 195-197 °C

IR (KBr): 3031, 2934, 2845, 2832, 1613, 1498 cm^{-1}

^{1}H NMR (500 MHz, $CDCl_3$): *δ* 8.58 (d, *J* = 8.6 Hz, 1H), 8.23 (d, *J* = 9.4 Hz, 1H), 8.18 (d, *J* = 8.6 Hz, 1H), 7.60 (d, *J* = 9.4 Hz, 1H), 7.06 (d, *J* = 7.9 Hz, 1H), 3.41 (t, *J* = 5.5 Hz, 4H), 3.15 (s, 3H), 1.78 (s, 4H), 1.71 – 1.63 (m, 2H)

^{13}C NMR (126 MHz, $CDCl_3$): *δ* 167.71, 164.08, 151.13, 146.37, 143.88, 135.53, 131.62, 131.06, 123.36, 120.68, 107.51, 49.90, 25.71, 24.42, 21.48

ESI-HRMS (*m/z*): $[M+H]^+$ calc'd. for $C_{17}H_{19}N_6$ 307.1671, found 306.1672

Synthesis of F-Tz4. To a high pressure reaction tube was added **S8** (0.100 g, 0.270 mmol), MeCN (152 μL, 2.70 mmol), $Ni(OTf)_2$ (28.0 mg, 0.140 mmol) and hydrazine hydrate (80%, 680 μL, 13.5 mmol).

The tube was sealed and heated to 70 °C for 24 h. Upon consumption of **S8** as evidenced by TLC analysis, the solvent was evaporated and the crude product was purified by silica gel chromatography (PE : EA = 5 : 1 – 2 : 1). The resulted intermediate was then dissolved in 10 mL methanol and the air was continuously pumped into the solution to oxidize the intermediate. Upon completion, the solvent was evaporated and the crude product was purified by recrystallization (dichloromethane/petroleum ether) as a red solid (35.0 mg, 27.6%).

Rf = 0.33 (DCM : MeOH = 20 : 1)

M. P.: 236-238 °C

IR (KBr): 3329, 3028, 2970, 2930, 2853, 1689, 1624, 1531, 1498, 1174 cm^{-1}

^{1}H NMR (500 MHz, $CDCl_3$): δ 8.55 (d, J = 8.6 Hz, 1H), 8.14 (d, J = 9.1 Hz, 1H), 8.10 (d, J = 8.6 Hz, 1H), 7.16-7.10 (m, 1H), 6.74 (s, 1H), 4.46 (s, 1H), 4.18 (s, 1H), 3.52 (s, 1H), 3.45-3.36 (m, 1H), 3.14 (s, 3H), 2.30-2.20 (m, 2H), 2.17-2.10 (m, 2H), 1.46 (s, 9H), 1.38-1.30 (m, 4H)

^{13}C NMR (126 MHz, $CDCl_3$): δ 167.61, 164.02, 155.35, 146.64, 145.32, 143.53, 134.71, 132.13, 131.62, 122.76, 120.84, 102.69, 79.52, 51.51, 49.32, 32.21, 31.76, 28.56, 21.44

ESI-HRMS (*m/z*): $[M+H]^+$ calc'd. for $C_{23}H_{30}N_7O_2$ 436.2461, found 436.2461

Synthesis of oxadiazole derivatives of F-Tz1-F-Tz4 (P1-P4) (Supplementary Figure S66)

To a round-bottom flask with a stir bar, the corresponding probe (3.0 mg) was dissolved in 10.0 ml acetonitrile. KO_2 powder was dissolved in dry MeCN containing 1% 18-crown-6, and the solution was filtrated by filter membrane. The clear and saturated KO_2 solution (20 – 30 mL) was added slowly at a rate of 5.0 mL/h to the probe solution while the mixture was stirred at room temperature. Upon consumption of the probe as evidenced by TLC analysis, the reactions were quenched with water. Then the volatile part was removed by rotary evaporation. The product was extracted with EtOAc (3 × 10 mL), and the combined organic layers were washed with H_2O (2 × 10 mL) and brine (10 mL), dried over Na_2SO_4, filtered, and concentrated upon rotary evaporation.

P1 was purified by silica gel chromatography (PE : EA = 5 : 1) as a creamy white solid.

Rf = 0.50 (PE : EA = 5 : 1)

ESI-HRMS (*m/z*): $[M+H]^+$ calc'd. for $C_{17}H_{17}N_3O$ 294.1606; found: 294.1611

P2 was purified by silica gel chromatography (PE : EA = 2 : 1) as a yellow solid.

Rf = 0.65 (PE : EA = 2 : 1)

ESI-HRMS (*m/z*): $[M+H]^+$ calc'd. for $C_{24}H_{31}N_4O_3$ 423.2396; found: 423.2394

P3 was purified by silica gel chromatography (DCM : MeOH = 20 : 1) as a creamy white solid.

Rf = 0.49 (DCM : MeOH = 20 : 1)

ESI-HRMS (*m/z*): $[M+H]^+$ calc'd. for $C_{17}H_{19}N_4O$ 295.1559; found: 295.1554

P4 was purified by silica gel chromatography (DCM : MeOH = 20 : 1) as a creamy white solid.

Rf = 0.58 (DCM : MeOH = 20 : 1)

ESI-HRMS (*m/z*): $[M+H]^+$ calc'd. for $C_{23}H_{30}N_5O_3$ 424.2349; found: 424.2349

Synthesis of probe F-Tz5 (Supplementary Figure S67)

To a 45 mL reaction tube equipped with a stir bar, nitrile substrate (1.0 eq), catalyst (3-mercaptopropionic acid, 0.20 eq), and hydrazine hydrate (80%, 10 eq) were added. The reaction was stirred at 40 °C. Upon completion, the reaction solution was cooled with ice water and ethyl acetate (50 mL). The organic phase was washed twice with 50 mL water, once with 50 mL brine and dried over Na_2SO_4. The solvent was evaporated and the crude product was purified by silica gel chromatography to yield the dihydrotetrazine intermediate S9. **ESI-MS** (*m/z*): $[M+H]^+$ calc'd. for $C_{14}H_{13}N_4O_2$ 269.10, found 269.30.Transfer S9 to a round-bottomed flask. Dichloromethane (20 mL) was added, and the suspended mixture was stirred. To the stirring suspension was added phenyliodonium diacetate (1.5 eq). The reaction mixture was allowed to stir until all ingredients were oxidized as shown by TLC analysis. And then the crude product was purified by silica gel chromatography (PE : EA = 5 : 1 – PE : EA = 2 : 1) to yield the tetrazine as a pink solid.

F-Tz5

Yield: 13.4%

Rf = 0.70 (PE : EA = 2 : 1)

M. P.: 246-247 °C

IR (KBr): 3117, 2106, 3070, 3055,1615, 1576 cm^{-1}

^{1}**H NMR** (500 MHz, DMSO- *d6*) *δ* 10.72 (s, 2H), 8.27 (dd, *J* = 8.2, 1.8 Hz, 2H), 7.72 – 7.45 (m, 2H), 7.27 – 6.94 (m, 4H)

^{13}**C NMR** (126 MHz, DMSO- *d6*) *δ* 164.16, 158.54, 134.62, 130.04, 120.50, 118.38, 117.28

ESI-MS (*m/z*): $[M+H]^+$ calc'd. for $C_{14}H_{11}N_4O_2$ 267.09, found 267.25

Synthesis of probe F-Tz6 (Supplementary Figure S68)

Synthesis of S11[9]. To a round-bottomed flask with a stir bar, **S10[10]** (0.900 g, 3.15 mmol), N-Hydroxysuccinimde (0.440 g, 3.78 mmol) and DMF (5.0 mL) were added. EDCI was dissolved in DMF and slowly added to the reaction solution, and the reaction mixture was stirred overnight at room temperature. Water (20 mL) was added to the flask and filtered. And the filtered solid was purified by silica gel chromatography (DCM : MeOH = 50 : 1) to yield a yellow solid (0.850 g, 66.3%).

Rf = 0.30 (DCM : MeOH = 20 : 1)

^{1}**H NMR** (500 MHz, DMSO-d_6) *δ* 8.54 (s, 1H), 7.23 (s, 1H), 3.39-3.36 (m, 4H), 2.85 (m, 4H), 2.72 – 2.62 (m, 4H), 1.93 – 1.79 (m, 4H).

^{13}**C NMR** (126 MHz, DMSO-d_6) *δ* 170.76, 159.47, 156.77, 153.70, 150.56, 150.24, 128.32, 120.05, 107.29, 104.72, 98.57, 50.04, 49.49, 26.79, 25.58, 20.44, 19.46, 19.43.

ESI-MS (*m/z*): $[M+H]^+$ calc'd. for $C_{20}H_{19}N_2O_6$ 383.12, found 383.05

Synthesis of S13. To a round-bottomed flask with a stir bar, **S12** (0.800 g, 2.65 mmol), and HCl/EA (20.0 mL) were added. The reaction mixture was stirred overnight at room temperature. Upon consumption of **S12** as evidenced by TLC analysis, the solvent was then removed by rotary evaporation to give a red solid **S13** which was used for the next step directly without purification.

Rf = 0.10 (DCM : MeOH = 20 : 1)

ESI-MS (*m/z*): $[M+H]^+$ calc'd. for $C_{10}H_{12}N_5$ 202.11, found 202.30

Synthesis of F-Tz6. To a round-bottomed flask with a stir bar, **S11** (0.120 g, 0.310 mmol), **S13** (81.8 mg, 0.350 mmol), DIPEA (114 μL, 0.690 mmol), and methanol (10.0 mL) were added. The reaction

mixture was stirred overnight under a N_2 atmosphere. Upon consumption of **S11** as evidenced by TLC analysis, the reaction mixture was filtered. And the filtered solid was purified by silica gel chromatography (DCM : MeOH = 100 : 1 – 10 : 1) to yield an orange solid (0.100 g, 68.0%).

Rf = 0.67 (DCM : MeOH = 20 : 1)

M. P.: 240-243 °C

IR (KBr): 3316, 3031, 2934, 2895, 2854, 1690, 1611, 1581, 1533 cm^{-1}

^{1}H NMR (500 MHz, $CDCl_3$) δ 9.37 (t, *J* = 6.0 Hz, 1H), 8.62 (s, 1H), 8.58 – 8.49 (m, 2H), 7.56 (d, *J* = 8.4 Hz, 2H), 6.99 (s, 1H), 4.75 (d, *J* = 6.1 Hz, 2H), 3.32 (q, *J* = 5.2 Hz, 4H), 3.07 (s, 3H), 2.87 (t, *J* = 6.4 Hz, 2H), 2.75 (t, *J* = 6.3 Hz, 2H), 1.96 (hept, *J* = 6.0, 5.1 Hz, 4H)

^{13}C NMR (126 MHz, $CDCl_3$) δ 167.21, 164.04, 163.25, 152.82, 148.49, 148.40, 143.94, 130.63, 128.35, 128.28, 128.25, 127.19, 119.82, 108.56, 108.33, 105.72, 50.34, 49.92, 43.30, 27.54, 21.25, 21.18, 20.24, 20.18

ESI-HRMS (*m/z*): $[M+H]^+$ calc'd. for $C_{26}H_{25}N_6O_3$ 469.1988, found 469.1977

Synthesis of probe F-Tz7 (Supplementary Figure S69)

Synthesis of F-Tz7. A mixture of **S14**[11] (80.0 mg, 0.200 mmol), **S13** (50.0 mg, 0.240 mmol), $Pd_2(dba)_3$ (1.8 mg, 0.002 mmol), Xantphos (1.1 mg, 0.002 mmol) and Cs_2CO_3 (195 mg, 0.600 mmol) was dissolved in 5.0 mL dry toluene. The reaction solution was stirred under reflux overnight under N_2 atmosphere. Upon consumption of **S14** as evidenced by TLC analysis, the solvent was evaporated and the crude product was purified by silica gel chromatography (DCM : MeOH = 100 : 1 – 30 : 1) to yield an orange solid (25.0 mg, 10.0%).

S14

Rf = 0.30 (PE : EA = 1 : 1)

^{1}H NMR (400 MHz, $CDCl_3$) δ 8.63 (dd, *J* = 7.3, 1.2 Hz, 1H), 8.55 (dd, *J* = 8.5, 1.1 Hz, 1H), 8.39 (d, *J* = 7.8 Hz, 1H), 8.03 (d, *J* = 7.8 Hz, 1H), 7.84 (dd, *J* = 8.5, 7.3 Hz, 1H), 4.33 (t, *J* = 6.9 Hz, 2H), 3.67 (t, *J* = 4.6 Hz, 4H), 2.69 (t, *J* = 4.6 Hz, 2H), 2.62 – 2.54 (m, 4H).

^{13}C NMR (101 MHz, $CDCl_3$) δ 163.75, 163.72, 133.44, 132.17, 131.36, 131.24, 130.75, 130.45, 129.14, 128.23, 123.16, 122.29, 67.18, 56.21, 53.95, 37.45.

F-Tz7

Rf = 0.27 (DCM : MeOH = 20 : 1)

M. P.: 207-209 °C

IR (KBr): 3386, 3377, 3324, 3306, 3053, 2956, 2921, 2896, 2856, 1681, 1644, 1585, 1550, 1244, 1115 cm^{-1}

^{1}H NMR (500 MHz, $CDCl_3$) δ 8.59 (dd, *J* = 7.8, 4.1, 2.0 Hz, 3H), 8.40 (d, *J* = 8.4 Hz, 1H), 8.25 – 8.17 (m, 1H), 7.69 – 7.57 (m, 3H), 6.72 (d, *J* = 8.4 Hz, 1H), 5.87 (t, *J* = 5.3 Hz, 1H), 4.76 (d, *J* = 5.4 Hz, 2H), 4.32 (t, *J* = 7.1 Hz, 2H), 3.69 (q, *J* = 4.6 Hz, 4H), 3.10 (s, 3H), 2.70 (t, *J* = 7.1 Hz, 2H), 2.61 (s, 4H)

^{13}C NMR (126 MHz, $CDCl_3$) δ 167.51, 164.69, 164.11, 163.82, 149.00, 142.10, 134.35, 132.91, 131.61, 131.36, 129.85, 128.65, 128.21, 127.84, 126.16, 125.19, 123.27, 120.53, 111.30, 105.25, 67.14, 56.36, 53.91, 47.72, 37.10, 21.32

ESI-HRMS (*m/z*): $[M+H]^+$ calc'd. for $C_{28}H_{28}N_7O_3$ 510.2254, found 510.2253

Synthesis of probe F-Tz8 (Supplementary Figure S70)

Synthesis of S15[12]. To a round-bottomed flask with a stir bar, 4-(Chloromethyl)benzonitrile (3.00 g, 19.8 mmol), and Monomethylamine ethanol solution (15.0 mL) were added. The reaction mixture was stirred overnight at room temperature. Upon consumption of 4-(Chloromethyl)benzonitrile as evidenced by TLC analysis, the solvent was removed by rotary evaporation. The crude product was purified by silica gel chromatography (PE : EA = 2 : 1 – DCM : MeOH = 10 : 1) to yield **S15** as a faint yellow oil (1.74 g, 60.0%).

Rf = 0.27 (PE : EA = 2 : 1)

^{1}H NMR (500 MHz, $CDCl_3$) δ 7.62 – 7.56 (m, 2H), 7.45 – 7.38 (m, 2H), 3.79 (s, 2H), 2.42 (s, 3H).

^{13}C NMR (126 MHz, $CDCl_3$) *δ* 145.84, 132.28, 128.77, 119.04, 110.79, 55.53, 36.10.

ESI-MS (*m/z*): $[M+H]^+$ calc'd. for $C_9H_{11}N_2$ 147.09, found 147.29

Synthesis of S16[5]. To a high pressure reaction tube was added **S15** (1.67 g, 11.5 mmol), MeCN (3.00 mL, 57.2 mmol), $Zn(OTf)_2$ (2.00 g, 5.74 mmol) and hydrazine hydrate (80%, 28.0 mL, 574 mmol). The tube was sealed and heated to 60 °C for 24 h. After that the reaction solution was cooled to room temperature. An aqueous solution of $NaNO_2$ (465 mg in 5.0 mL H_2O) was added to the mixture, followed by dropwise addition of HCl (1 N), until the pH reached 2 ~ 3 and gases stopped evolving, at which point the mixture turned bright red. The product was extracted with EtOAc (3 × 50 mL), and the combined organic layers were washed with H_2O (2 × 20 mL) and brine (10 mL), dried over Na_2SO_4, filtered, and concentrated upon rotary evaporation. The crude product was purified by silica gel chromatography (DCM : MeOH = 10 : 1) to yield **S16** as a red solid (0.500 g, 20.4%).

Rf = 0.17 (DCM : MeOH = 20 : 1)

M. P.: 74 -75 °C

^{1}H NMR (400 MHz, DMSO-*d6*) *δ* 8.55 – 8.48 (m, 2H), 7.77 – 7.68 (m, 2H), 4.19 (s, 2H), 3.01 (s, 3H), 2.57 (s, 3H).

^{13}C NMR (101 MHz, DMSO-*d6*) *δ* 167.32, 163.05, 137.87, 132.12, 130.48, 127.69, 51.60, 32.96, 20.91.

ESI-MS (*m/z*): $[M+H]^+$ calc'd. for $C_{11}H_{14}N_5$ 216.12, found 216.25

Synthesis of F-Tz8. A mixture of **S17** (445 mg, 0.930 mmol), **S16** (220 mg, 1.02 mmol), EDCI (444 mg, 2.30 mmol) and HOBT (188 mg, 1.39 mmol) was dissolved in 10 mL dry DCM. Then added DIPEA (615 μL, 3.72 mmol) at 0 °C, and the reaction solution was stirred overnight under a N_2 atmosphere. Upon consumption of **S17** as evidenced by TLC analysis, water (20.0 mL) was added to the flask and the reaction mixture was extracted with ethyl acetate (3 × 50 mL). The organic layers were combined, dried with Na_2SO_4, filtered and the solvent was then removed by rotary evaporation. And the crude product was purified by silica gel chromatography (DCM : MeOH = 30 : 1) to yield a crimson solid (0.100 g, 16.8%).

Rf = 0.16 (DCM : MeOH = 20 : 1)

M. P.: 106 -108 °C

IR (KBr): 3473, 3077, 2975, 2926, 2872, 2853, 1632, 1588, 1528 cm^{-1}

^{1}H NMR (500 MHz, CD_3OD) *δ* 8.22 (d, *J* = 8.0 Hz, 2H), 7.78 (tdd, *J* = 8.9, 3.5, 1.9 Hz, 3H), 7.54 – 7.49 (m, 1H), 7.26 (d, *J* = 9.5 Hz, 2H), 7.00 (dd, *J* = 9.5, 2.5 Hz, 2H), 6.95 (d, *J* = 2.5 Hz, 2H), 6.89 (d, *J* = 8.0 Hz, 2H), 4.57 (s, 2H), 3.64 (ddq, *J* = 29.3, 14.5, 7.2 Hz, 8H), 3.09 (s, 3H), 2.93 (s, 3H), 1.27 (q, *J* = 7.5, 6.5 Hz, 12H)

^{13}C NMR (126 MHz, CD_3OD) δ 169.25, 167.66, 163.54, 157.77, 155.83, 155.52, 141.11, 135.64, 131.84, 131.00, 130.86, 130.12, 129.83, 129.68, 128.14, 127.34, 127.20, 113.94, 113.54, 95.96, 49.72, 45.54, 36.43, 19.76, 11.40

ESI-HRMS (*m/z*): $[M]^+$ calc'd. for $C_{39}H_{42}N_7O_2^+$ 640.3395, found 640.3409

Synthesis of probe F-Tz9 (Supplementary Figure S71)

F-Tz9 was prepared by literature procedures.[13]

S18[14]

Rf = 0.90 (PE : EA = 10 : 1)

^{1}H NMR (500 MHz, $CDCl_3$) δ 8.13 (d, J = 8.2 Hz, 1H), 7.81 (d, J = 1.7 Hz, 1H), 7.70 (dd, J = 8.2, 1.7 Hz, 1H).

S19[15]

Rf = 0.50 (PE : EA = 10 : 1)

^{1}H NMR (500 MHz, $CDCl_3$) δ 10.38 (d, J = 0.8 Hz, 1H), 8.00 (d, J = 8.0 Hz, 1H), 7.97 (d, J = 1.4 Hz, 1H), 7.73 (ddd, J = 8.0, 1.5, 0.8 Hz, 1H).

S20

Rf = 0.50 (DCM : MeOH = 20 : 1)

^{1}H NMR (500 MHz, $CDCl_3$) δ 8.52 (s, 1H), 8.38 (s, 1H), 7.93 (d, J = 8.5 Hz, 1H), 7.82 (d, J = 9.4 Hz, 1H), 7.44 (dd, J = 8.5, 1.5 Hz, 1H), 7.35 (dd, J = 9.4, 2.5 Hz, 1H), 7.09 (d, J = 2.5 Hz, 1H), 3.20 (s, 6H).

^{13}C NMR (126 MHz, $CDCl_3$) δ 152.24, 152.19, 147.81, 135.48, 134.76, 129.92, 129.22, 125.81, 122.95, 122.39, 119.44, 119.27, 113.06, 103.30, 40.46.

ESI-MS (*m/z*): $[M+H]^+$ calc'd. for $C_{16}H_{14}N_3$ 248.12, found 248.20

F-Tz9

Rf = 0.17 (DCM : MeOH = 20 : 1)

M. P.: 215 -218 °C

IR (KBr): 3031, 2956, 2921, 2851, 1631, 1598, 1493, 1404, 1362 cm^{-1}

^{1}H NMR (500 MHz, $CDCl_3$) δ 9.40 (dd, J = 1.7, 0.9 Hz, 1H), 8.58 (s, 1H), 8.49 (dd, J = 8.7, 1.7 Hz, 1H), 8.08 (d, J = 8.7 Hz, 1H), 7.84 (d, J = 9.3 Hz, 1H), 7.33 (dd, J = 9.3, 2.5 Hz, 1H), 7.17 (d, J = 2.4 Hz, 1H), 3.20 (s, 6H), 3.13 (s, 3H)

^{13}C NMR (126 MHz, $CDCl_3$) δ 167.31, 164.57, 152.09, 151.93, 149.27, 135.37, 133.05, 129.73, 129.55, 129.22, 126.51, 122.27, 121.07, 118.99, 103.93, 40.55, 21.36

ESI-HRMS (*m/z*): $[M+H]^+$ calc'd. for $C_{18}H_{17}N_6$ 317.1515; found: 317.1511

General liquid chromatography methods

Method A: The mobile phase A: 1‰ CF_3COOH in distilled water. The mobile phase B: 1‰ CF_3COOH in MeOH. Running time: 0-15 min: 5% phase A and 95% phase B. Flow rate: 1 mL/min. Detection wavelength: 280 or 325 nm. Column: Ultimate®XB-C18, 5 μm, 4.6 × 250 mm.

Method B: The mobile phase A: 1‰ CF_3COOH in distilled water. The mobile phase B: 1‰ CF_3COOH in MeOH. Running time: 0-3 min: 30% phase A and 70% phase B, 3-8 min: 30-5% phase A and 70-95% phase B, 8-10 min: 5% phase A and 95% phase B. 10-11 min: 5-30% phase A and

95-70% phase B, 11-15 min: 30% phase A and 70% phase B. Flow rate: 1 mL/min. Detection wavelength: 240, 254 or 280 nm. Column: Ultimate® XB-C18, 5 μm, 4.6 × 250 mm.

Method C: The mobile phase A: 1‰ CF_3COOH in distilled water. The mobile phase B: 1‰ CF_3COOH in MeOH. Running time: 0-3 min: 60% phase A and 40% phase B, 3-8 min: 60-5% phase A and 40-95% phase B, 8-10 min: 5% phase A and 95% phase B. 10-11 min: 5-60% phase A and 95-40% phase B, 11-15 min: 60% phase A and 40% phase B. Flow rate: 1 mL/min. Detection wavelength: 240 or 254 nm. Column: Ultimate® XB-C18, 5 μm,4.6×250 mm.

Method D: The mobile phase A: 1‰ CF_3COOH in distilled water. The mobile phase B: 1‰ CF_3COOH in MeOH. Running time: 0-3 min: 45% phase A and 55% phase B, 3-10 min: 45-5% phase A and 55-95% phase B, 10-11 min: 5% phase A and 95% phase B. 11-14 min: 5-45% phase A and 95-55% phase B, 14-15 min: 45% phase A and 55% phase B. Flow rate: 1 mL/min. Detection wavelength: 300 nm. Column: Ultimate® XB-C18, 5 μm, 4.6 × 250 mm.

Preparation of various analytes for the selectivity experiment

$O_2^{\bullet-}$ [16-18] : KO_2 powder was dissolved in dry MeCN by adding a suiSupplementary Table amount of 18-crown-6 to increase the solubility. The solution was filtered through a membrane filter. The concentration of generated $O_2^{\bullet-}$ could be calculated by using a UV-vis spectrophotometer with the extinction coefficient at 255 nm (ε= 1460 M^{-1} cm^{-1}).

H_2O_2 and NaClO: These solutions were prepared by diluting commercial H_2O_2 and NaClO solutions with PBS (10 mM, pH 7.4) to make 100 mM stock solutions.

***t*BHP**: *tert*-Butylhydroperoxide (*t*BHP) was dissolved in PBS (10 mM, pH 7.4) 1 h before use to make a stock solution of 100 mM.

$ONOO^-$: To a vigorously stirred solution of $NaNO_2$ (0.6 M, 10 mL) and H_2O_2 (0.7 M, 10 mL) in deionized H_2O at 0 °C was added HCl (0.6 M, 10 mL), immediately followed by the rapid addition of NaOH (1.5 M, 20 mL). Excess hydrogen peroxide was removed by passing the solution through a short column of MnO_2. The concentration of $ONOO^-$ was determined by UV analysis with the extinction coefficient at 302 nm (ε= 1670 M^{-1} cm^{-1}). Aliquots of the solution were stored at -20 °C for use.

NO: NO was administrated with DEA·NONOate as a donor. DEA·NONOate was prepared in 0.01 M NaOH to make a 10 mM stock solution.

·OH: It was generated by Fenton reaction. To a solution of H_2O_2 (100 mM, 1.0 mL) in PBS (10 mM, Ph 7.4) was added $FeSO_4$ solution (100 mM, 1.0 mL) at ambient temperature (stock solution 50 mM).

GSH, Cys, and Hcy: These solutions were prepared by dissolving commercial GSH, Cys, and Hcy powder with deionized water to make 100 mM stock solutions.

Supplementary spectra

HRMS spectra of S3

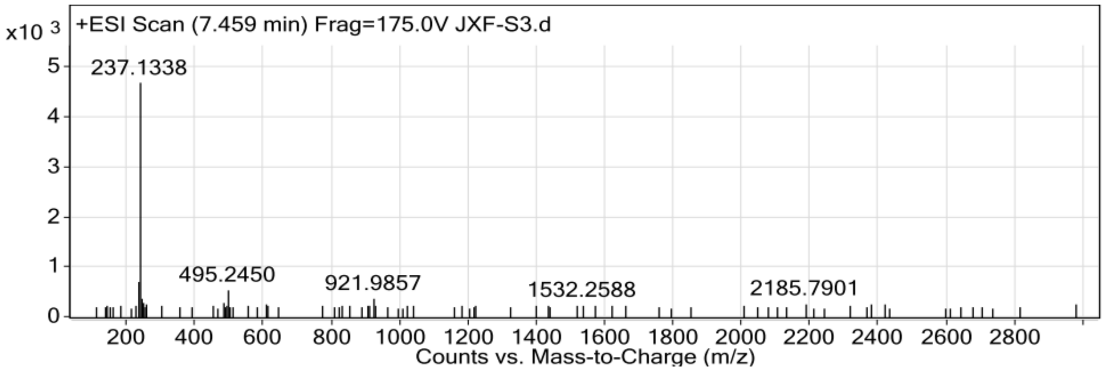

HRMS spectra of S4

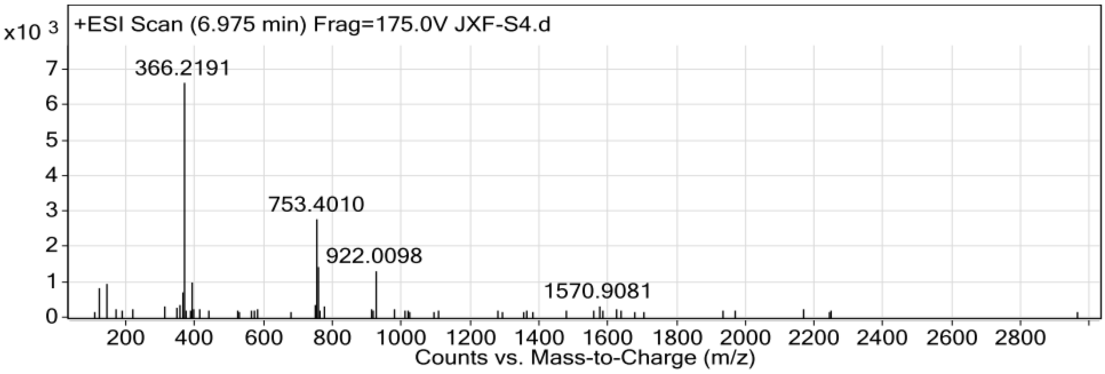

HRMS spectra of S7

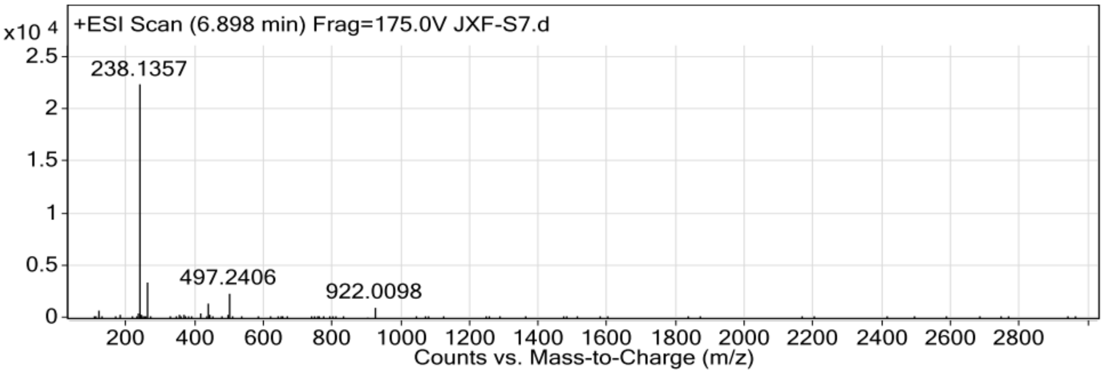

HRMS spectra of S8

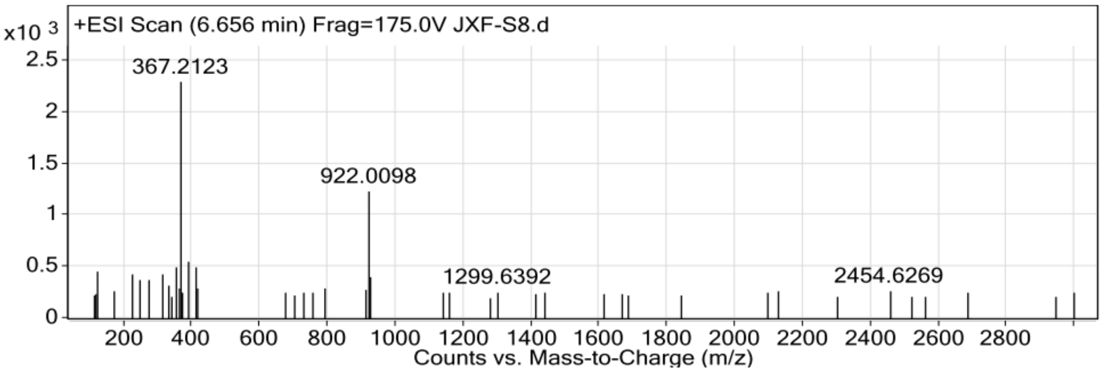

HRMS spectra of F-Tz1

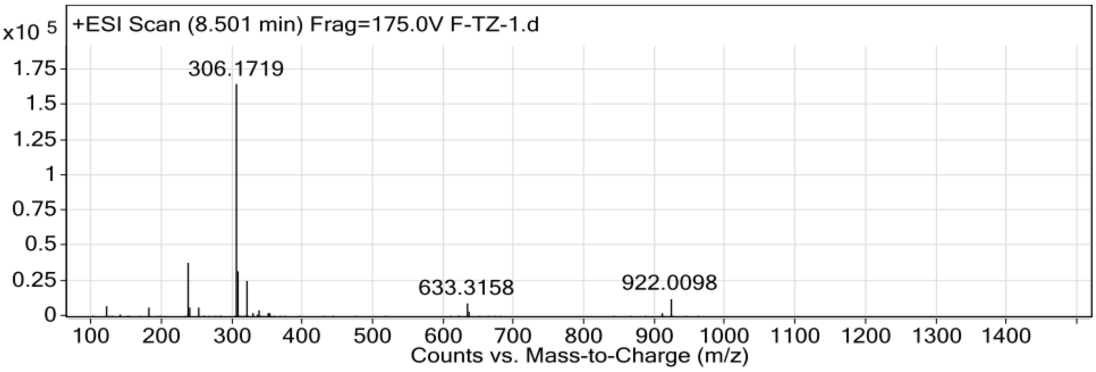

HRMS spectra of F-Tz2

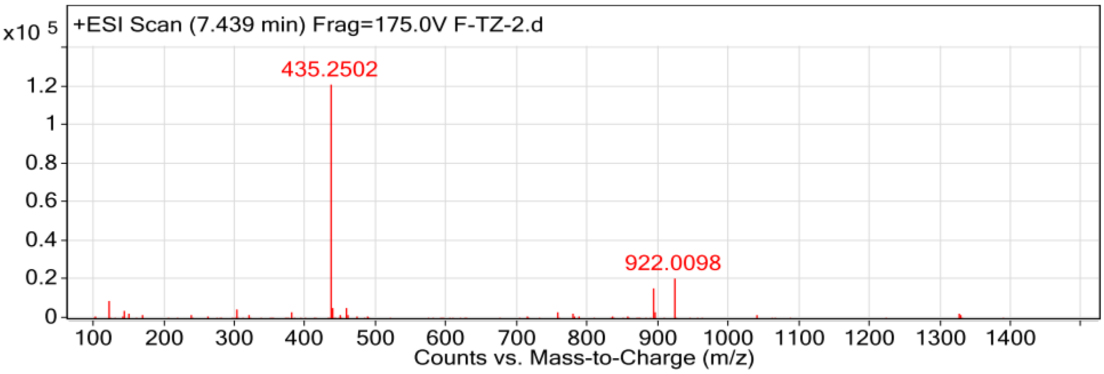

HRMS spectra of F-Tz3

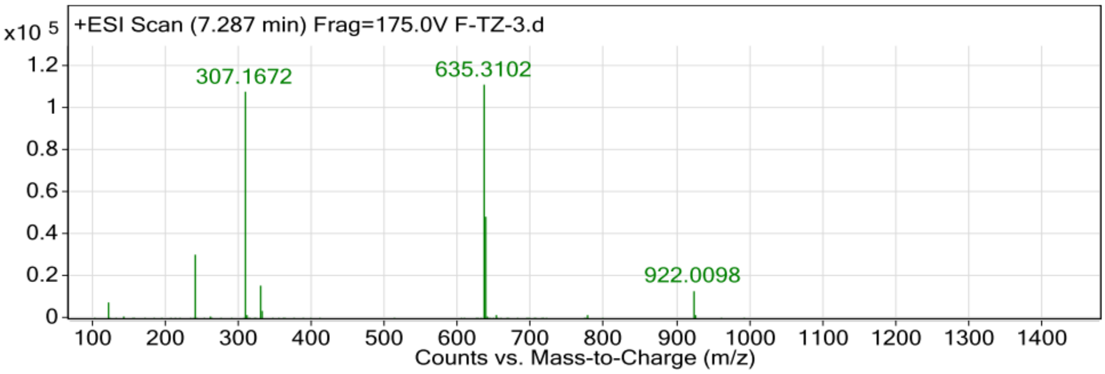

HRMS spectra of F-Tz4

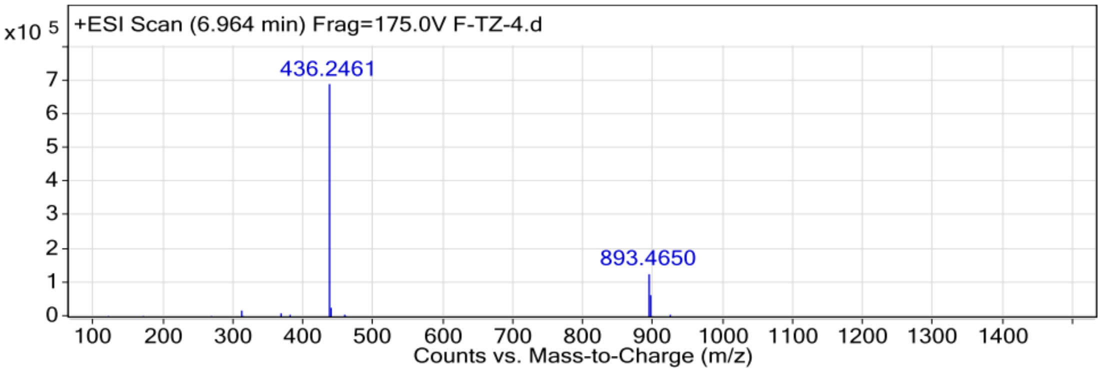

HRMS spectra of F-Tz6

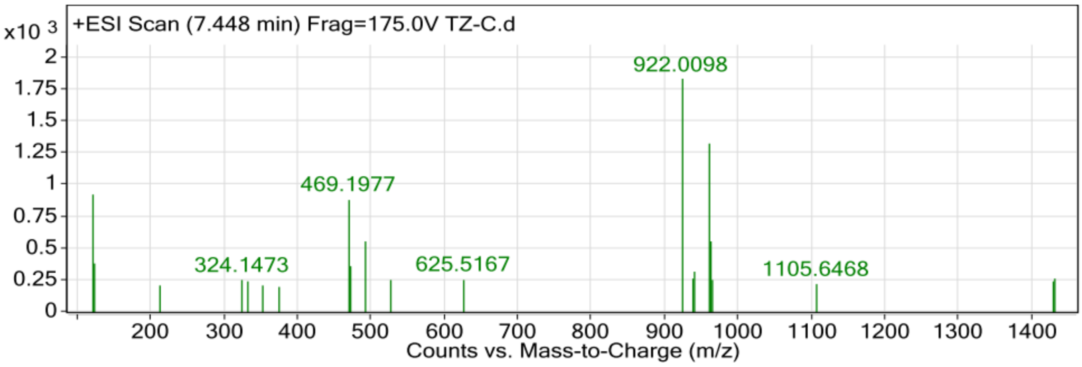

HRMS spectra of F-Tz7

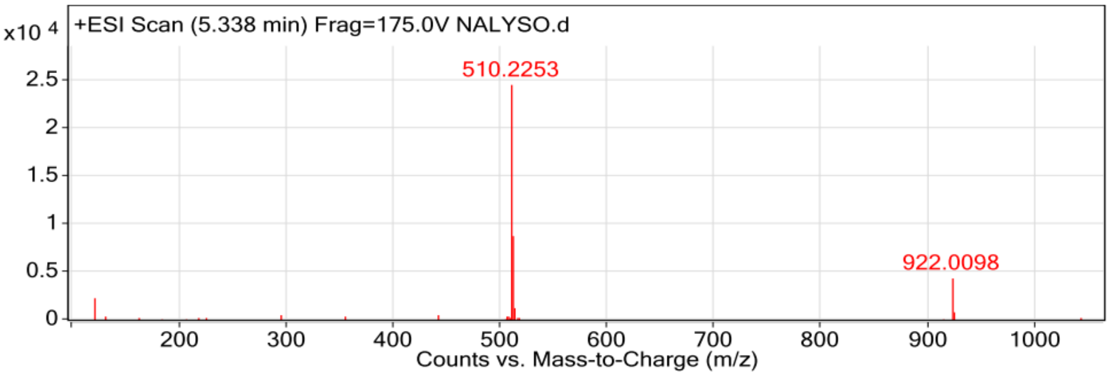

HRMS spectra of F-Tz8

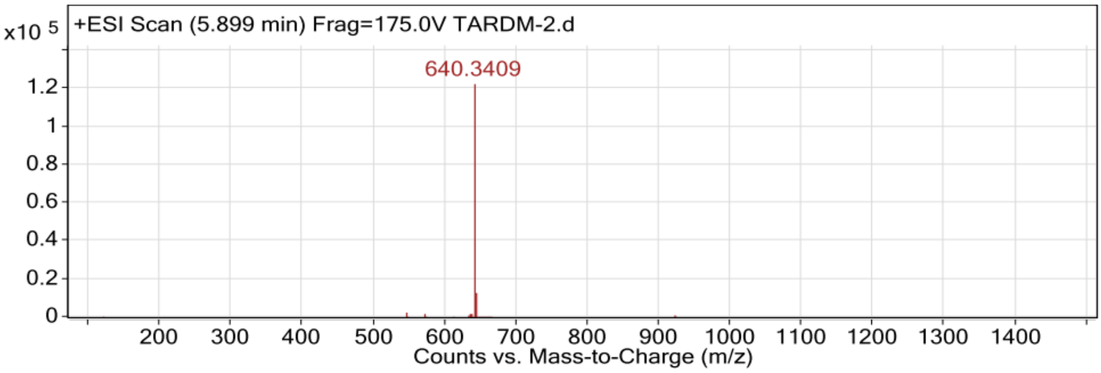

HRMS spectra of F-Tz9

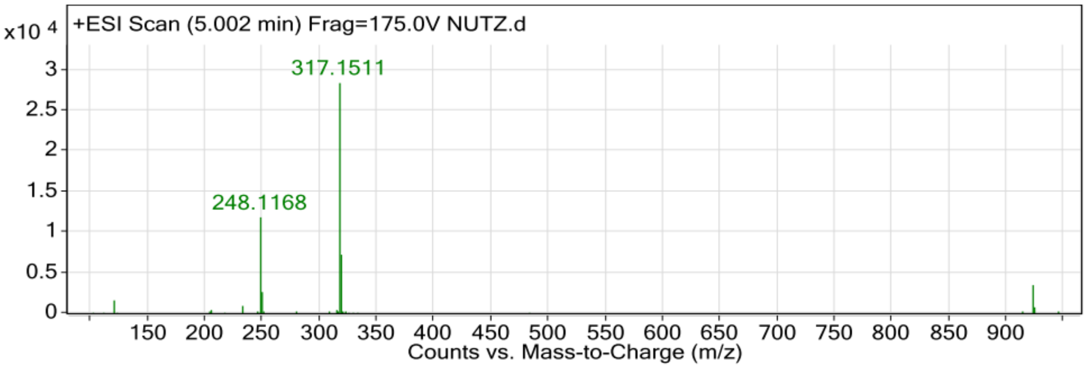

HRMS spectra of P1

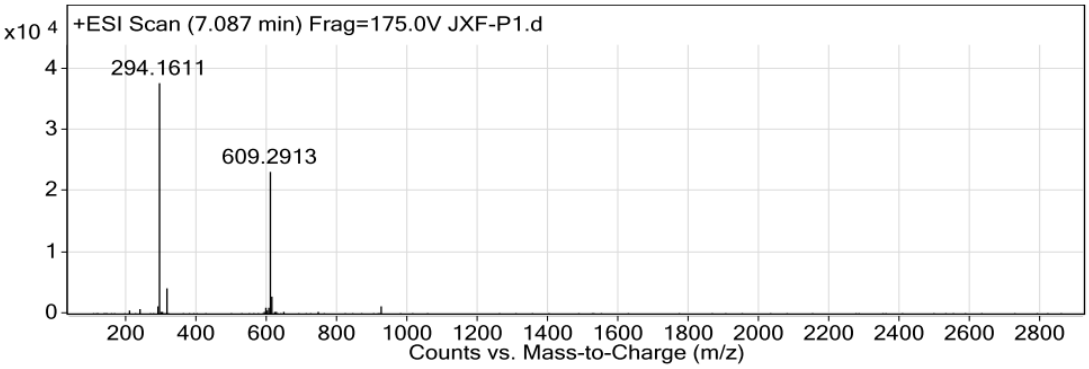

HRMS spectra of P2

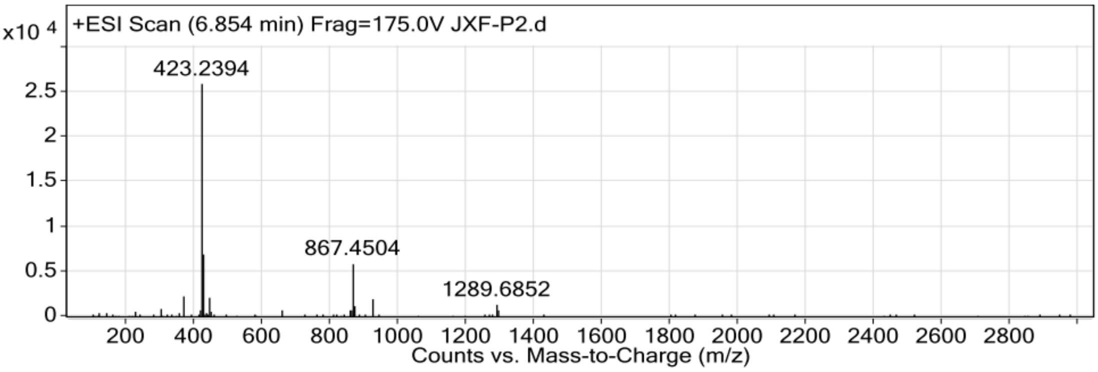

HRMS spectra of P3

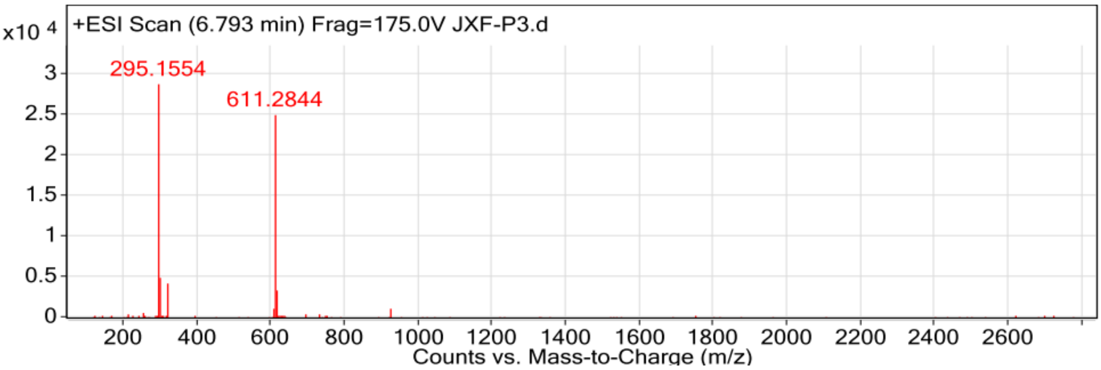

HRMS spectra of P4

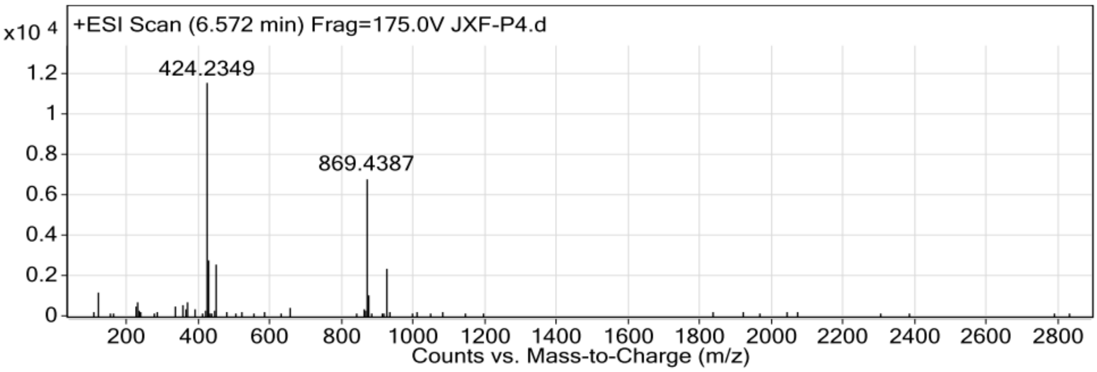

^{1}H NMR and ^{13}C NMR of Tz1

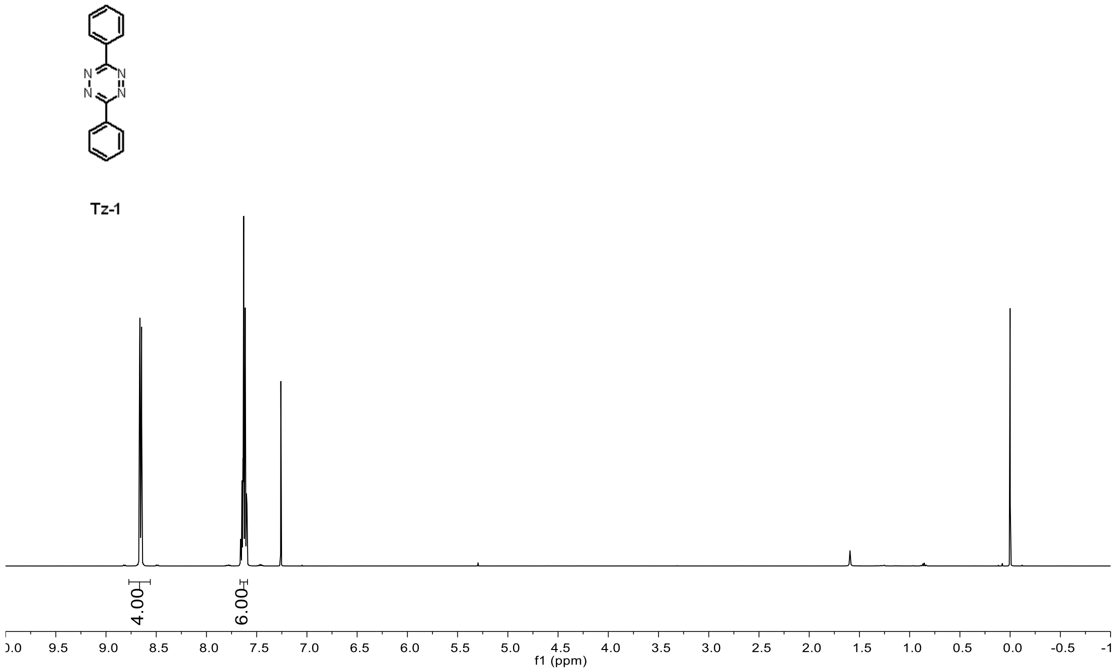

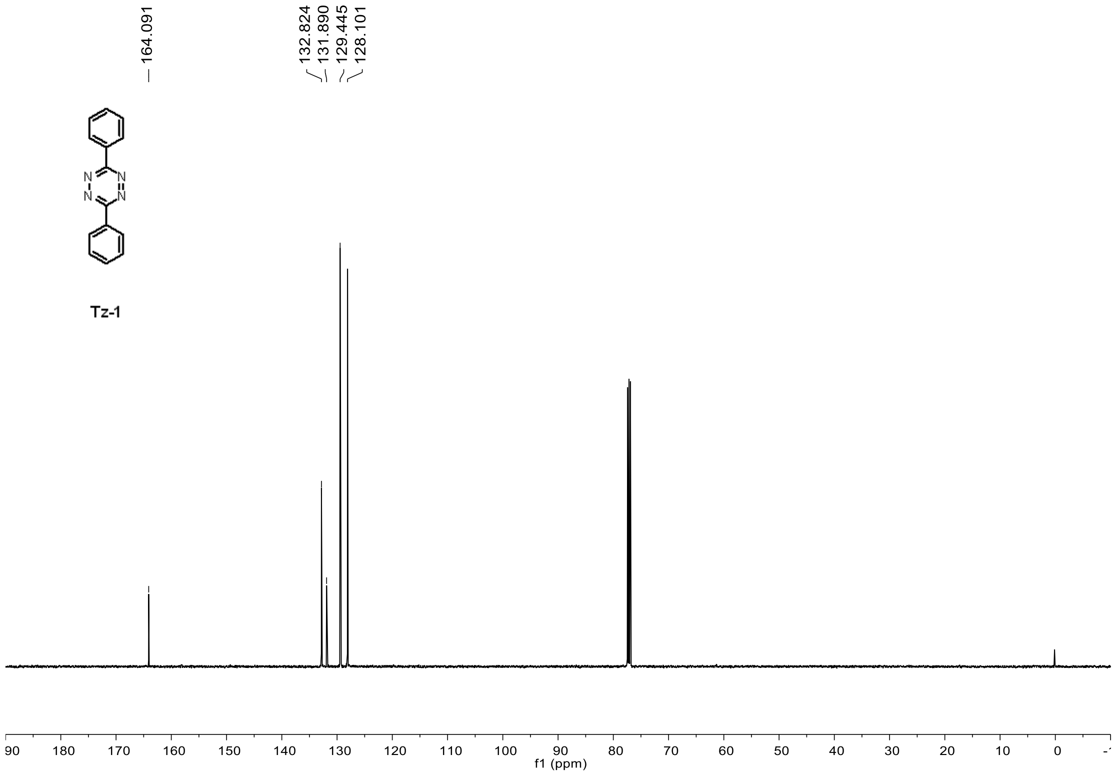

^{1}H NMR and ^{13}C NMR of Tz2

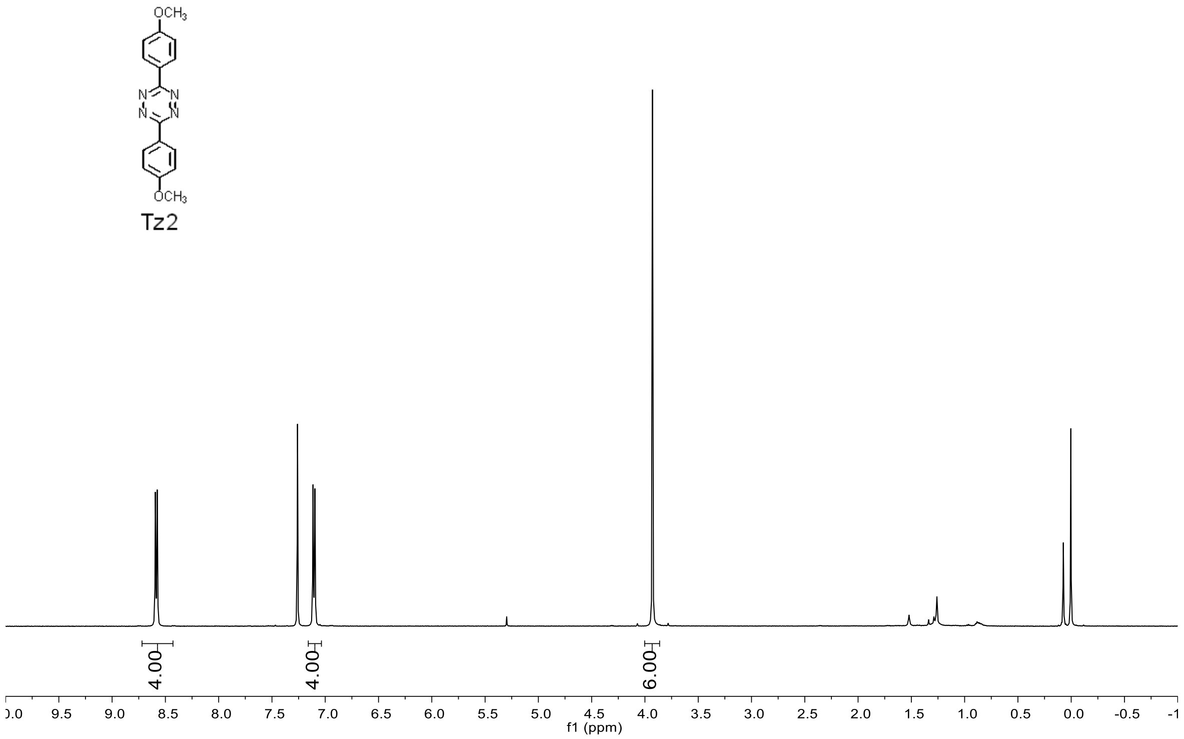

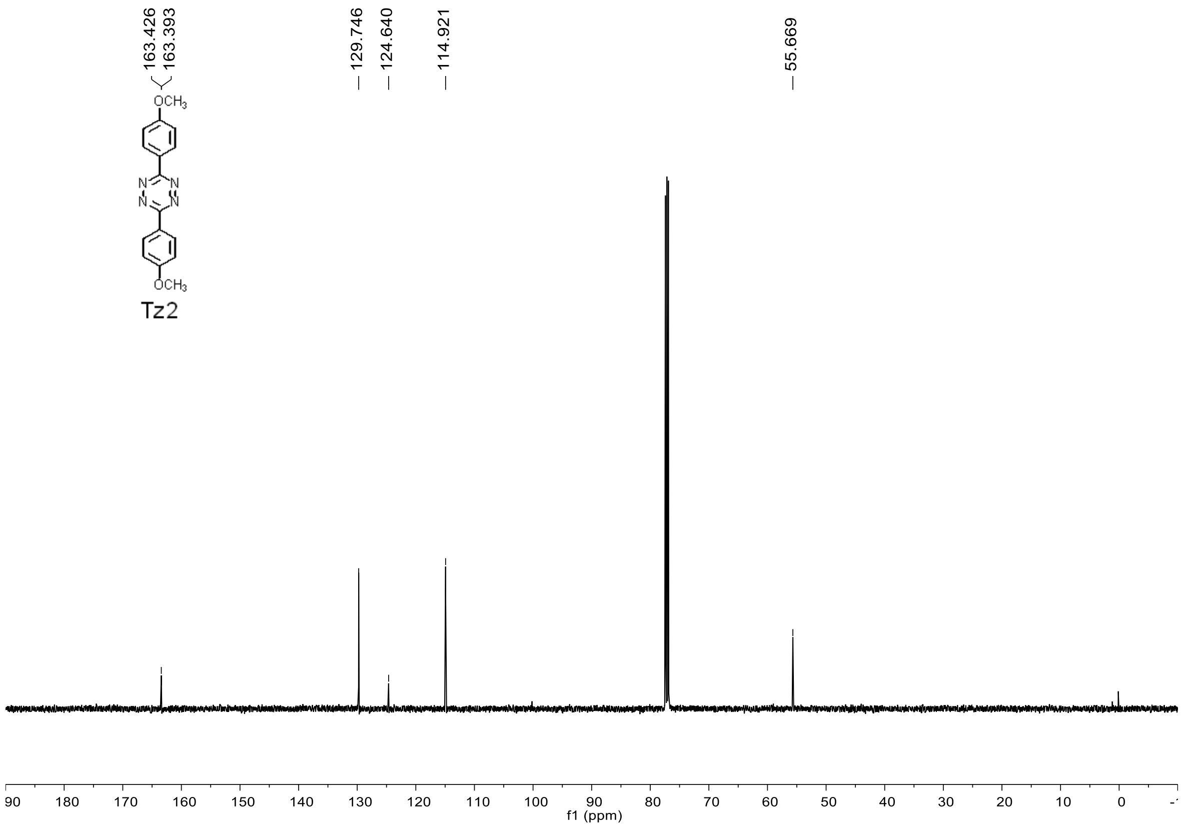

^{1}H NMR and ^{13}C NMR of Tz3

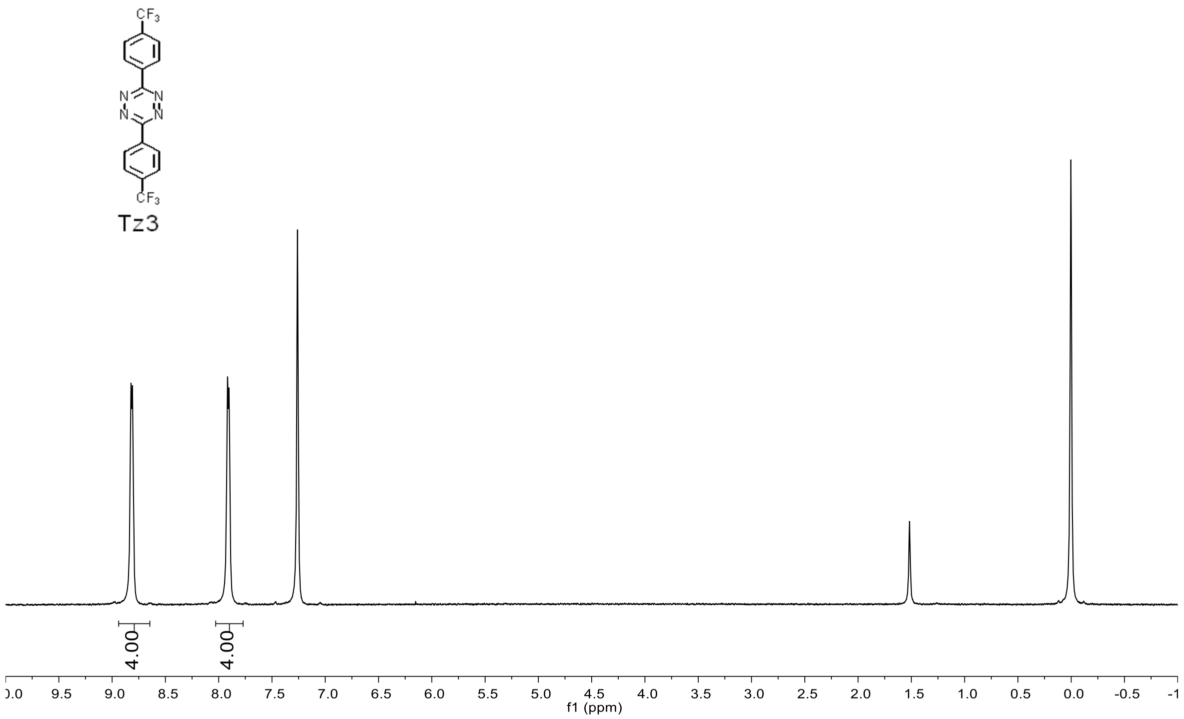

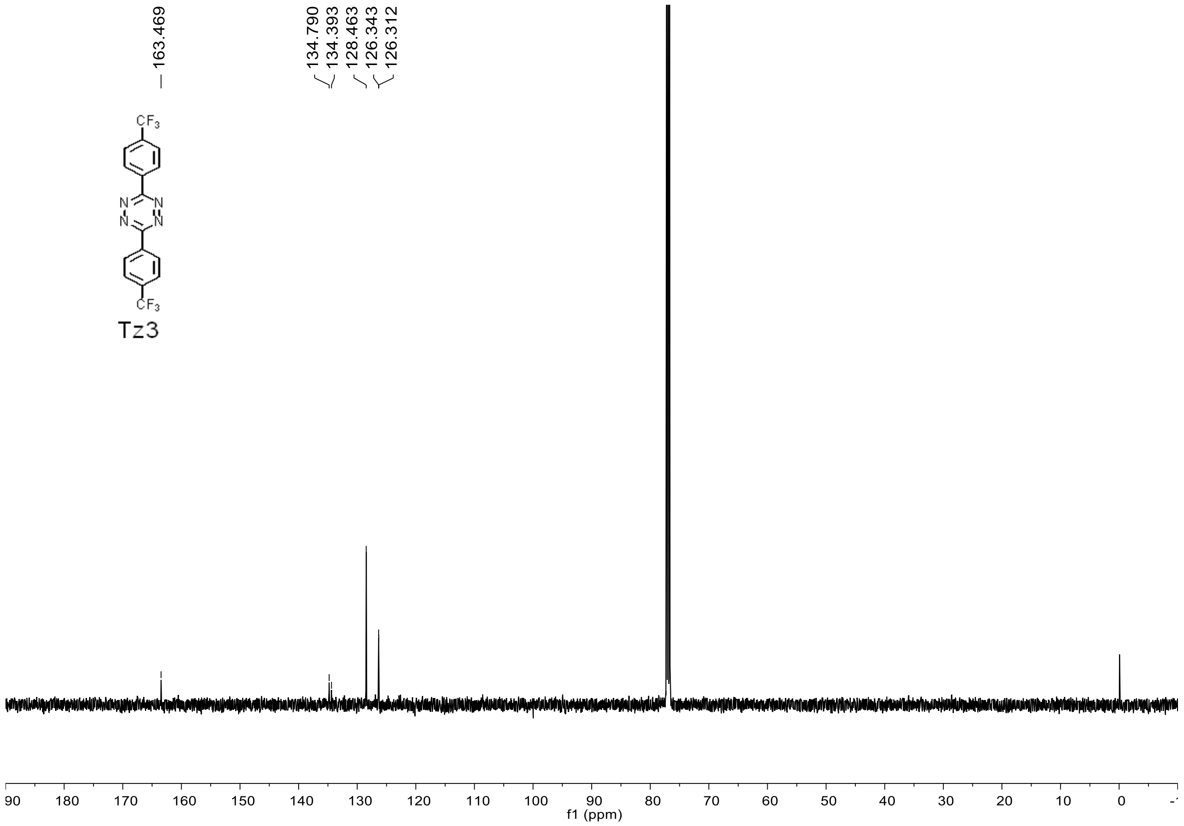

^{1}H NMR and ^{13}C NMR of Tz4

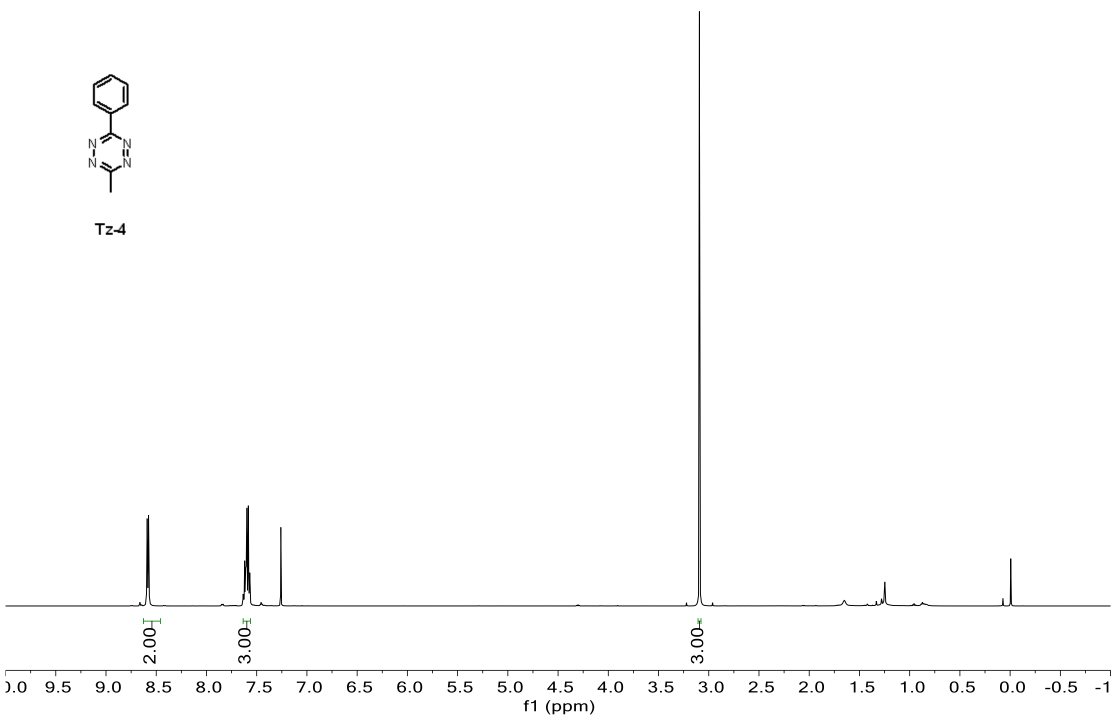

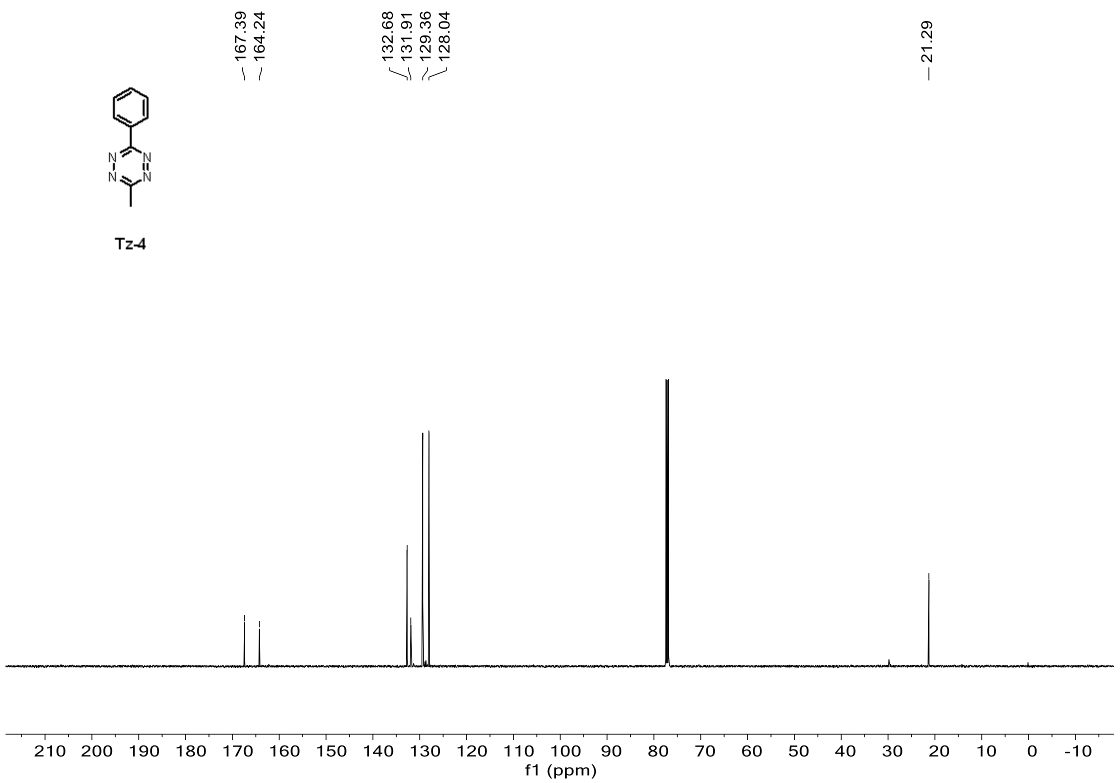

^{1}HNMR and ^{13}C NMR of S1

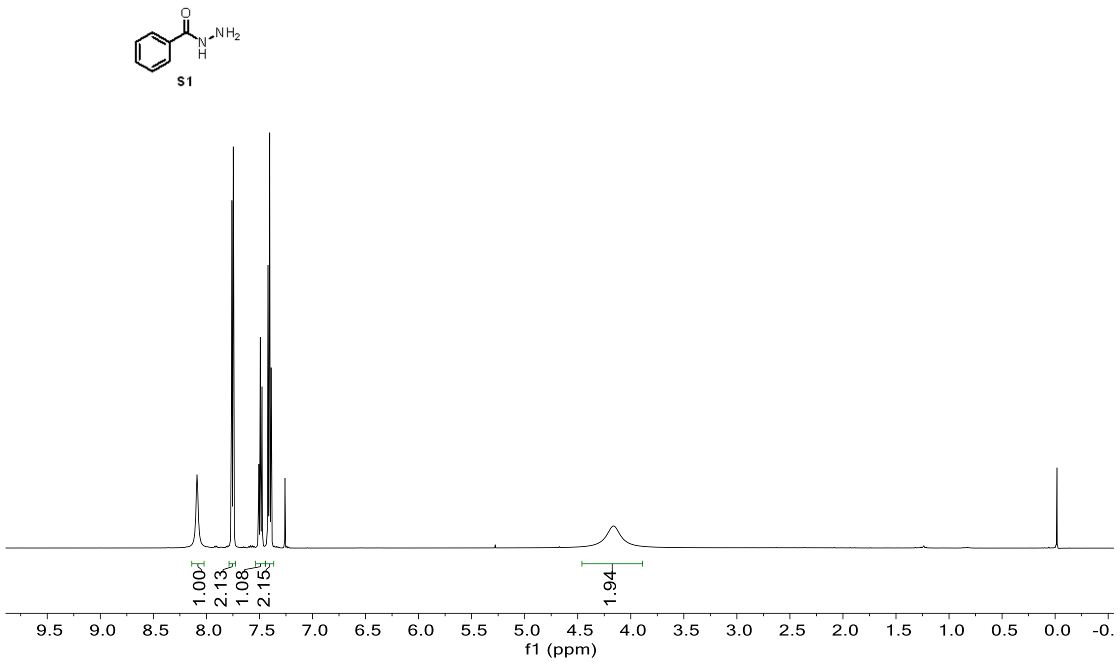

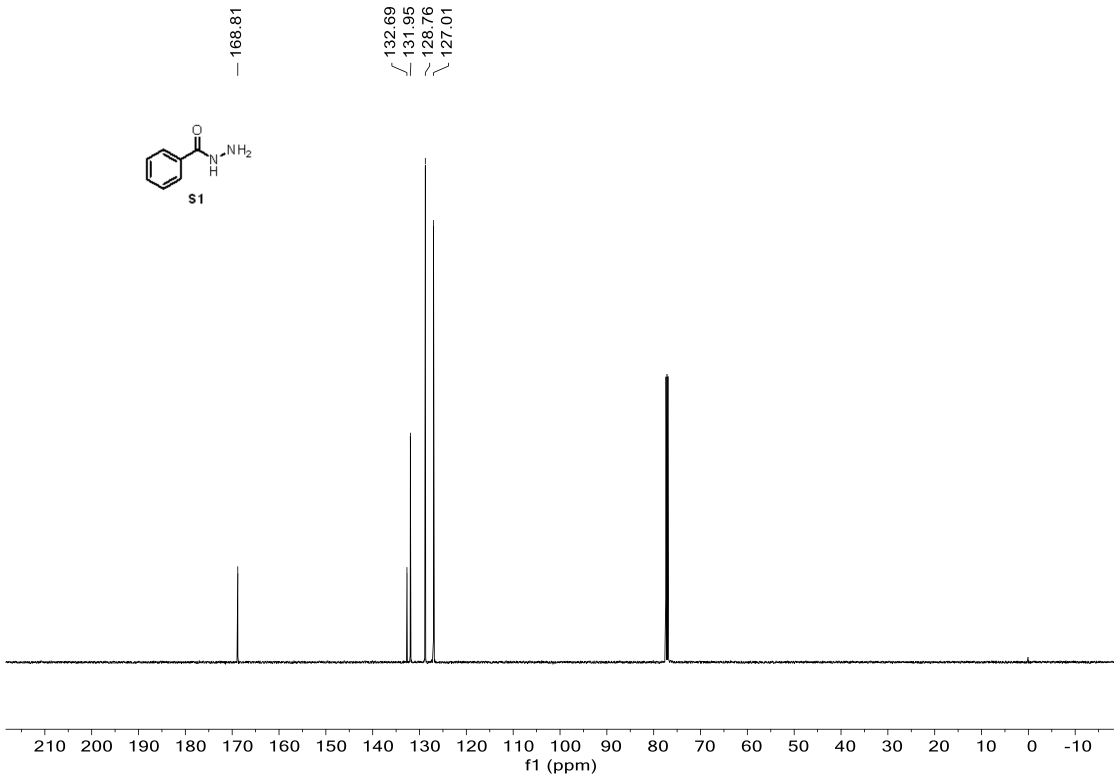

^{1}HNMR and ^{13}C NMR of O1

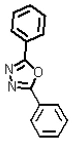

O1

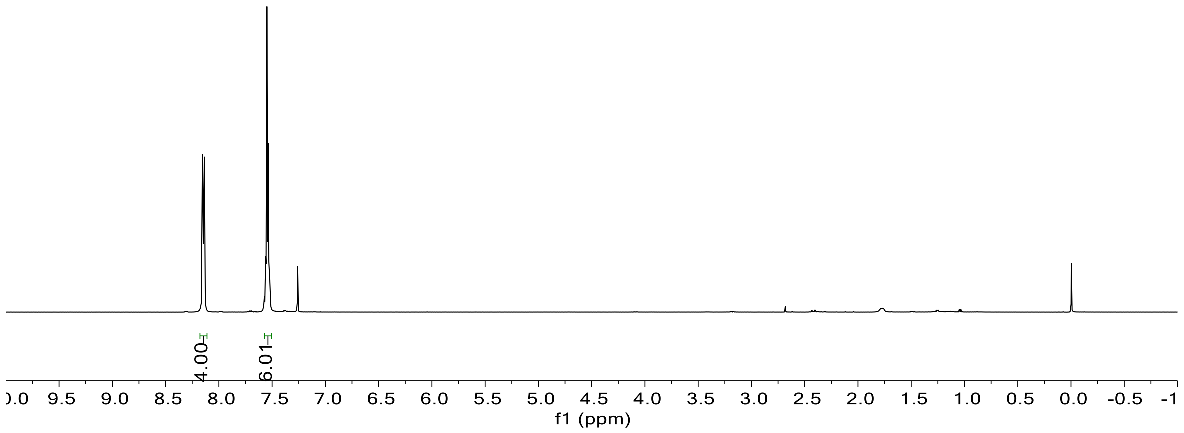

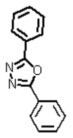

O1

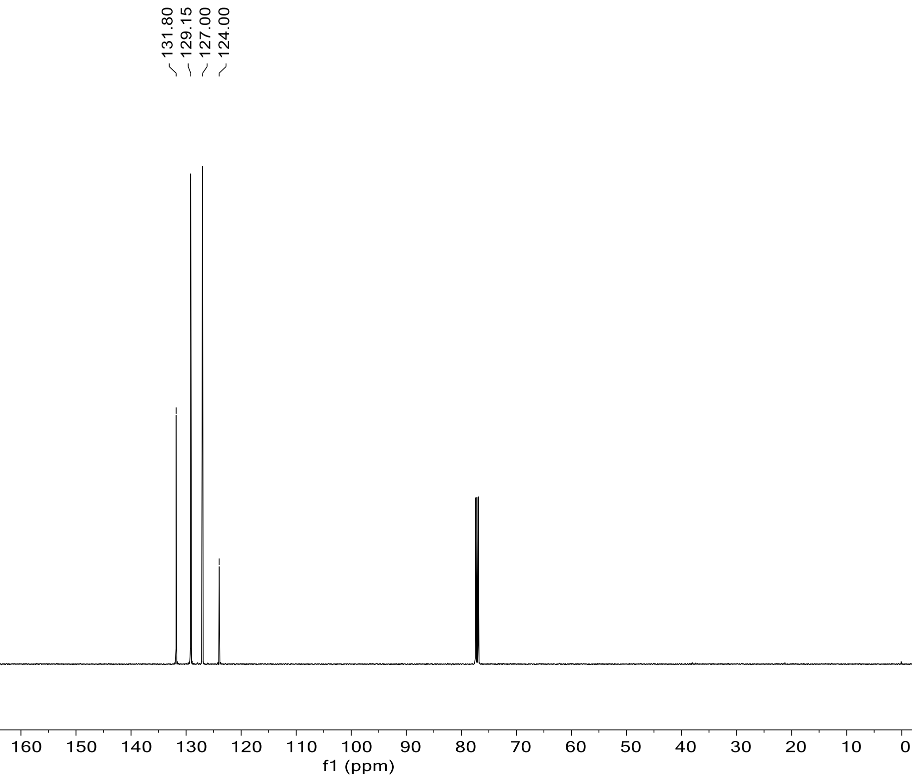

^{1}H NMR and ^{13}C NMR of S2

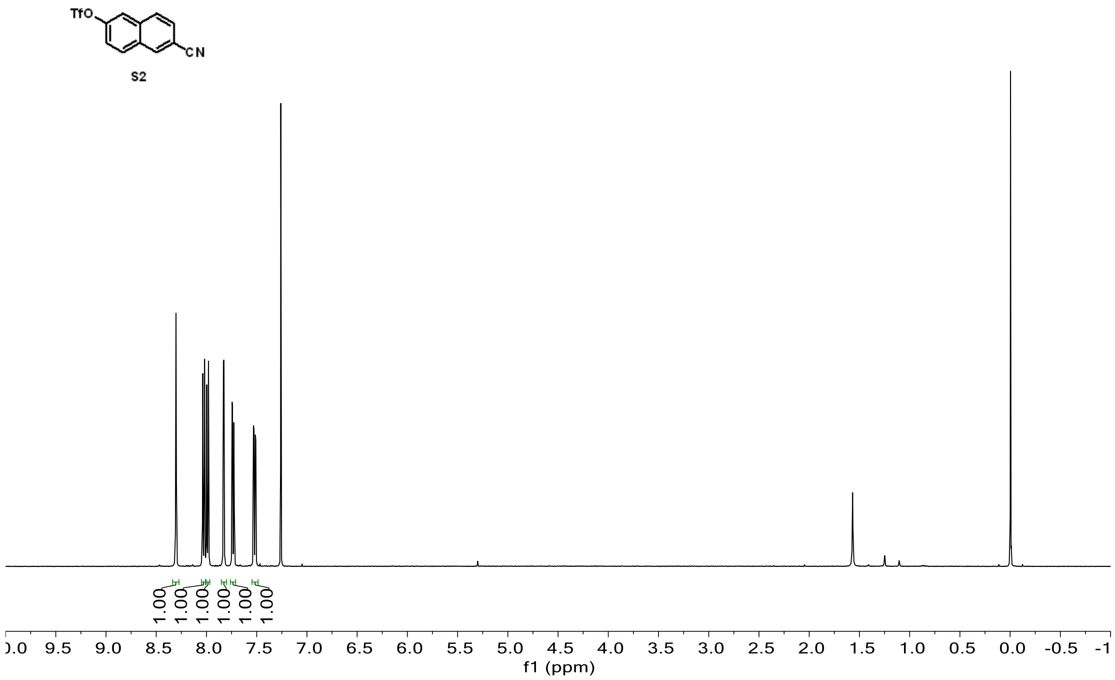

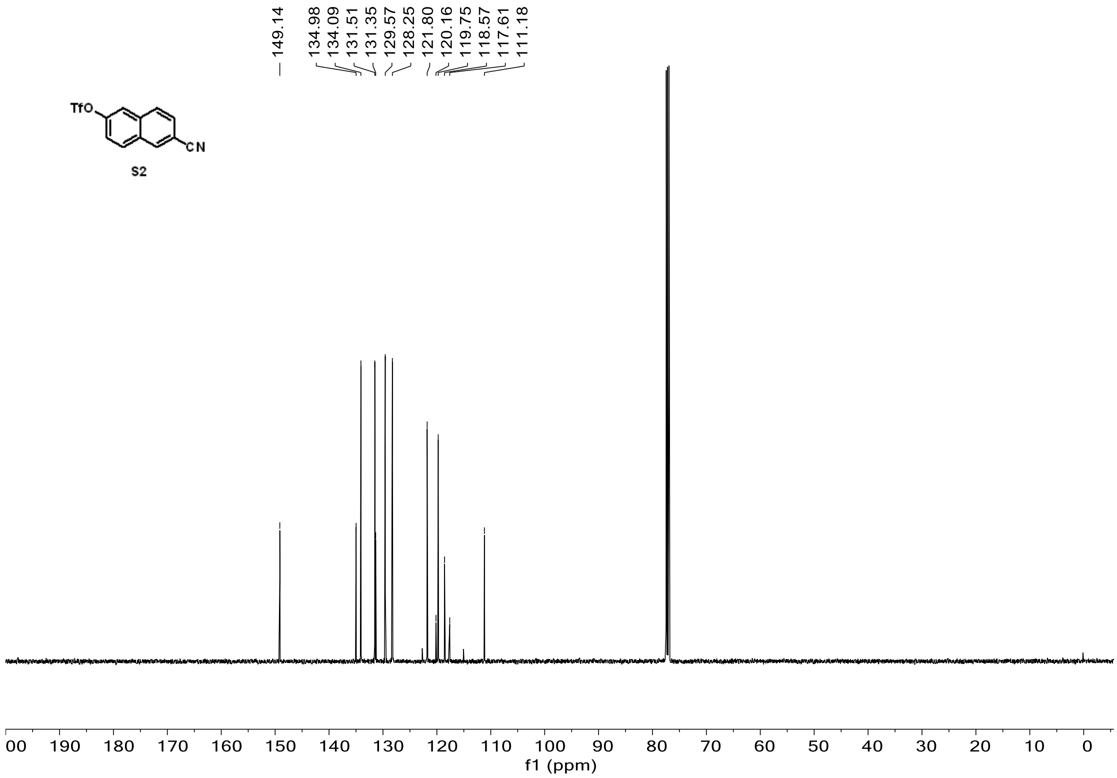

^{1}H NMR and ^{13}C NMR of S4

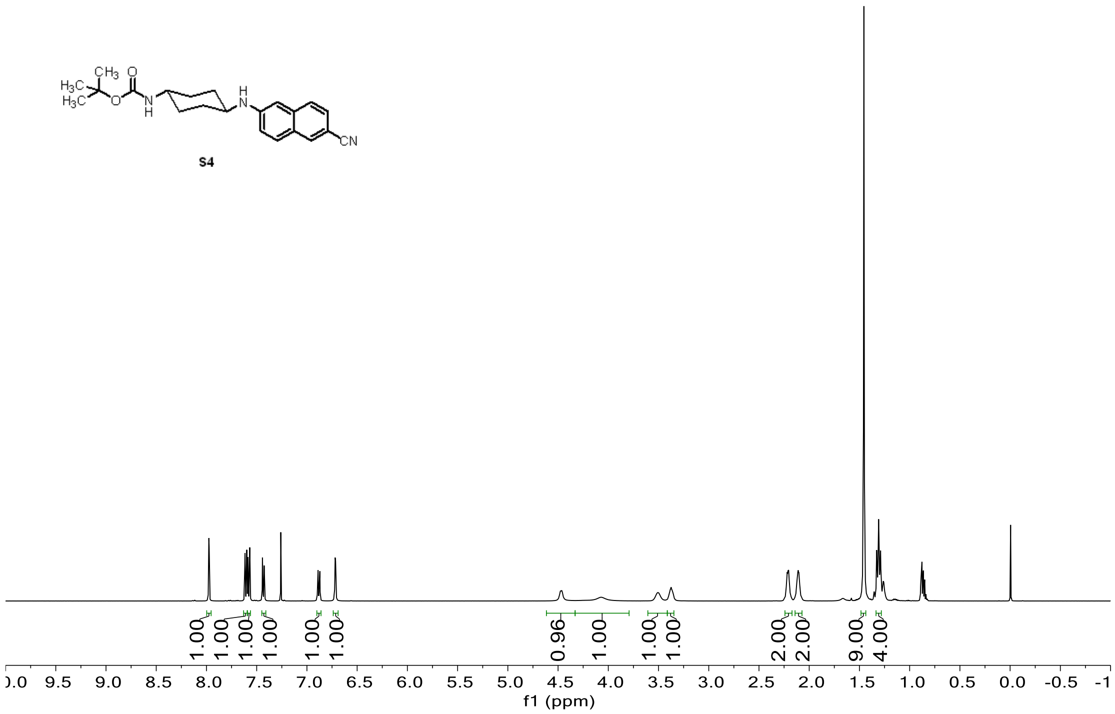

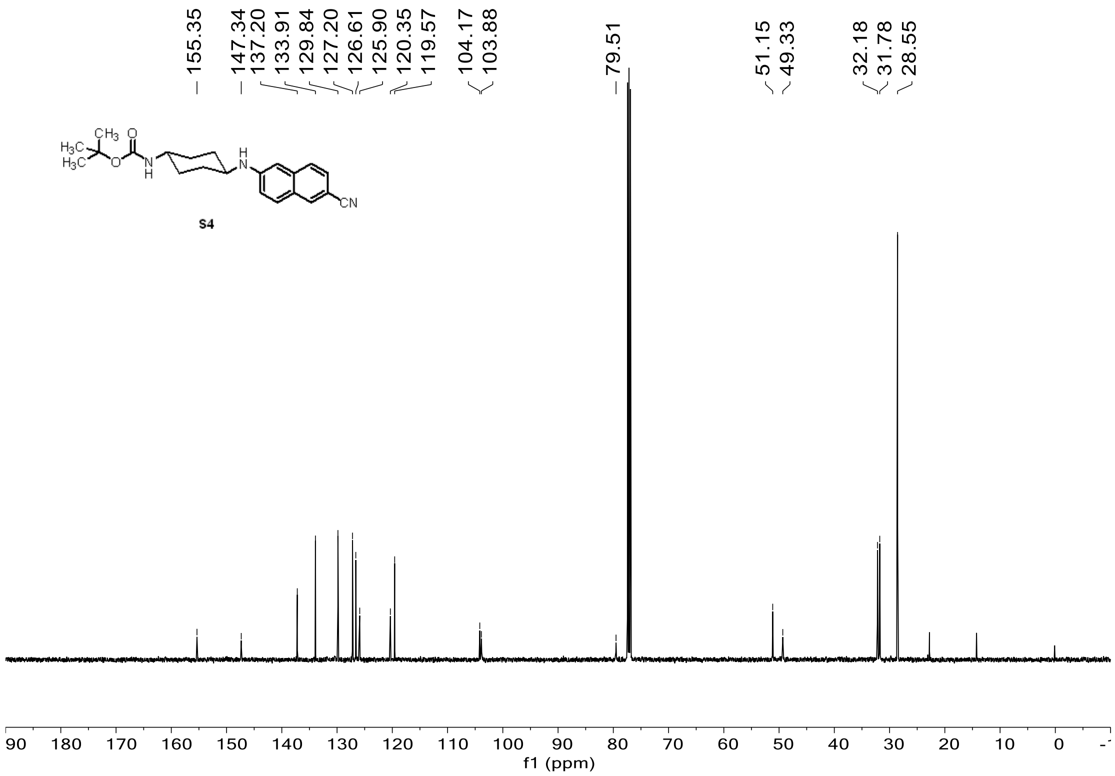

^{1}H NMR and ^{13}C NMR of S6

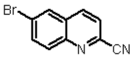

S6

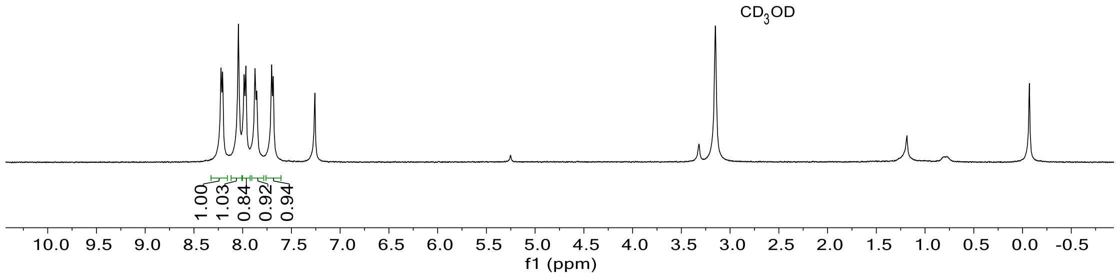

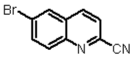

S6

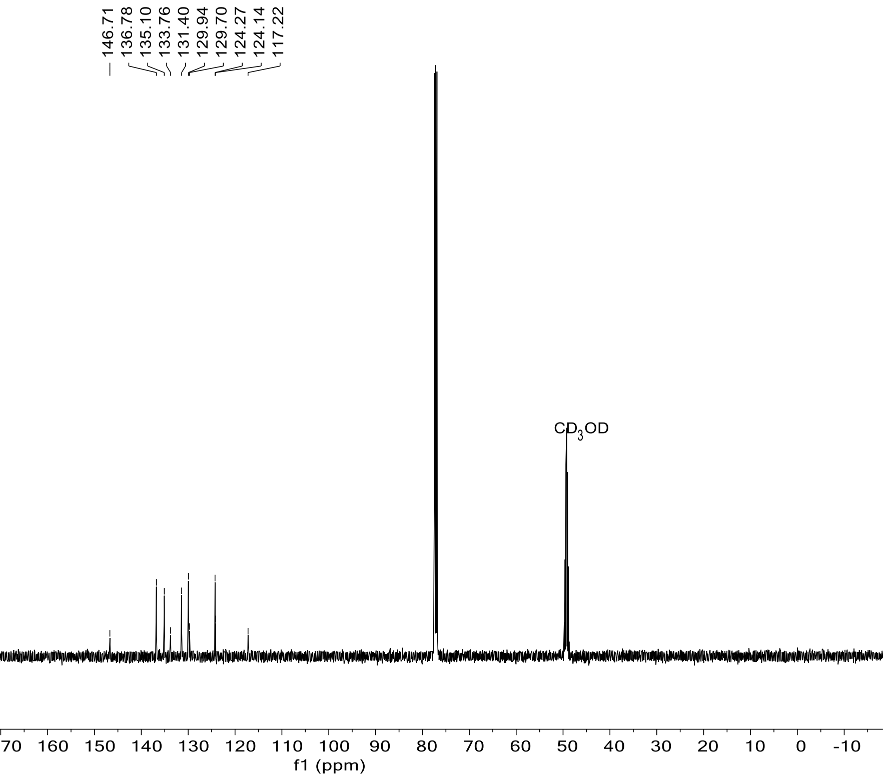

^{1}H NMR and ^{13}C NMR of S7

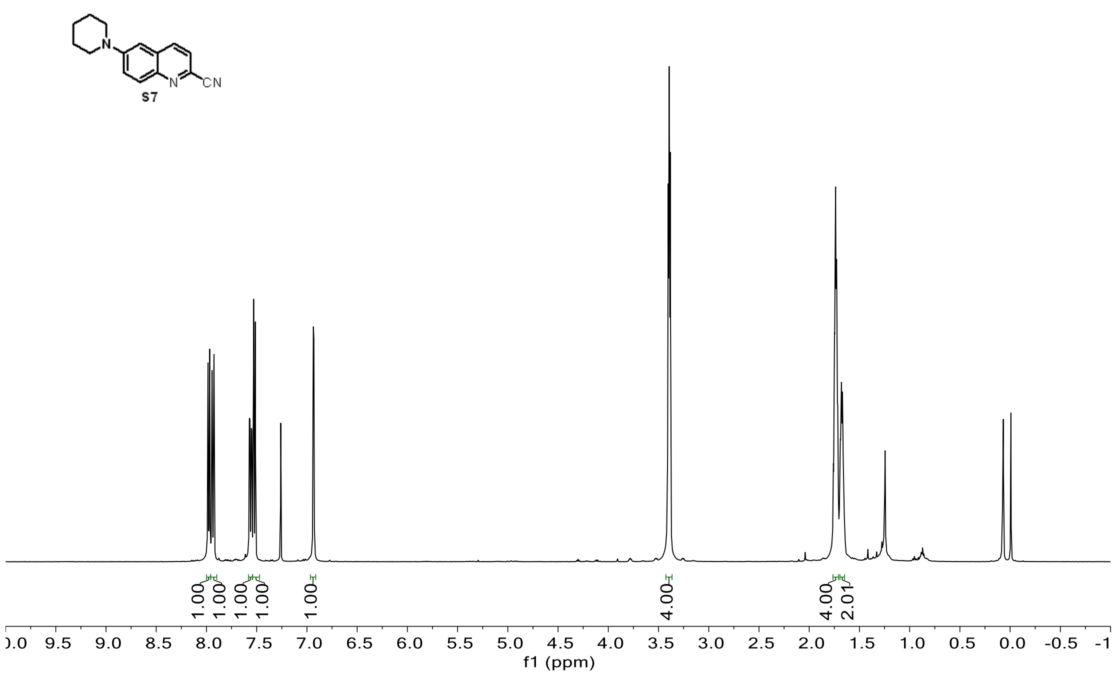

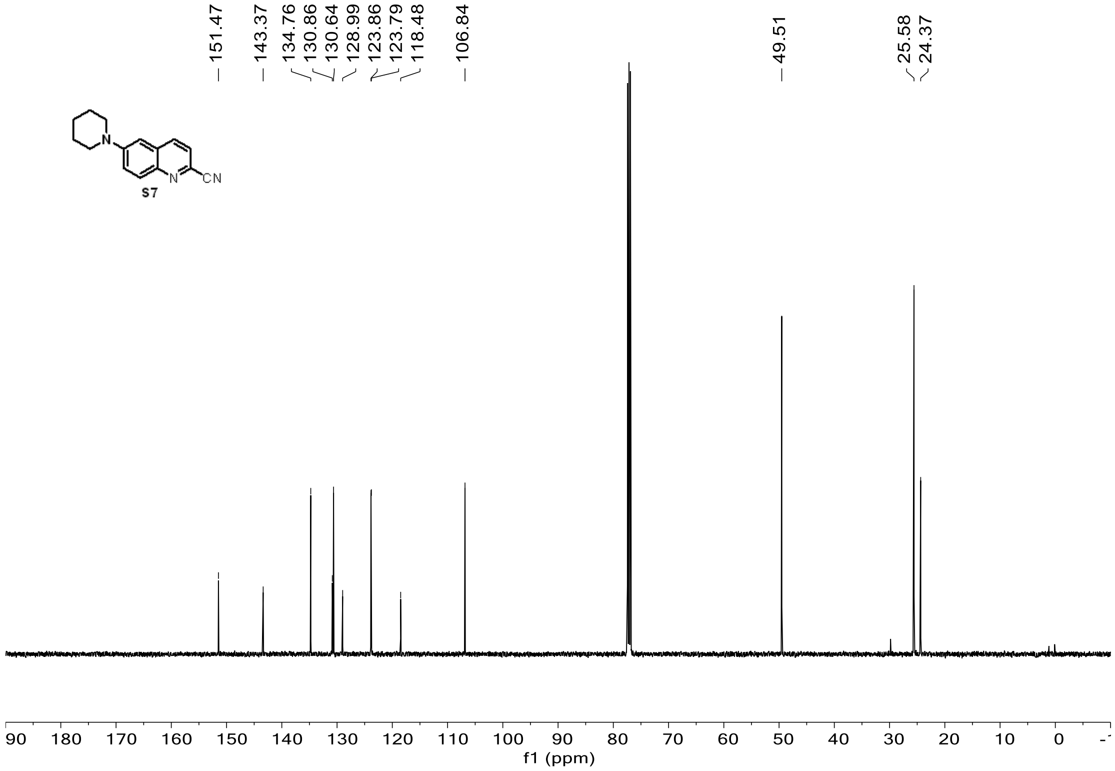

^{1}H NMR and ^{13}C NMR of S8

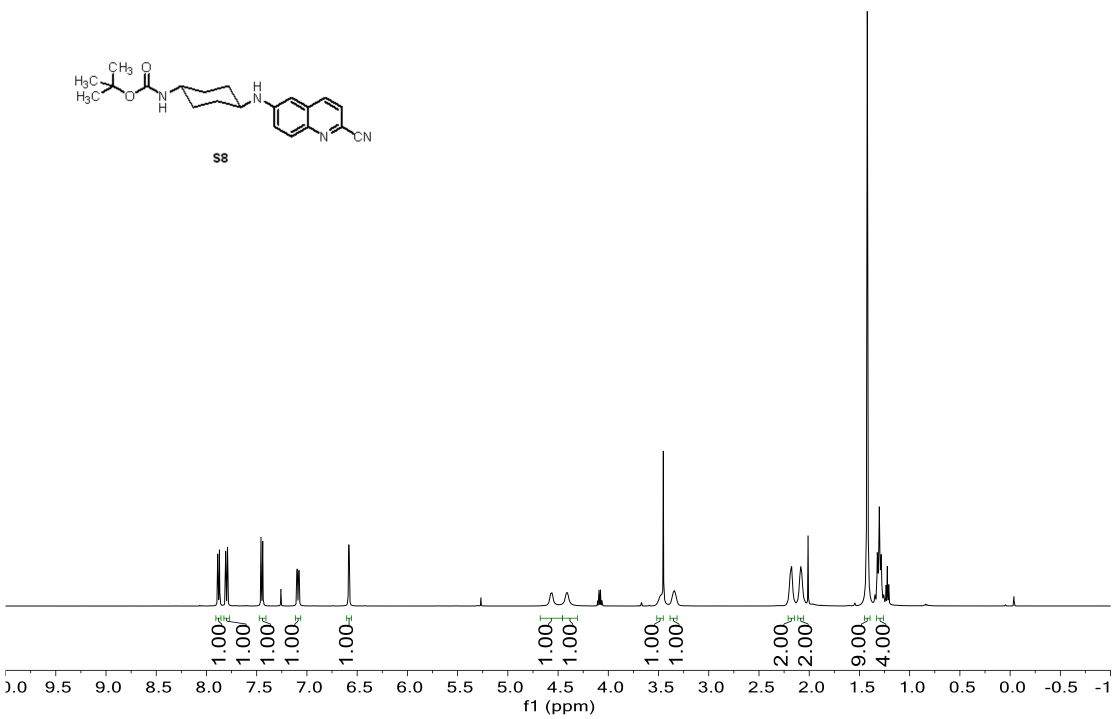

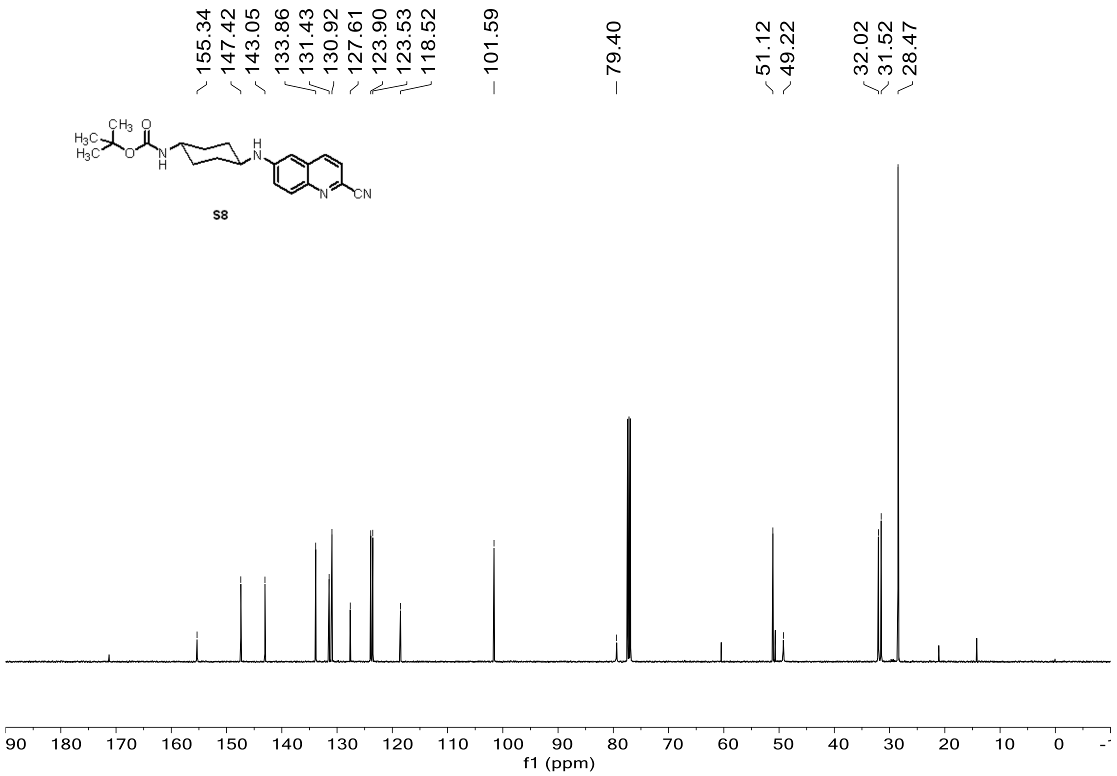

^{1}H NMR and ^{13}C NMR of F-Tz1

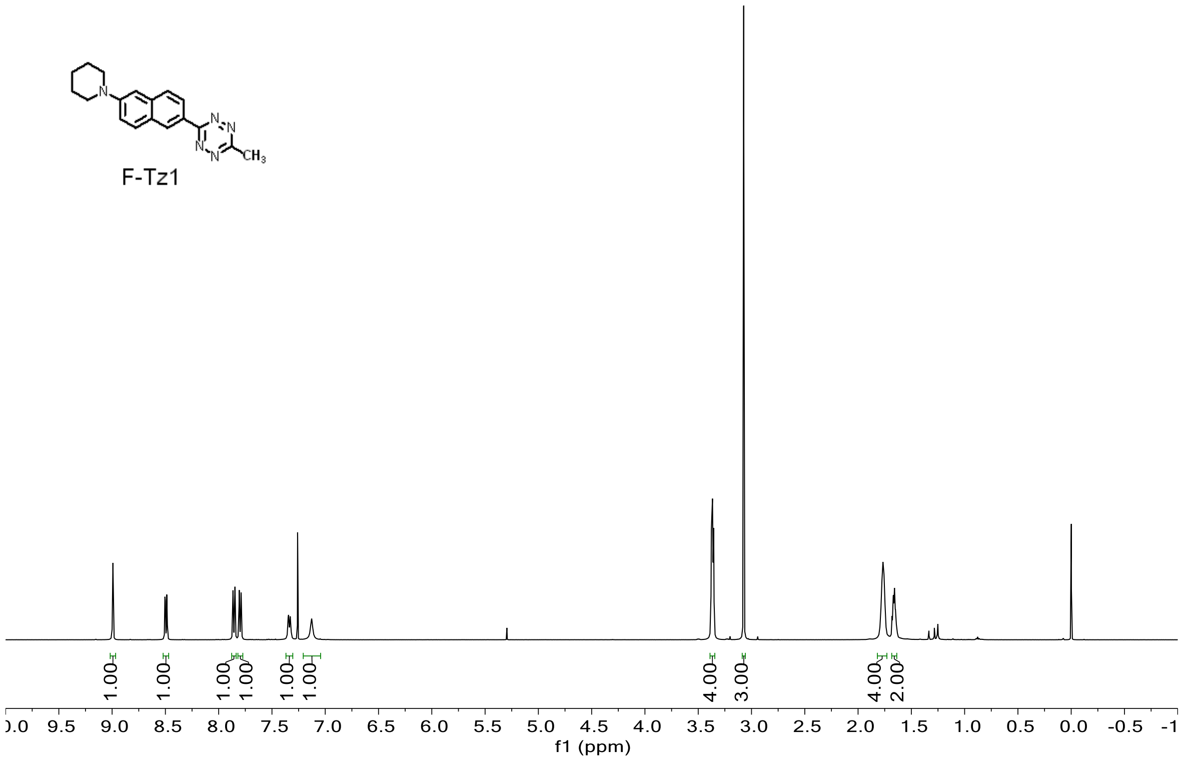

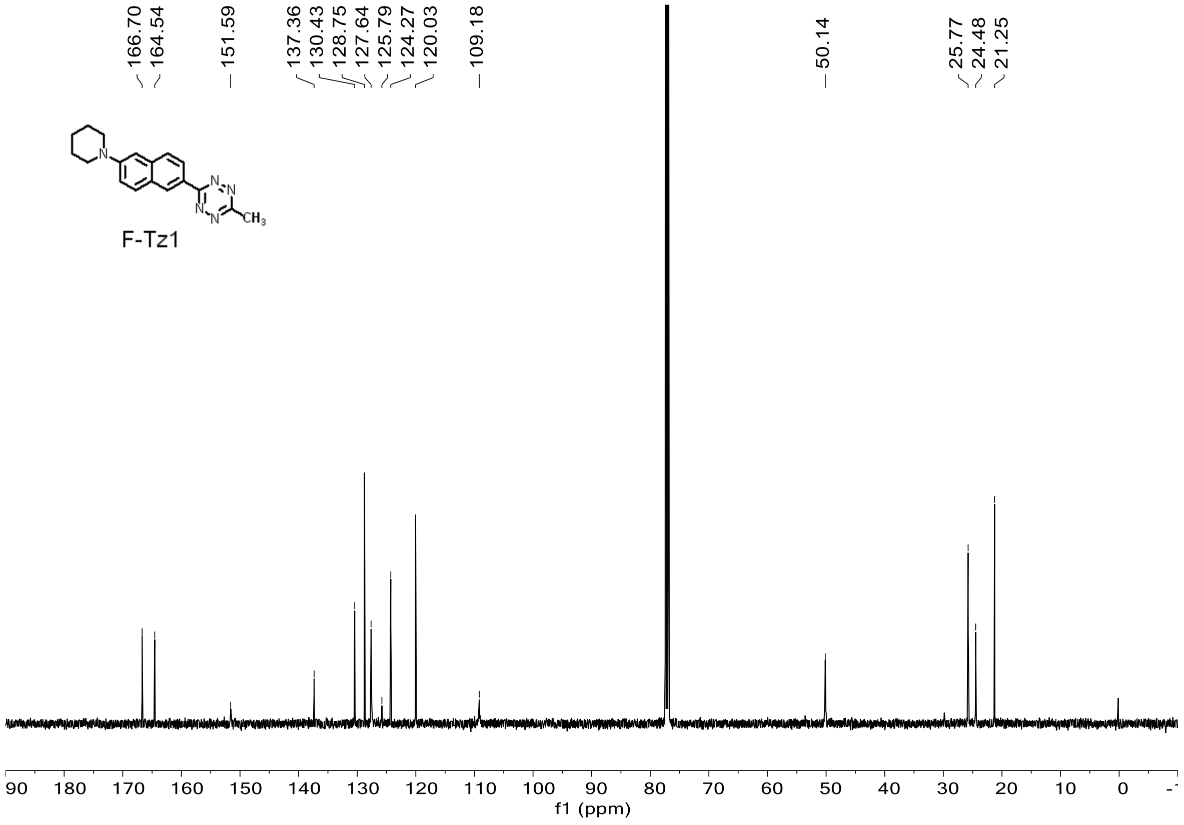

^{1}H NMR and ^{13}C NMR of F-Tz2

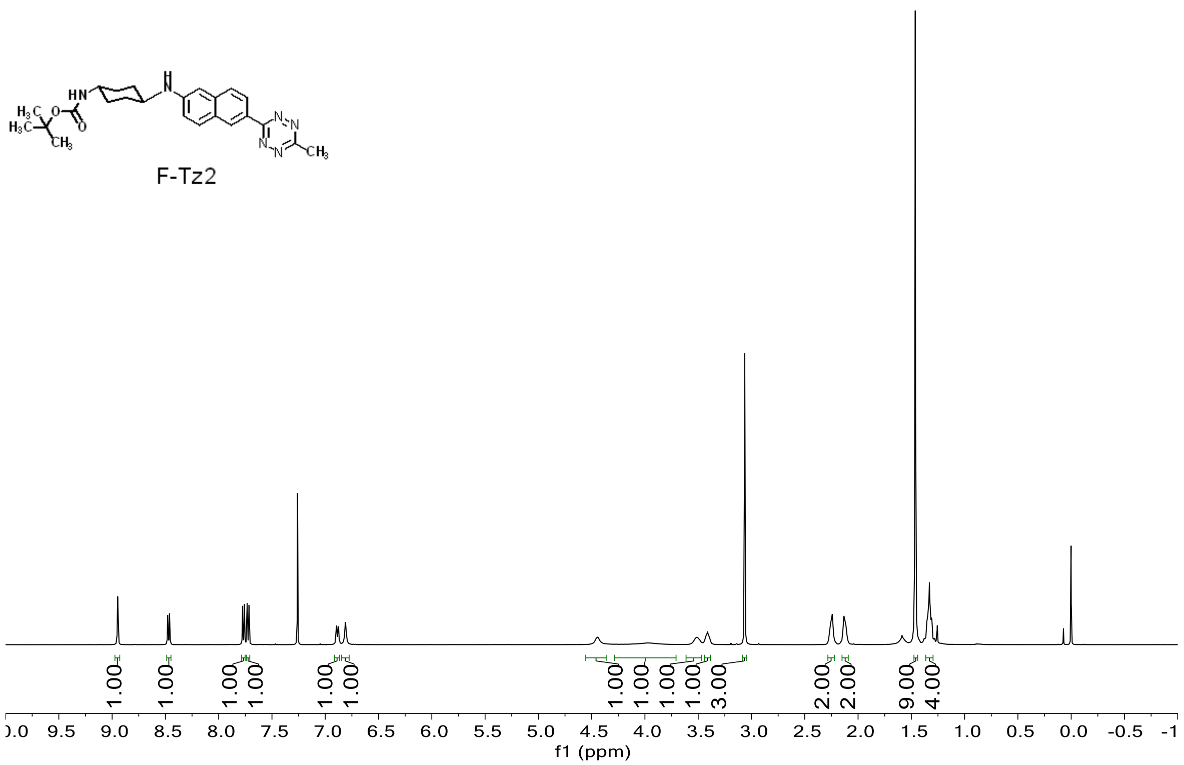

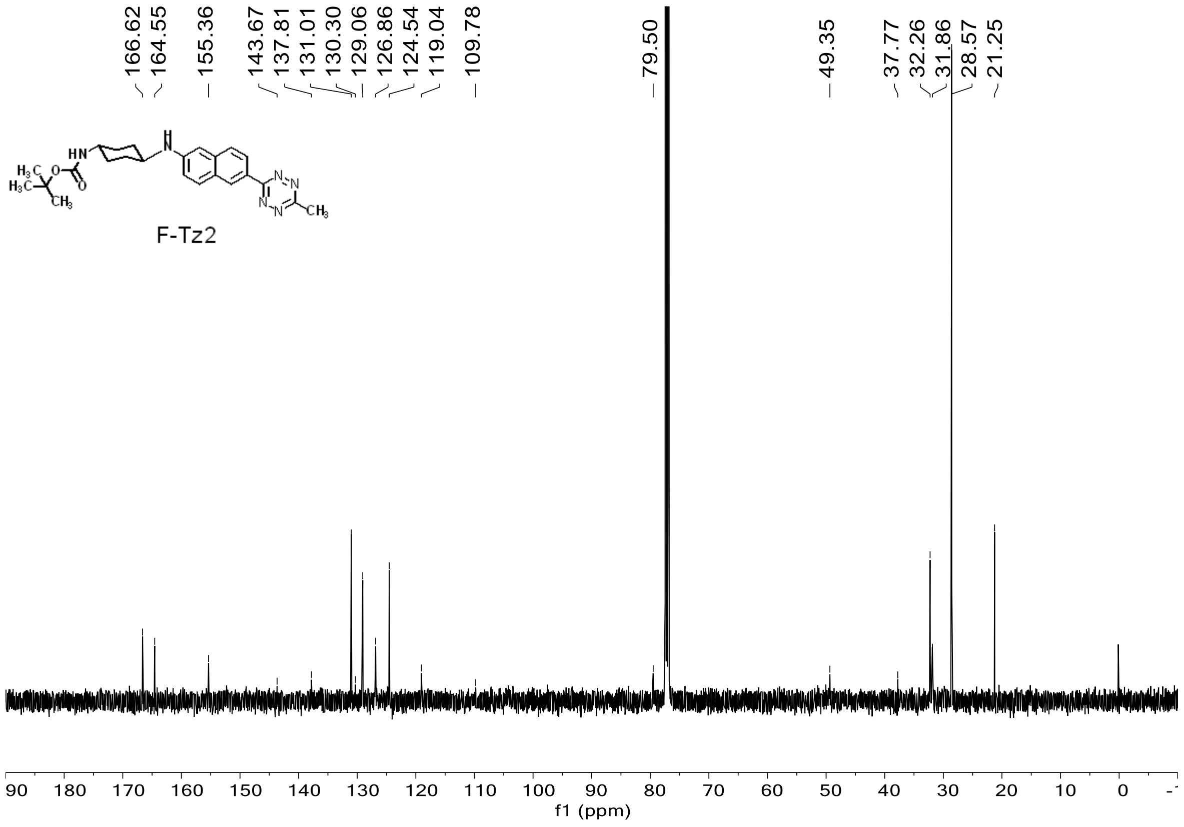

^{1}H NMR and ^{13}C NMR of F-Tz3

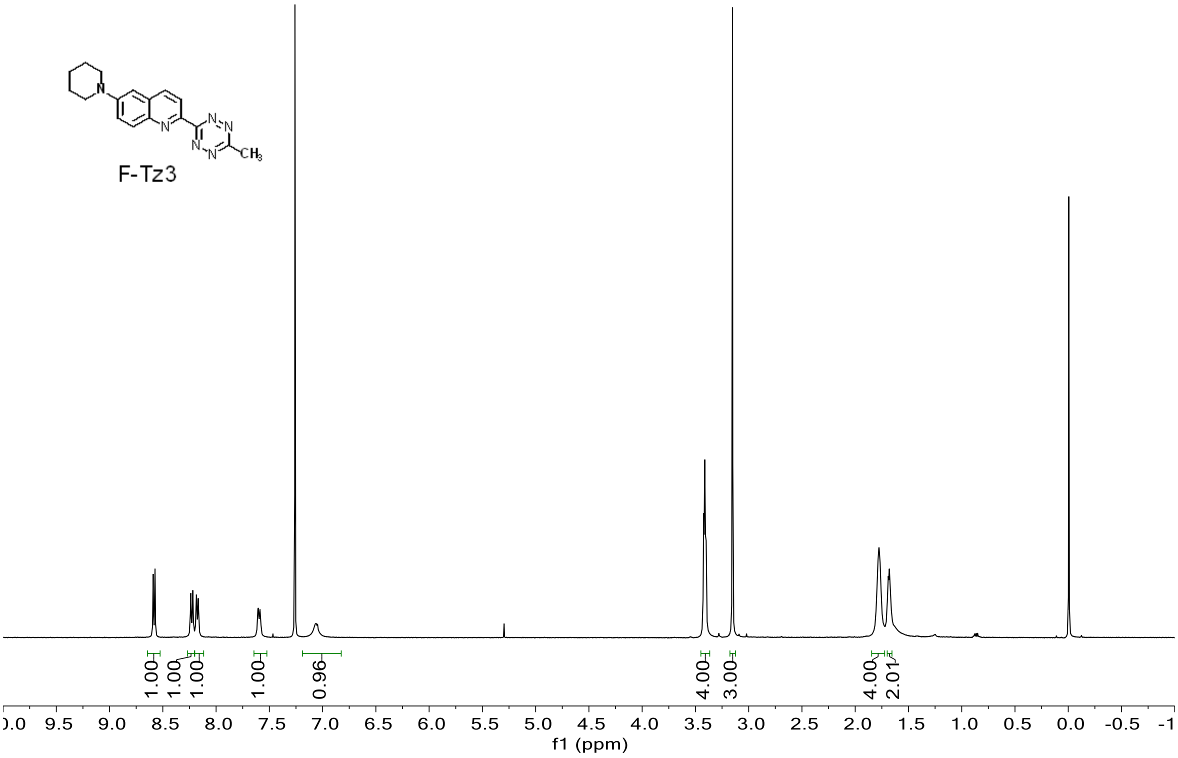

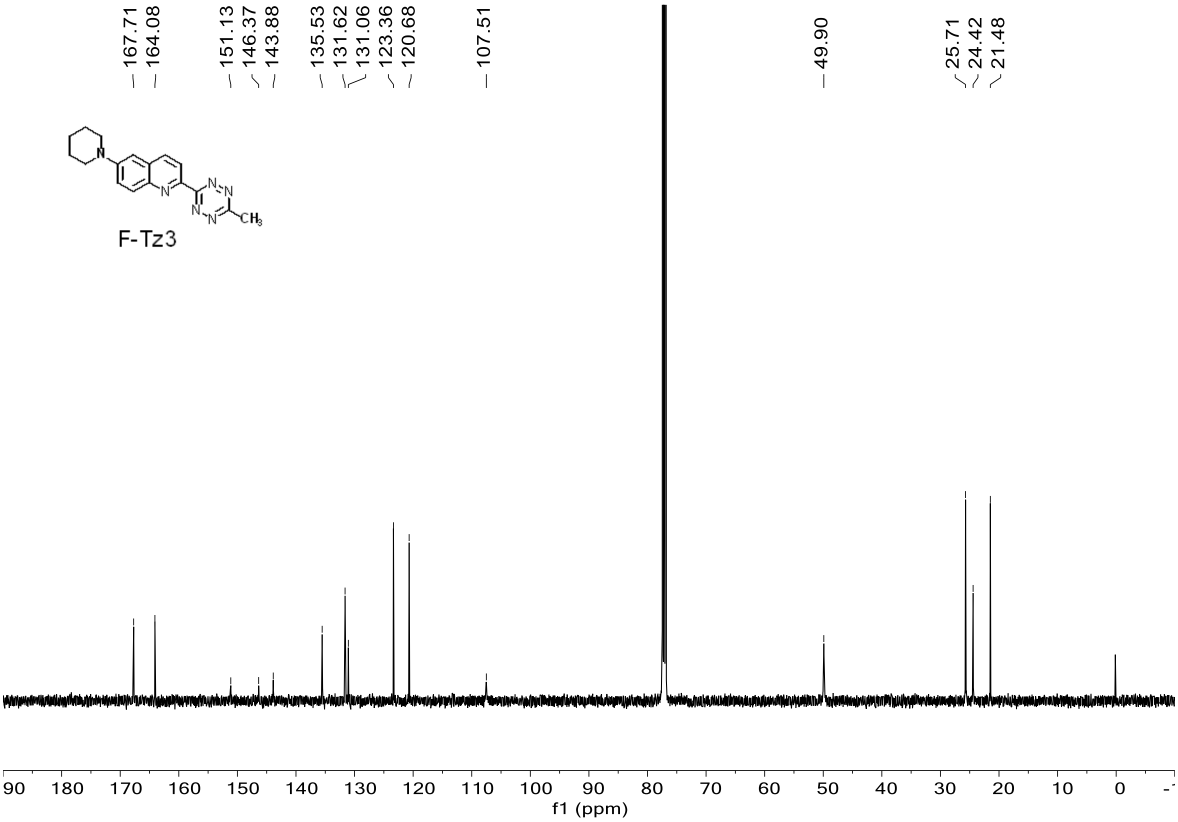

^{1}H NMR and ^{13}C NMR of F-Tz4

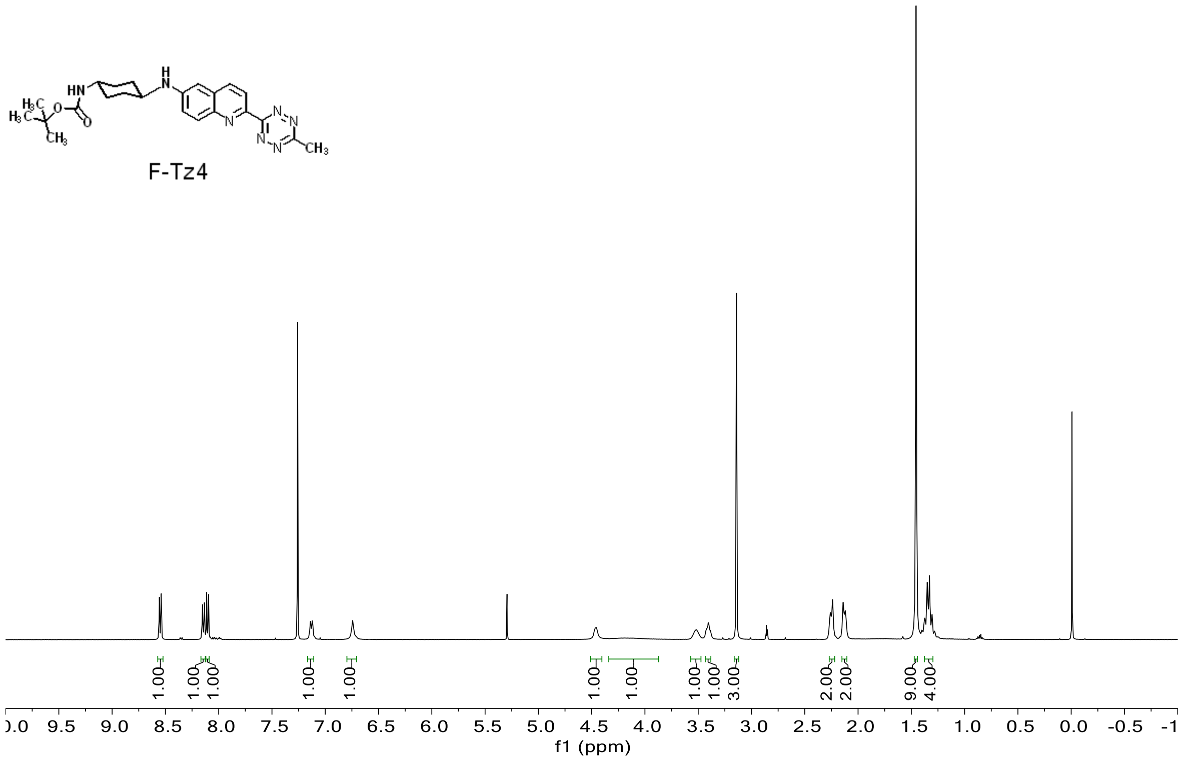

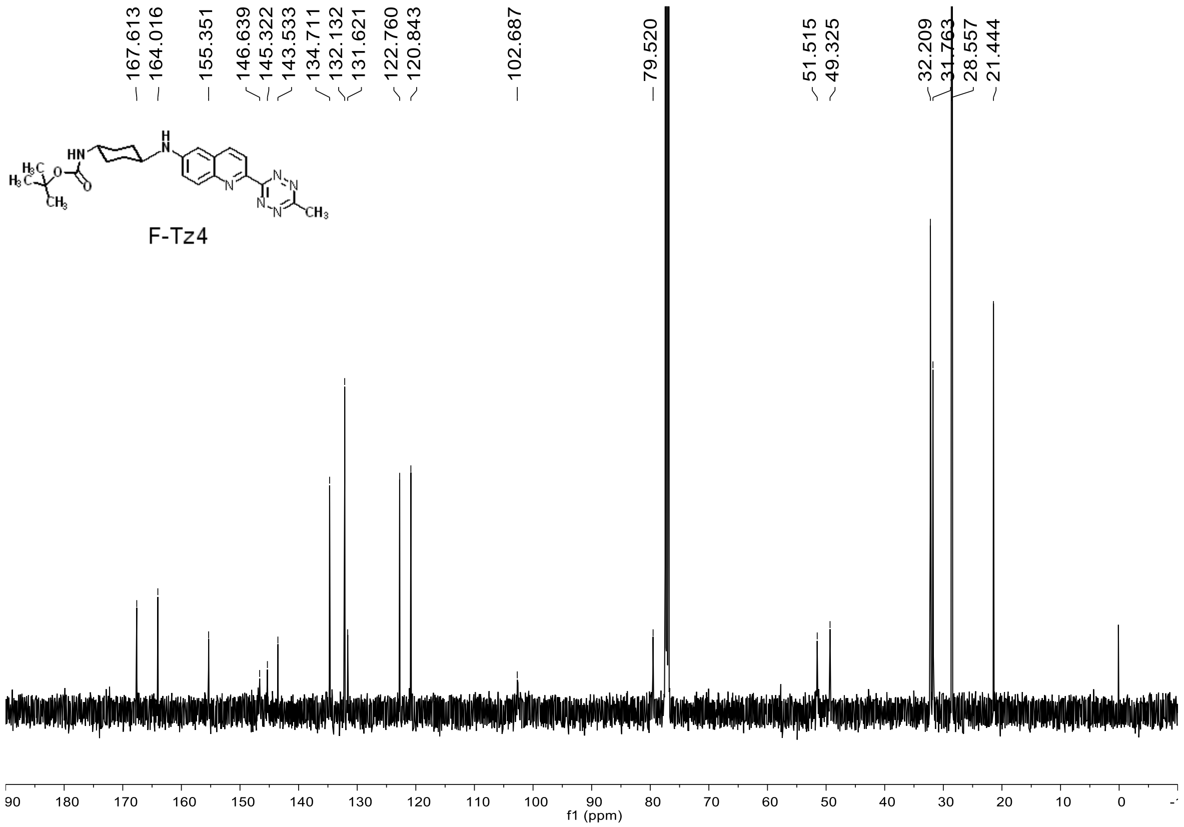

^{1}H NMR and ^{13}C NMR of F-Tz5

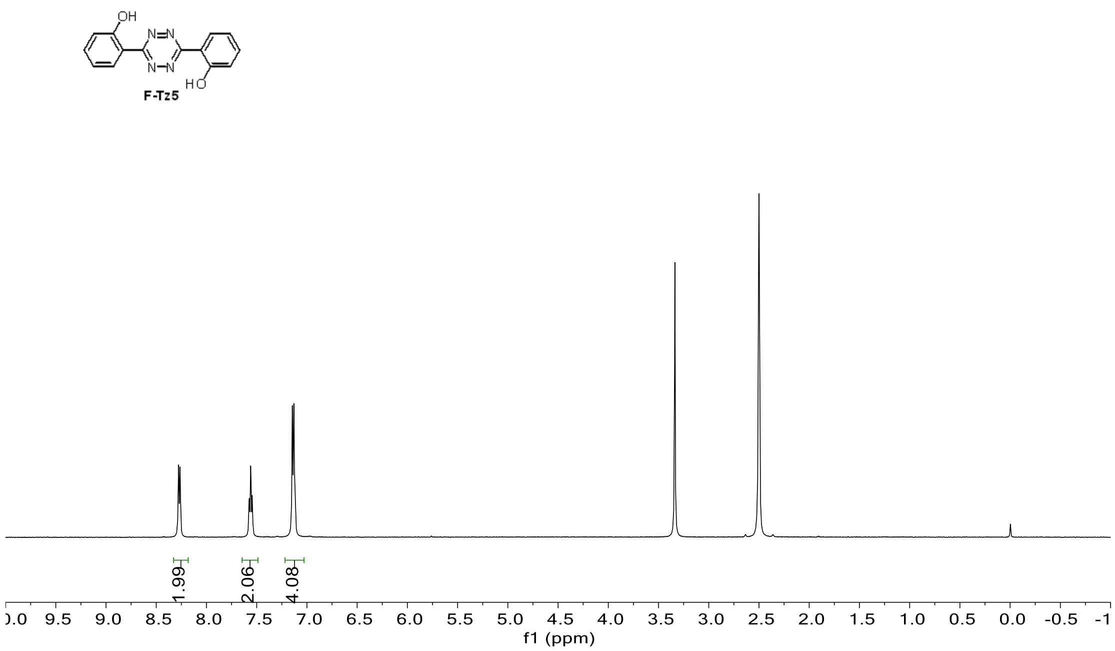

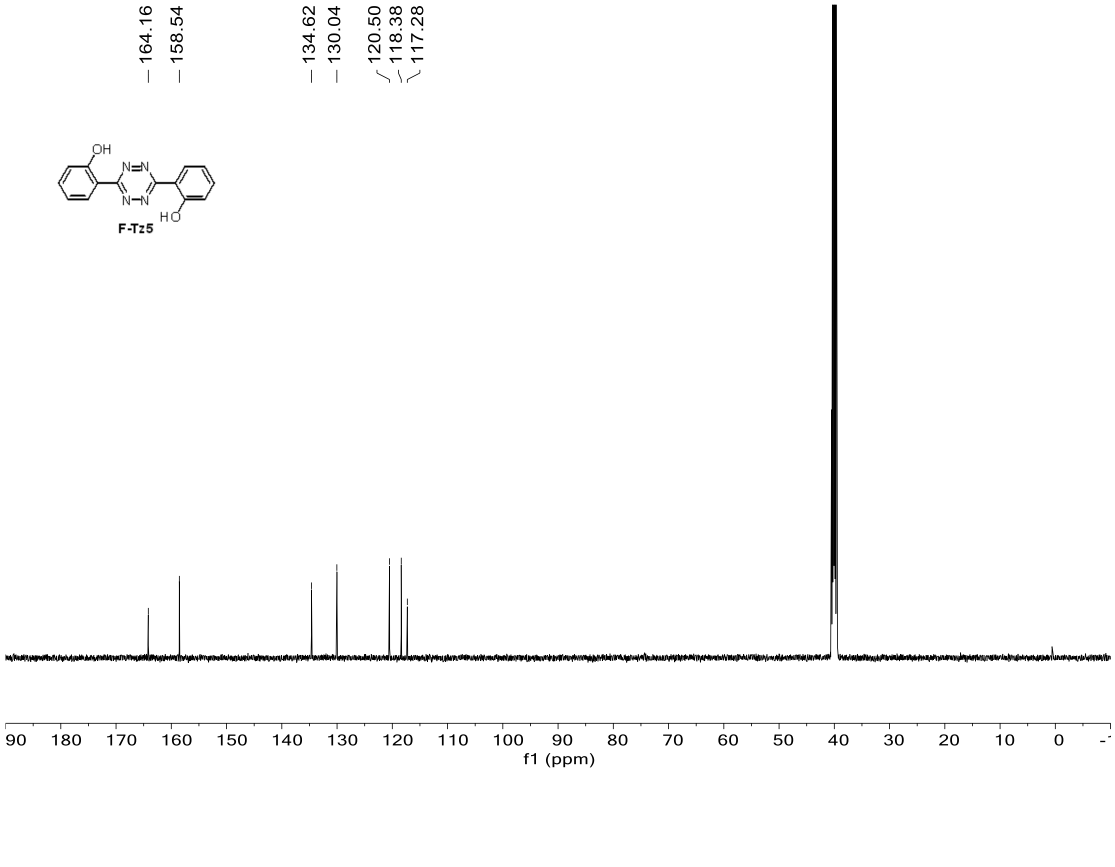

^{1}H NMR and ^{13}C NMR of S11

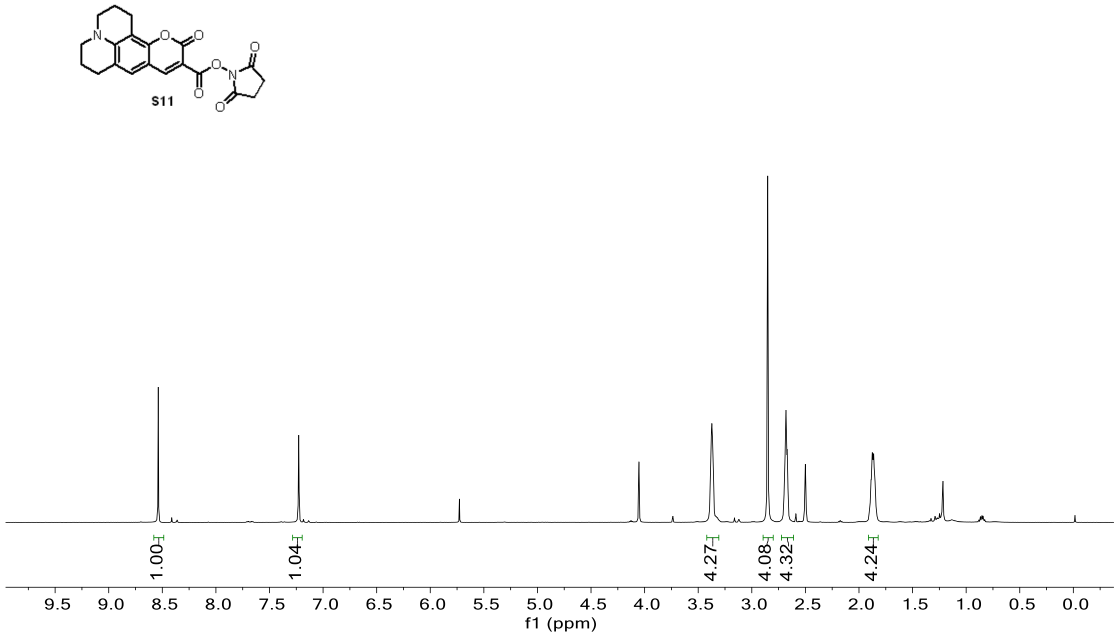

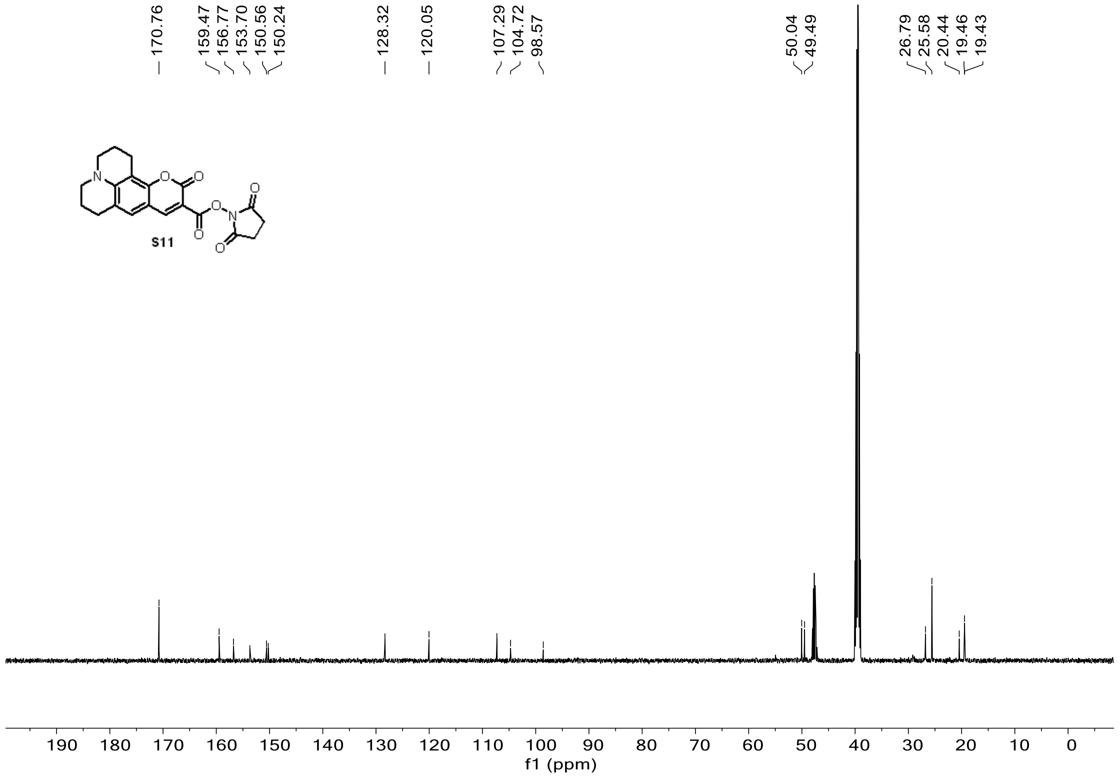

^{1}H NMR and ^{13}C NMR of F-Tz6

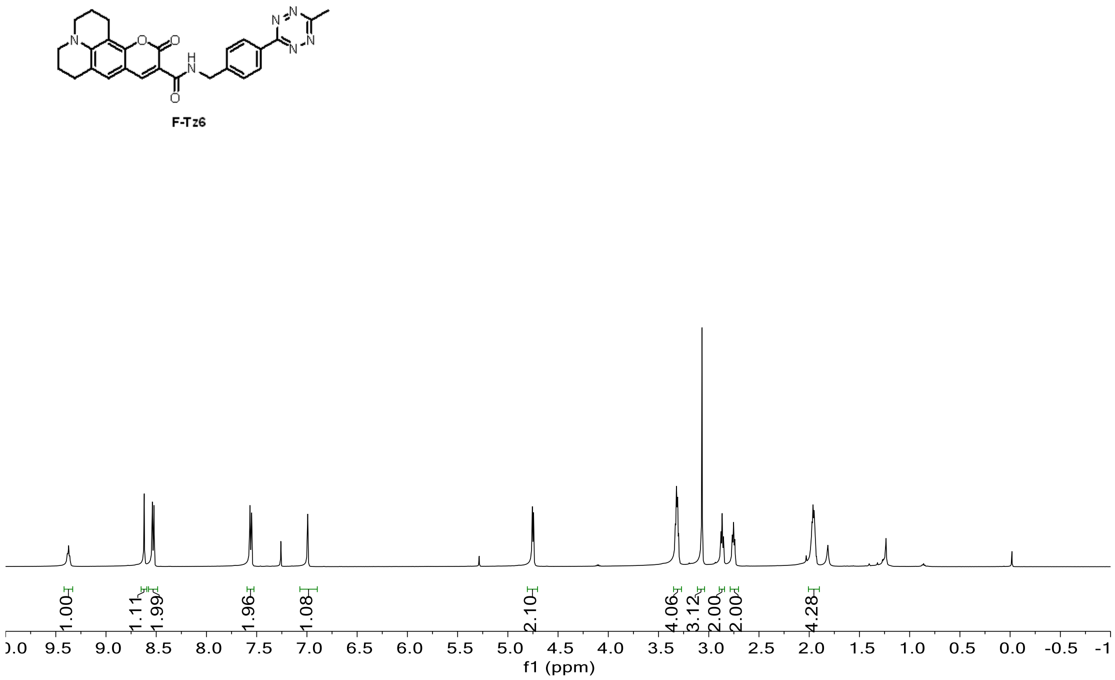

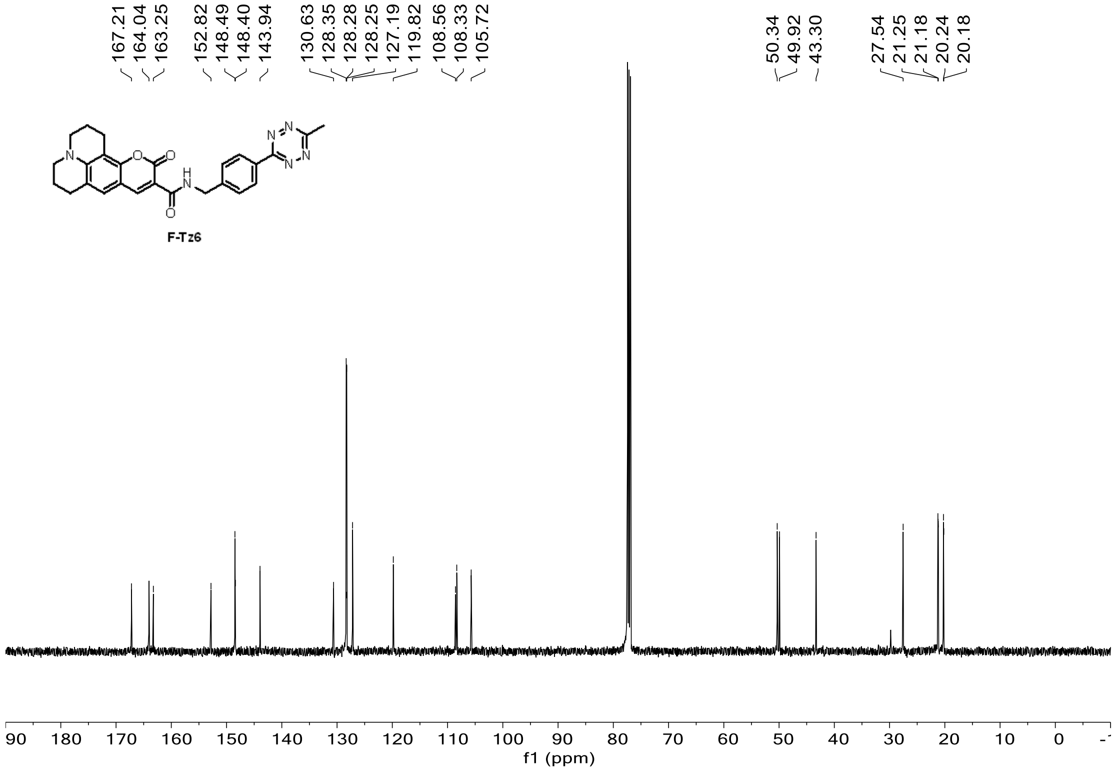

^{1}H NMR and ^{13}C NMR of S14

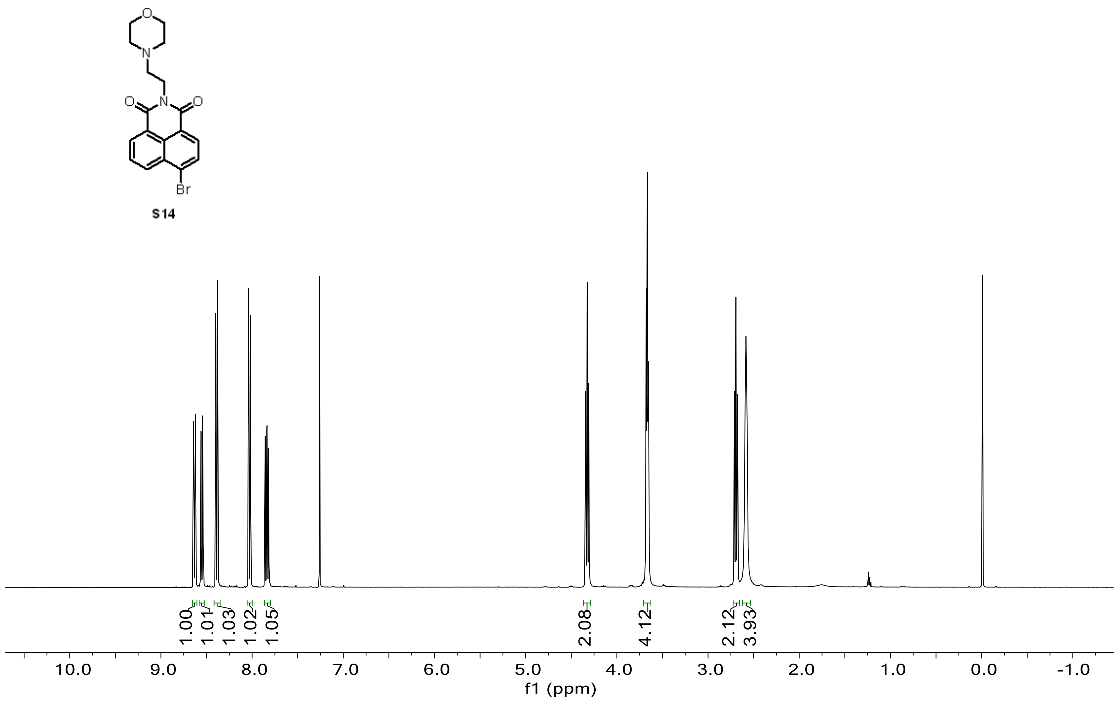

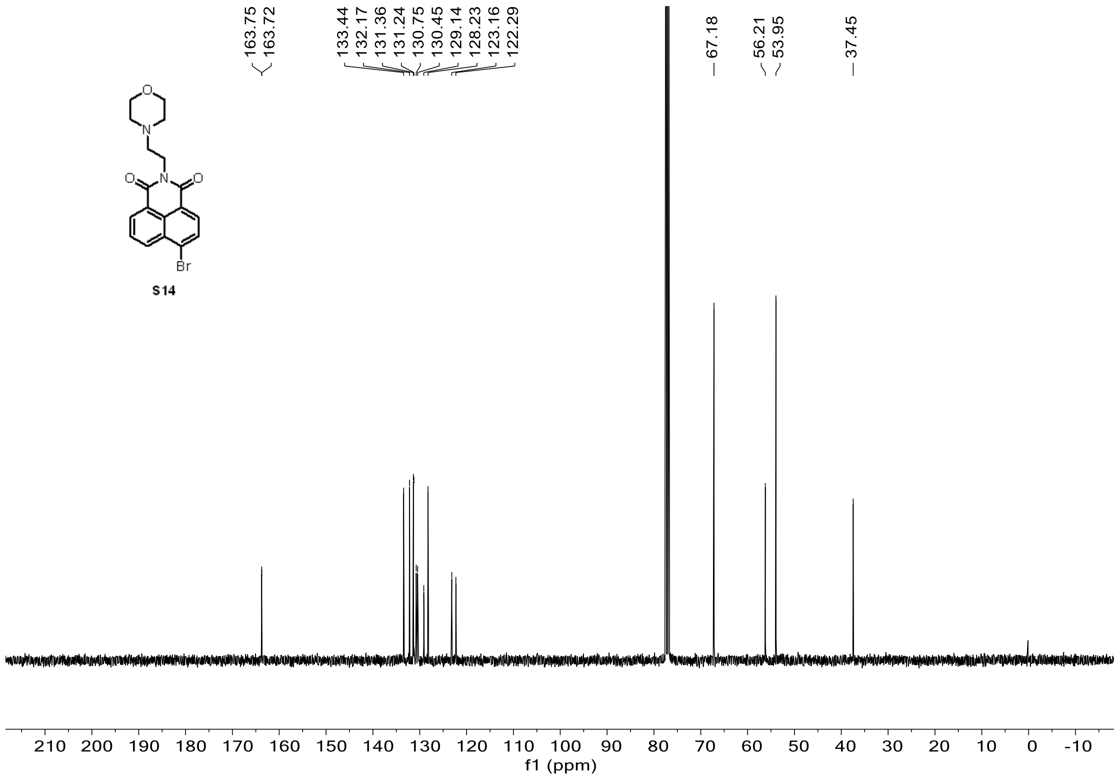

^{1}H NMR and ^{13}C NMR of F-Tz7

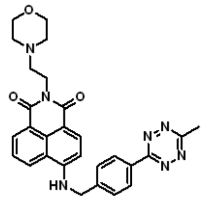

F-Tz7

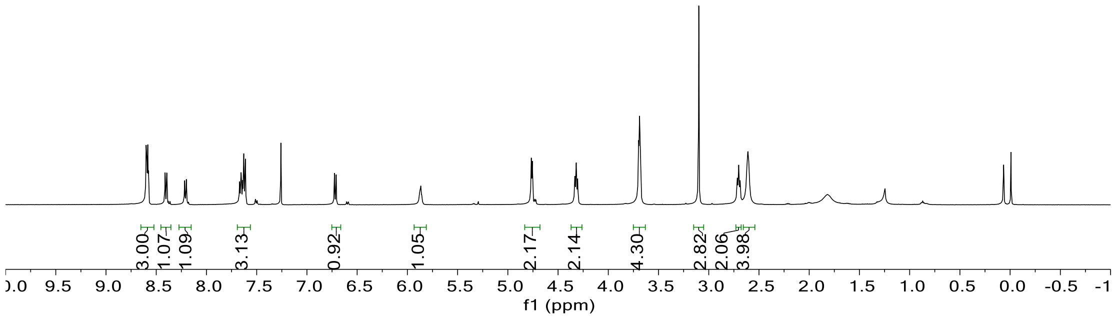

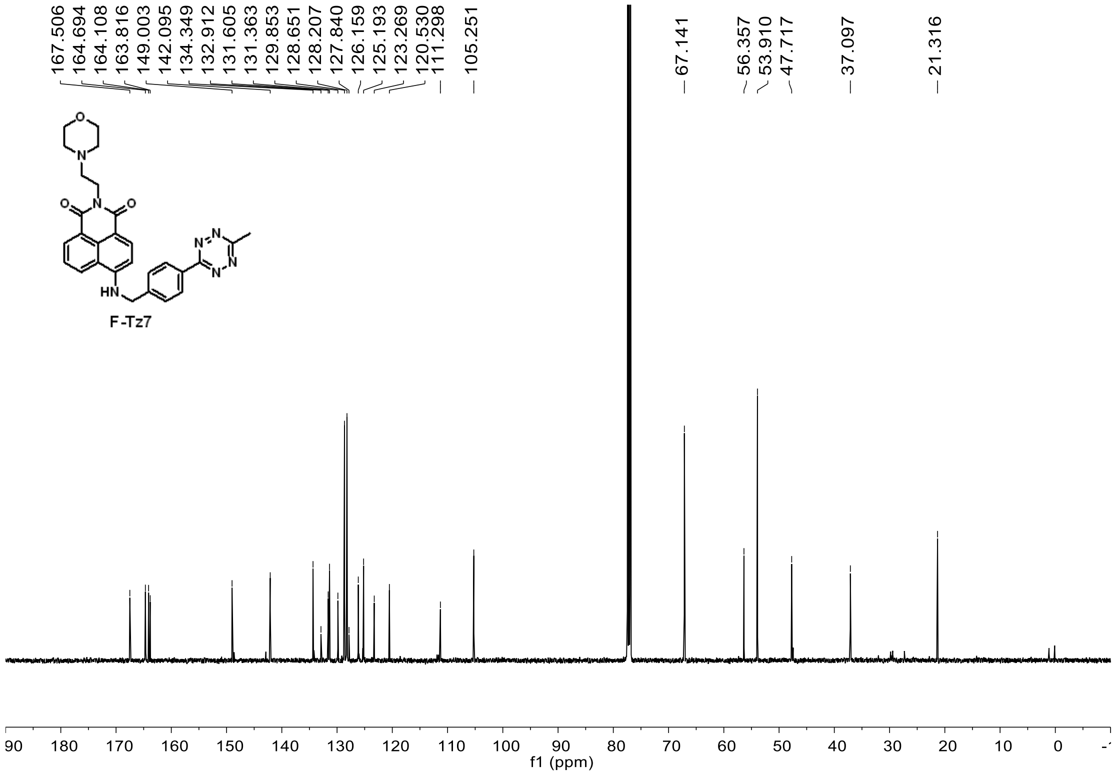

^{1}H NMR and ^{13}C NMR of S15

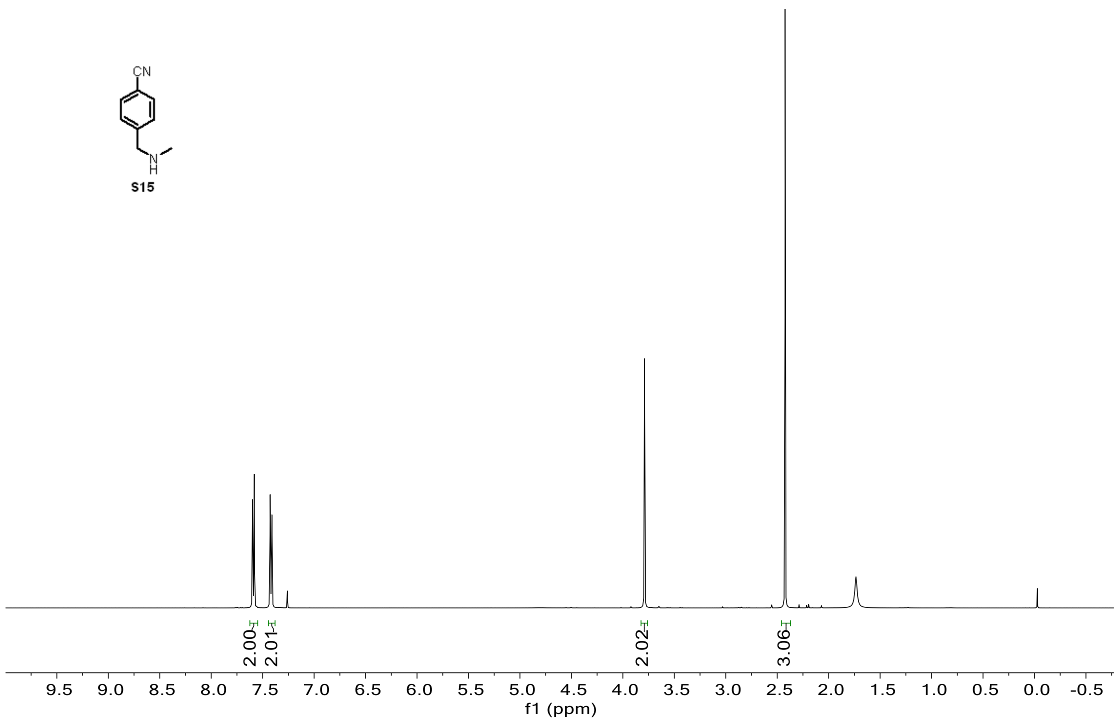

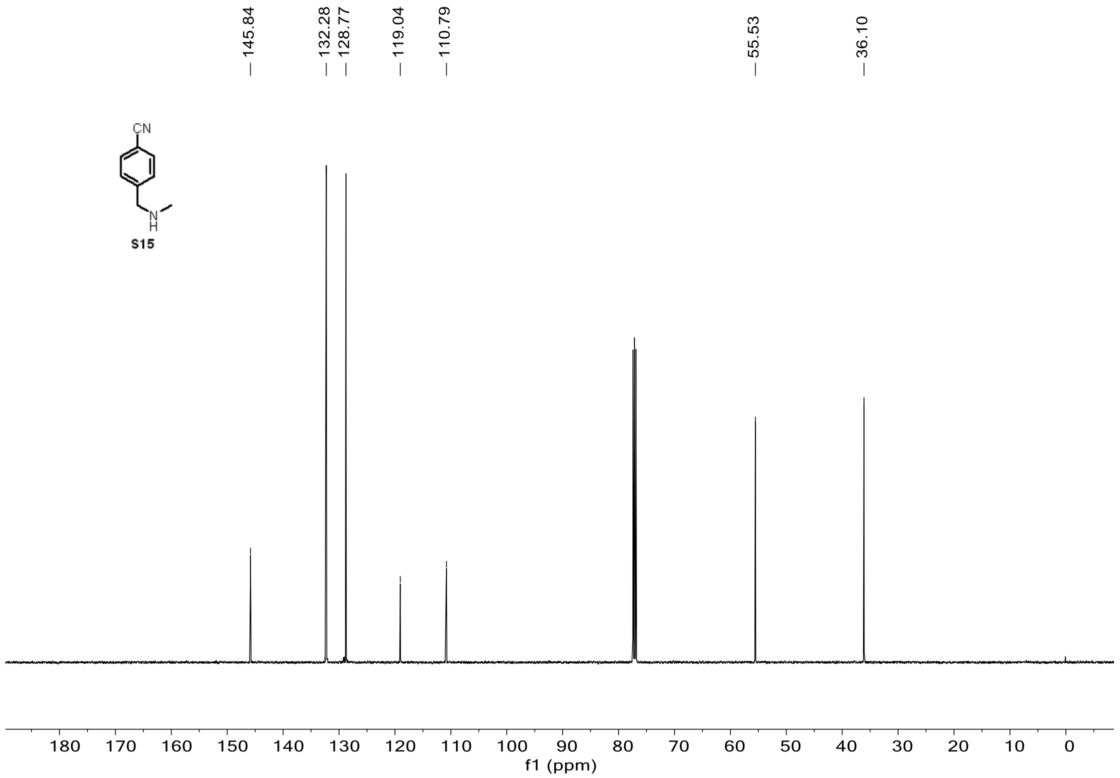

^{1}H NMR and ^{13}C NMR of S16

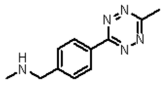

S16

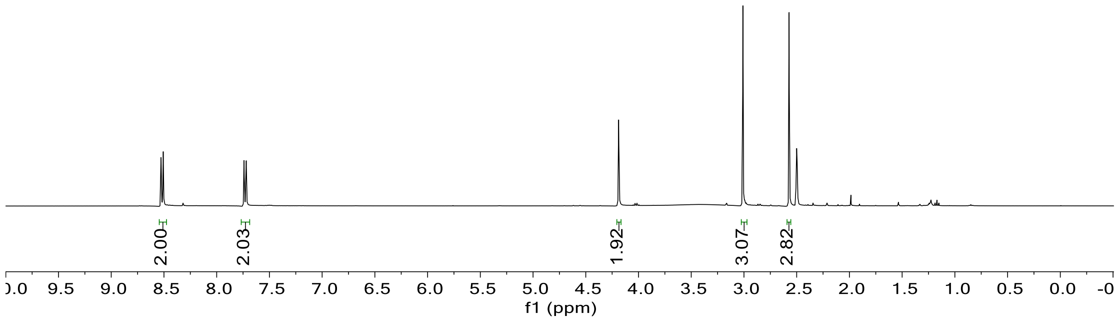

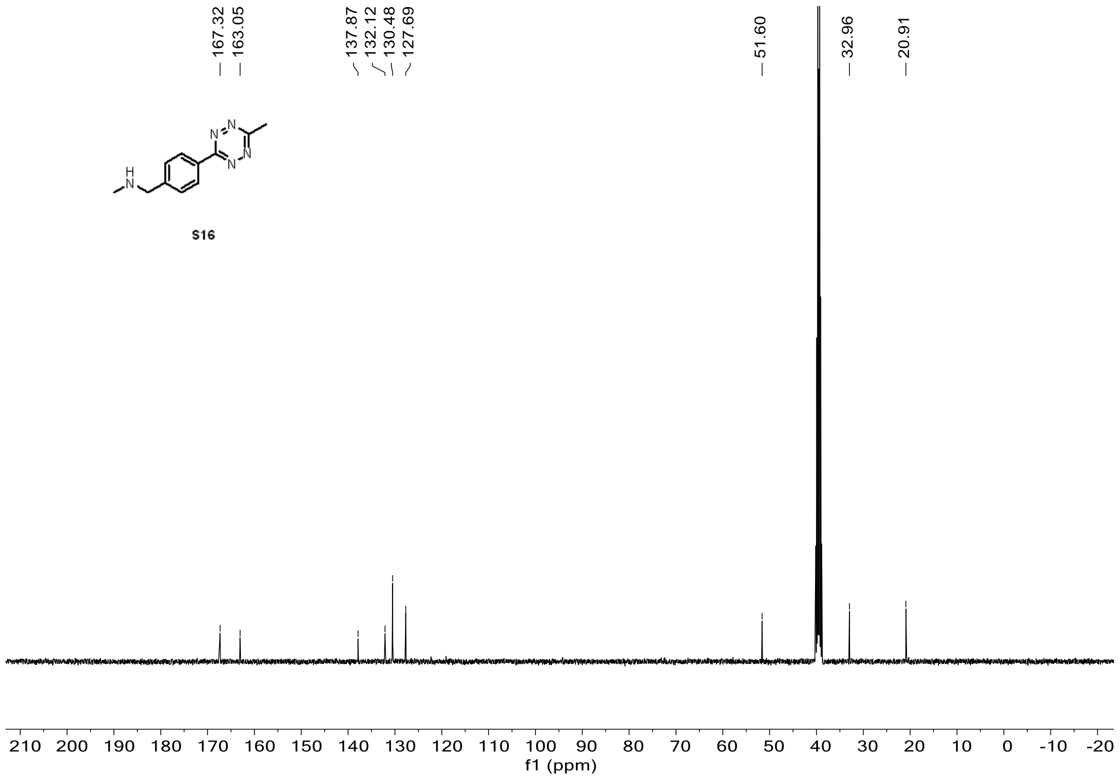

S16

^{1}H NMR and ^{13}C NMR of F-Tz8

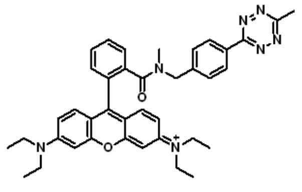

F-Tz8

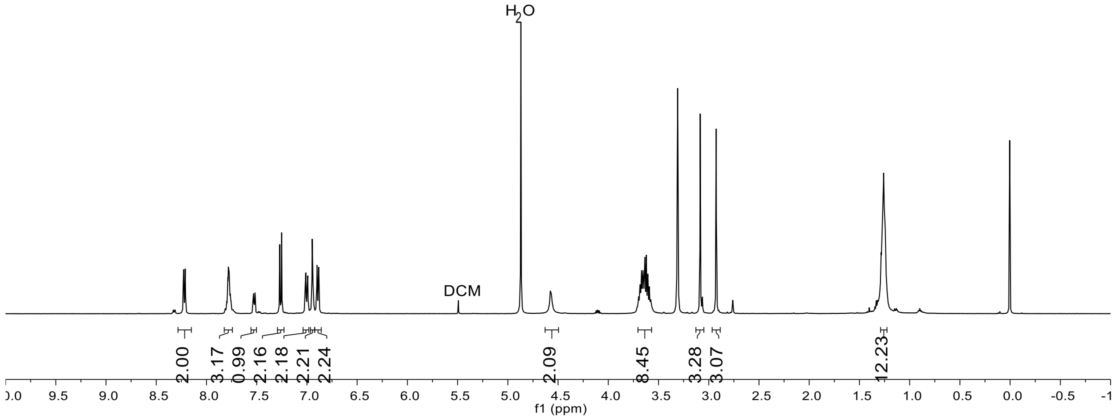

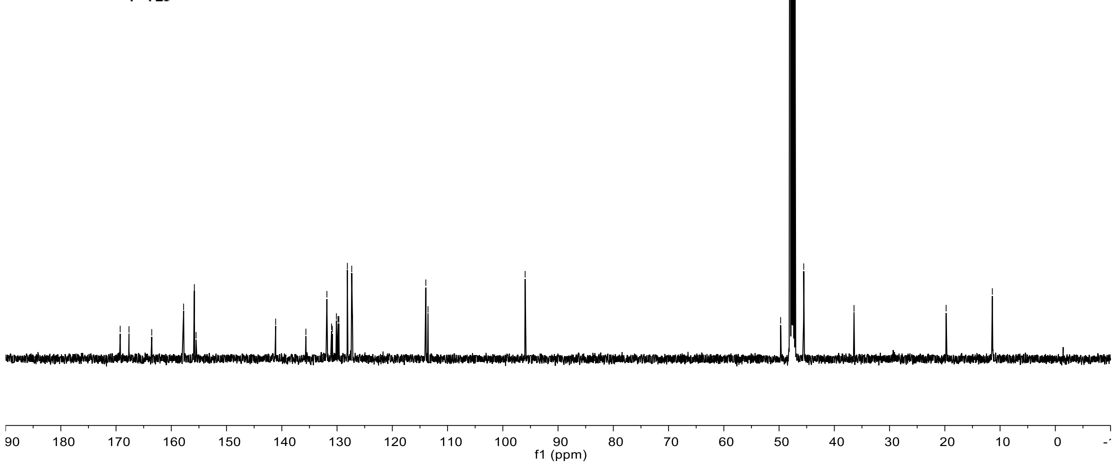

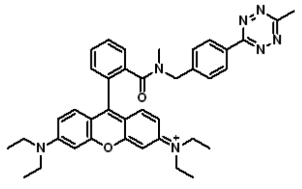

F-Tz8

^{1}H NMR of S18

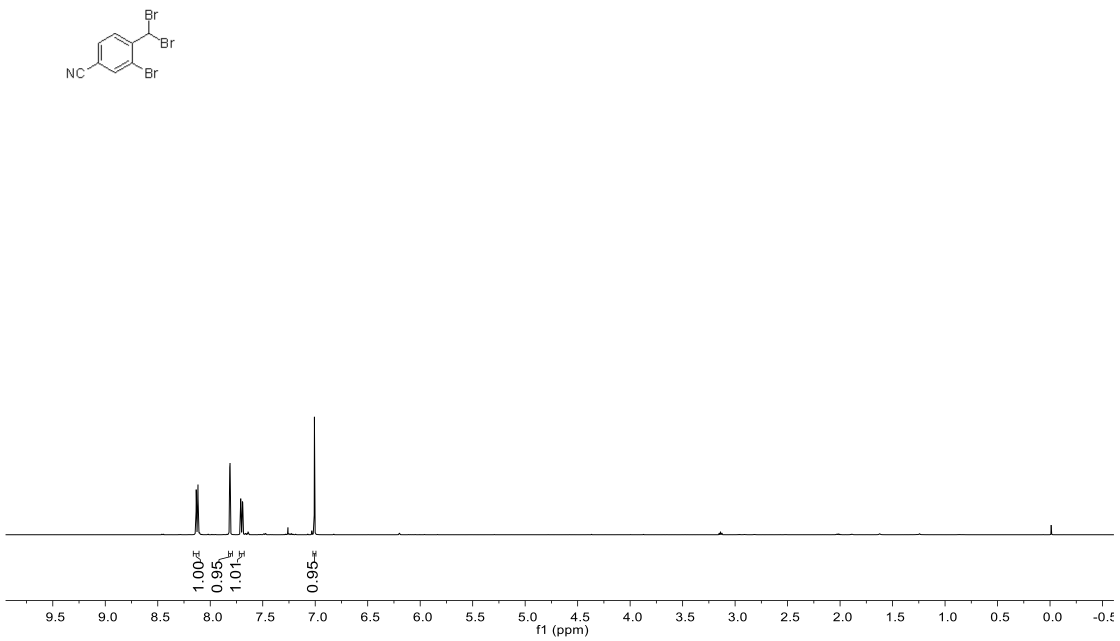

^{1}H NMR of S19

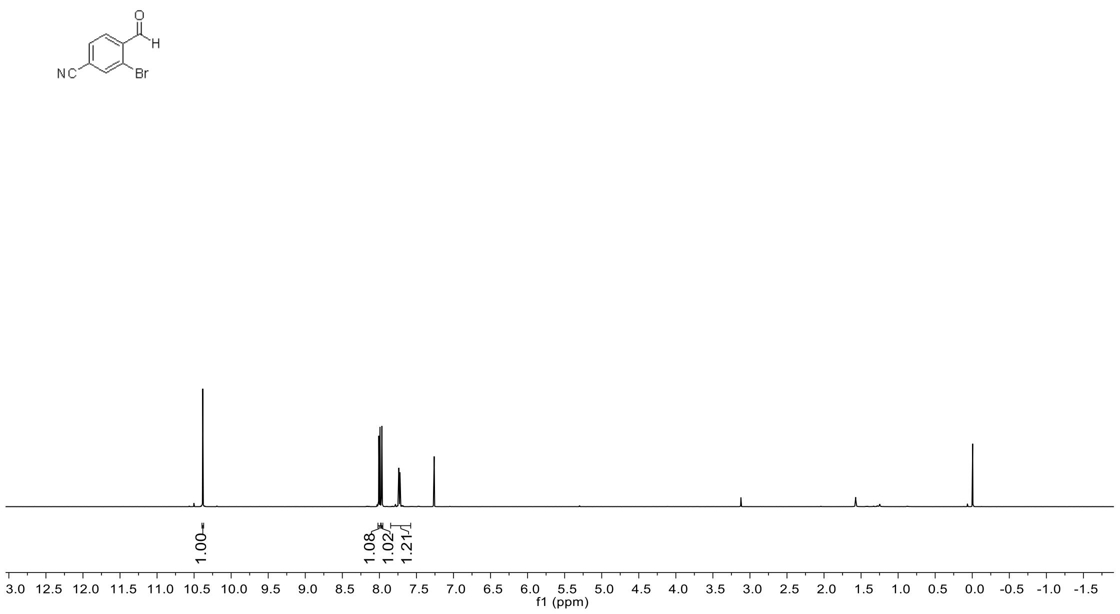

^{1}H NMR and ^{13}C NMR of S20

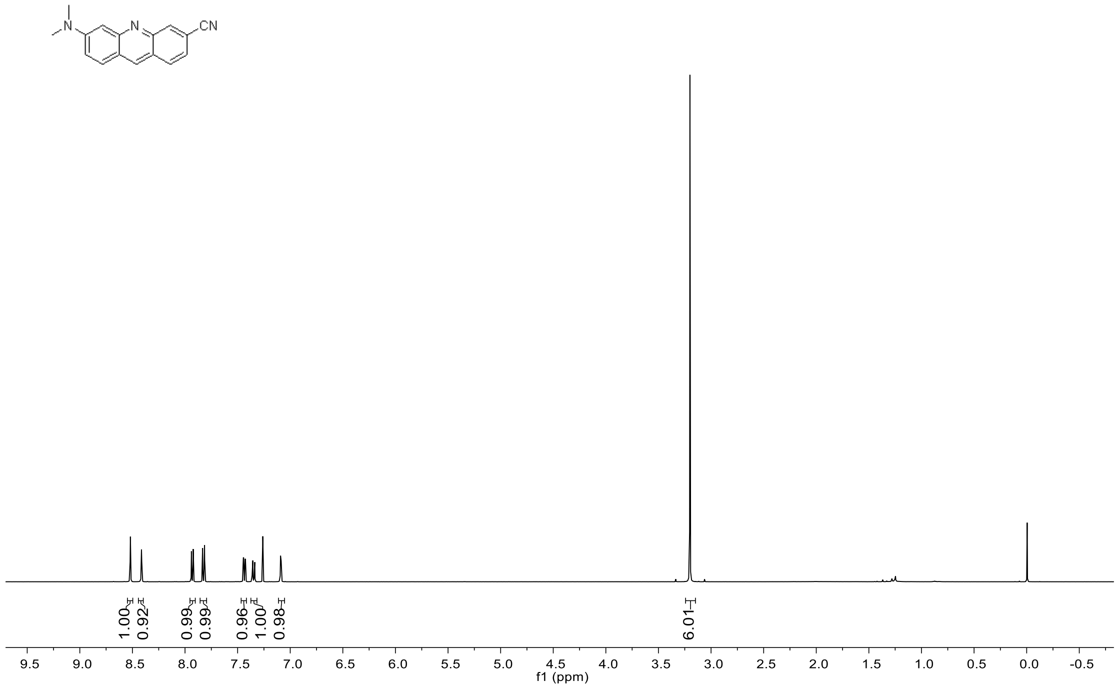

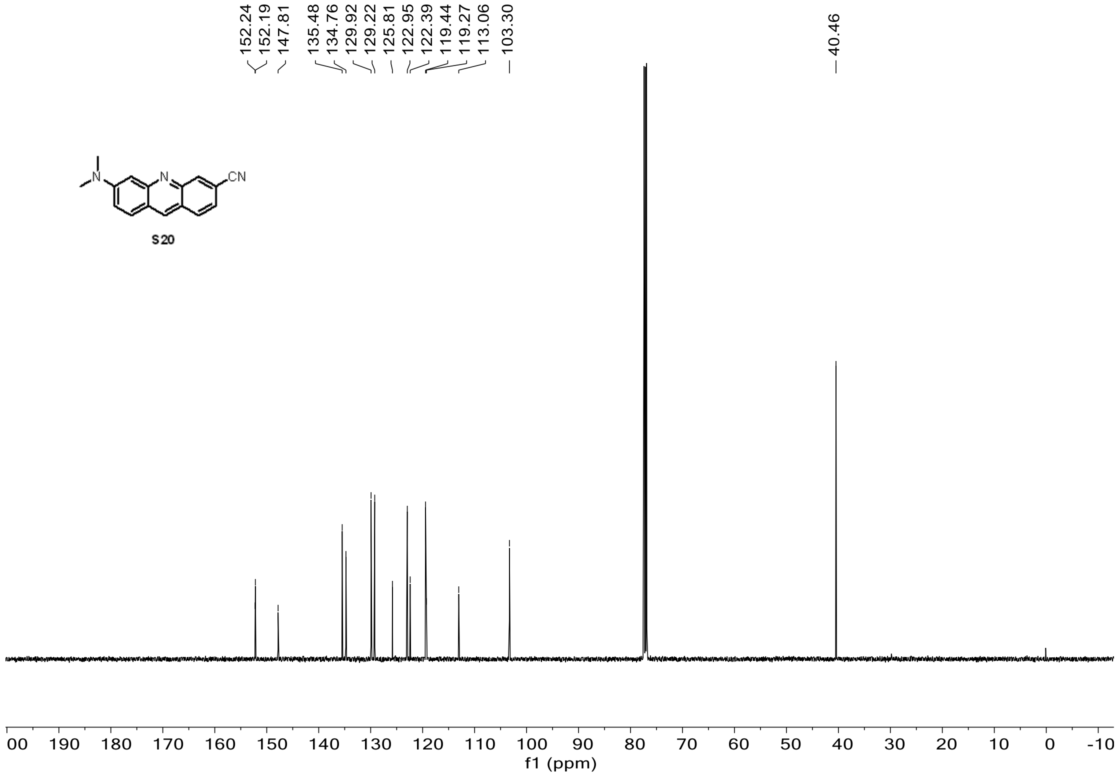

^{1}H NMR and ^{13}C NMR of F-Tz9

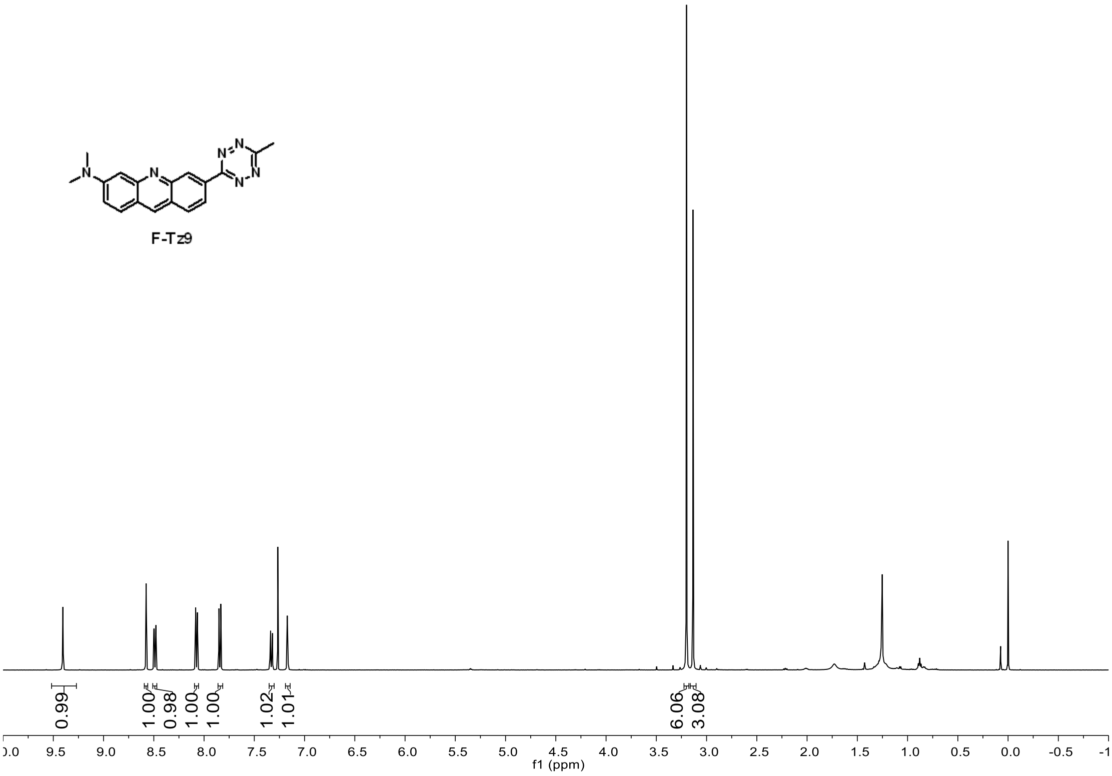

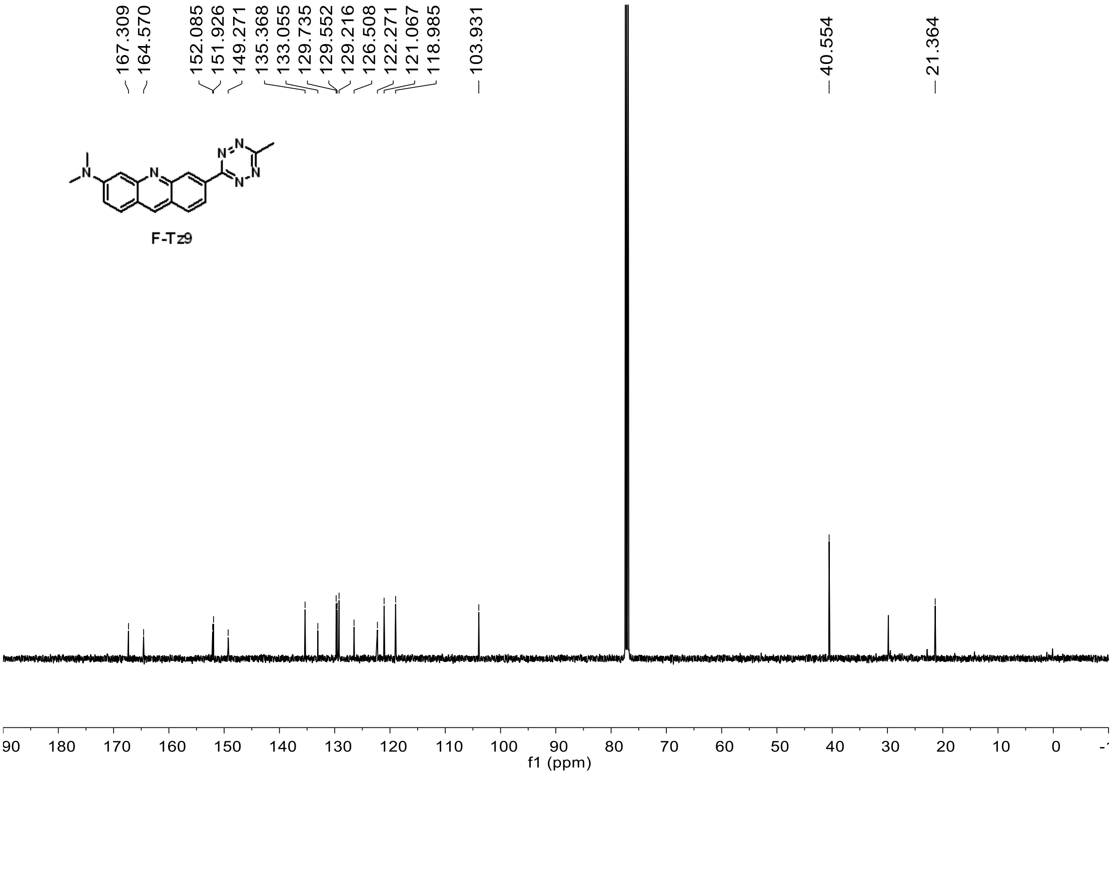

References

1 Selvaraj, R. & Fox, J. M. An efficient and mild oxidant for the synthesis of s-tetrazines. *Tetrahedron Letters* **55**, 4795-4797 (2014). https://doi.org:10.1016/j.tetlet.2014.07.012

2 Stabile, P. *et al.* Mild and convenient one-pot synthesis of 1,3,4-oxadiazoles. *Tetrahedron Letters* **51**, 4801-4805 (2010). https://doi.org:10.1016/j.tetlet.2010.06.139

3 Wang, Y.-J., Zhang, G.-Y., Shoberu, A. & Zou, J.-P. Iron-catalyzed oxidative amidation of acylhydrazines with amines. *Tetrahedron Letters* **80** (2021). https://doi.org:10.1016/j.tetlet.2021.153316

4 Wang, Q. *et al.* Design, synthesis and biological evaluation of acyl hydrazones-based derivatives as RXRalpha-targeted anti-mitotic agents. *Bioorg Chem* **128**, 106069 (2022). https://doi.org:10.1016/j.bioorg.2022.106069

5 Yang, J., Karver, M. R., Li, W., Sahu, S. & Devaraj, N. K. Metal-catalyzed one-pot synthesis of tetrazines directly from aliphatic nitriles and hydrazine. *Angew Chem Int Ed Engl* **51**, 5222-5225 (2012). https://doi.org:10.1002/anie.201201117

6 Lambert, W. D. *et al.* Installation of Minimal Tetrazines through Silver-Mediated Liebeskind-Srogl Coupling with Arylboronic Acids. *J Am Chem Soc* **141**, 17068-17074 (2019). https://doi.org:10.1021/jacs.9b08677

7 Xu, Z. *et al.* Design, synthesis, and evaluation of novel porcupine inhibitors featuring a fused 3-ring system based on the 'reversed' amide scaffold. *Bioorg Med Chem* **24**, 5861-5872 (2016). https://doi.org:10.1016/j.bmc.2016.09.041

8 Jiang, C. *et al.* Direct Transformation of Nitrogen-Containing Methylheteroarenes to Heteroaryl Nitrile by Sodium Nitrite. *Org Lett* **24**, 6341-6345 (2022). https://doi.org:10.1021/acs.orglett.2c02596

9 Xie, X. *et al.* Topology-Selective, Fluorescent "Light-Up" Probes for G-Quadruplex DNA Based on Photoinduced Electron Transfer. *Chemistry* **24**, 12638-12651 (2018). https://doi.org:10.1002/chem.201801701

10 Xing, W. *et al.* A PET-based fluorescent probe for monitoring labile Fe(II) pools in macrophage activations and ferroptosis. *Chem Commun (Camb)* **58**, 2979-2982 (2022). https://doi.org:10.1039/d1cc06611k

11 Shi, B. *et al.* Turn on fluorescent detection of hydrazine with a 1,8-naphthalimde derivative. *Dyes and Pigments* **147**, 152-159 (2017). https://doi.org:10.1016/j.dyepig.2017.08.010

12 Larkin, J. D., Frimat, K. A., Fyles, T. M., Flower, S. E. & James, T. D. Boronic acid based photoinduced electron transfer (PET) fluorescence sensors for saccharides. *New Journal of Chemistry* **34** (2010). https://doi.org:10.1039/c0nj00578a

13 Loehr, M. O. & Luedtke, N. W. A Kinetic and Fluorogenic Enhancement Strategy for Labeling of Nucleic Acids. *Angew Chem Int Ed Engl* **61**, e202112931 (2022). https://doi.org:10.1002/anie.202112931

14 Schiffler, M. A. *et al.* Discovery and Characterization of 2-Acylaminoimidazole Microsomal Prostaglandin E Synthase-1 Inhibitors. *J Med Chem* **59**, 194-205 (2016). https://doi.org:10.1021/acs.jmedchem.5b01249

15 Dubost, E., Fossey, C., Cailly, T., Rault, S. & Fabis, F. Selective ortho-bromination of substituted benzaldoximes using Pd-catalyzed C-H activation: application to the synthesis of substituted 2-bromobenzaldehydes. *J Org Chem* **76**, 6414-6420 (2011). https://doi.org:10.1021/jo200853j

16 Fee, J. A. & Hildenbrand, P. G. On the development of a well-defined source of superoxide ion for studies with biological systems. *FEBS Letters* **39**, 79-82 (1974). https://doi.org:10.1016/0014-5793(74)80021-9
17 Ozawa, T. & Hanaki, A. On a spectrally well-defined and stable source of superoxide ion, O−2. *FEBS Letters* **74**, 99-102 (1977). https://doi.org:10.1016/0014-5793(77)80762-x
18 Sawyer, D. T. & Valentine, J. S. How super is superoxide? *Accounts of Chemical Research* **14**, 393-400 (2002). https://doi.org:10.1021/ar00072a005
