# Supplementary material for: 1,2,4,5-Tetrazine-tethered probes for fluorogenically imaging superoxide in live cells with ultrahigh specificity
Source: Nat Commun. 2023 Mar 14;14:1401. doi: 10.1038/s41467-023-37121-8 (PMC10014963; doi:10.1038/s41467-023-37121-8)
Supplement: Supplementary file 3 — Description of Additional Supplementary Files [file 41467_2023_37121_MOESM3_ESM.pdf]

Description of Additional Supplementary Files

Supplementary Data 1: High-content screening results.

Supplementary Data 2: Summarization on various detection methods for superoxide.
