# Supplementary material for: 1,2,4,5-Tetrazine-tethered probes for fluorogenically imaging superoxide in live cells with ultrahigh specificity
Source: Nat Commun. 2023 Mar 14;14:1401. doi: 10.1038/s41467-023-37121-8 (PMC10014963; doi:10.1038/s41467-023-37121-8)
Supplement: Supplementary file 6 — Reporting Summary [file 41467_2023_37121_MOESM6_ESM.pdf]

nature portfolio

Corresponding author(s): Yi Wang and Xin Li

Last updated by author(s): Feb 21, 2023

Reporting Summary

Nature Portfolio wishes to improve the reproducibility of the work that we publish. This form provides structure for consistency and transparency in reporting. For further information on Nature Portfolio policies, see our Editorial Policies and the Editorial Policy Checklist.

Statistics

For all statistical analyses, confirm that the following items are present in the figure legend, table legend, main text, or Methods section.

n/a	Confirmed	
☐	☒	The exact sample size (n) for each experimental group/condition, given as a discrete number and unit of measurement
☐	☒	A statement on whether measurements were taken from distinct samples or whether the same sample was measured repeatedly
☐	☒	The statistical test(s) used AND whether they are one- or two-sided<br>*Only common tests should be described solely by name; describe more complex techniques in the Methods section.*
☒	☐	A description of all covariates tested
☒	☐	A description of any assumptions or corrections, such as tests of normality and adjustment for multiple comparisons
☐	☒	A full description of the statistical parameters including central tendency (e.g. means) or other basic estimates (e.g. regression coefficient) AND variation (e.g. standard deviation) or associated estimates of uncertainty (e.g. confidence intervals)
☐	☒	For null hypothesis testing, the test statistic (e.g. F, t, r) with confidence intervals, effect sizes, degrees of freedom and P value noted<br>*Give P values as exact values whenever suitable.*
☒	☐	For Bayesian analysis, information on the choice of priors and Markov chain Monte Carlo settings
☒	☐	For hierarchical and complex designs, identification of the appropriate level for tests and full reporting of outcomes
☐	☒	Estimates of effect sizes (e.g. Cohen's d, Pearson's r), indicating how they were calculated

Our web collection on statistics for biologists contains articles on many of the points above.

Software and code

Policy information about availability of computer code

Data collection	Hitachi U-3010 spectrophotometer (UV Solutions 2.2) and Agilent Cary Eclipse Fluorescence Spectrophotometer (Scan.lnk) were used to characterize the photophysical properties of the probes. Bruker 500 Fourier transform spectrometer, SHIMADZU LCMS-2020 spectrometer (LabSolutions.lnk) and Agilent 6224 TOF LC/MS spectrometer were used to characterize the structure of the compounds. Leica TCS SP8 (LAS X) was used for cell imaging experiments. The ImageXpress Micro® Confocal High-Content Imaging System (Molecular Devices, LLC, San Jose, CA, USA) was used to acquire images in High-content screening.
Data analysis	In the fluorescence confocal imaging experiments, the fluorescence intensity was quantified via the software “ImageJ” (ImageJ-win64.exe) and the data were analyzed by Graphpad prism 8.0.2 software .<br>The fluorescence spectra and absorption spectra were processed via the software “Origin 2021”.<br>DFT and TD-DFT calculations were carried out using Gaussian 16A.<br>The analysis module of the MetaXpress® High-Content Image and Analysis Software (Molecular Devices) was used to analyze the images and the data were analyzed by Graphpad prism 8.0 software .

For manuscripts utilizing custom algorithms or software that are central to the research but not yet described in published literature, software must be made available to editors and reviewers. We strongly encourage code deposition in a community repository (e.g. GitHub). See the Nature Portfolio guidelines for submitting code & software for further information.

Data

Policy information about availability of data

All manuscripts must include a data availability statement. This statement should provide the following information, where applicable:

- Accession codes, unique identifiers, or web links for publicly available datasets
- A description of any restrictions on data availability
- For clinical datasets or third party data, please ensure that the statement adheres to our policy

The authors declare that the data supporting the findings from this study are available in the article and its supplementary information. Source data are provided with this paper.

Human research participants

Policy information about studies involving human research participants and Sex and Gender in Research.

Reporting on sex and gender	N/A
Population characteristics	N/A
Recruitment	N/A
Ethics oversight	N/A

Note that full information on the approval of the study protocol must also be provided in the manuscript.

Field-specific reporting

Please select the one below that is the best fit for your research. If you are not sure, read the appropriate sections before making your selection.

☒ Life sciences ☐ Behavioural & social sciences ☐ Ecological, evolutionary & environmental sciences

For a reference copy of the document with all sections, see nature.com/documents/nr-reporting-summary-flat.pdf

Life sciences study design

All studies must disclose on these points even when the disclosure is negative.

Sample size	For cell experiments, three biologically independent replications were used for each experimental group. The sample sizes were not predetermined but we performed TTC staining experiments with the final group sizes based on existing published literature of similar experiments (Xiao H, et al. Circ Res. 2022, 130, e3-e17; Gao C et al. Cardiovasc Res. 2020, 116, 647-657). For the hemodynamic experiments, n≥6 was chosen based on the previous publications in this filed (Xiao CY et.al. Circulation. 2001, 104, 2210-2215; and Zhang J, et.al. Aging (Albany NY). 2020, 12, 24270-24287).<br>In animal experiments of protective effect of coprostanone against mice myocardial I/R injury, a total of 132 mice were used, which were divided into sham operation group, I/R group, 5αCh3 low dose group (50 mg/kg), 5αCh3 high dose group (100 mg/kg) by the random number table method. Among the survived mice, 16-18 in each group were used for TTC staining, 4 in each group was used for Western blotting. For hemodynamics, 41 mice were randomly divided into sham, sham + 5αCh3 (100 mg/kg), I/R, and I/R + 5αCh3 (100 mg/kg) groups. All the survived mice were used to record hemodynamic parameters.
Data exclusions	In the TTC staining test, one-three mice in each group were not analyzed because of the staining failure. The data of 15-16 mice were finally counted. No data were excluded for the other results. In the hemodynamics experiment, 1-3 mice in each group failed to record the hemedynamic parameters because of the failure of micro-cannulation.
Replication	All experiments were performed at least with three independent biological replicates.
Randomization	All mice and cells were randomly divided into different groups for the experiments.
Blinding	During the experiment, all mice and cells were numbered numerically and all the data were collected blinded to group allocation. For animal experiments, experimental operations were performed by designated individuals, while the image processing and data analysis were completed by other individuals.

Reporting for specific materials, systems and methods

We require information from authors about some types of materials, experimental systems and methods used in many studies. Here, indicate whether each material, system or method listed is relevant to your study. If you are not sure if a list item applies to your research, read the appropriate section before selecting a response.

Materials & experimental systems

n/a	Involved in the study
[ ]	[x] Antibodies
[ ]	[x] Eukaryotic cell lines
[x]	[ ] Palaeontology and archaeology
[ ]	[x] Animals and other organisms
[x]	[ ] Clinical data
[x]	[ ] Dual use research of concern

Methods

n/a	Involved in the study
[x]	[ ] ChIP-seq
[x]	[ ] Flow cytometry
[x]	[ ] MRI-based neuroimaging

Antibodies

Antibodies used

All the antibodies used in this manuscript are as follows:
HO-1/HMOX1 Rabbit Polyclonal antibody (Proteintech Group, Inc., Chicago, IL), Catalog number: 10701-1-AP, Lot number: 00105224;
SOD2 Polyclonal antibody (Proteintech Group, Inc., Chicago, IL), Catalog number: 24127-1-AP, Lot number: 00067140;
Beta Tubulin Polyclonal antibody (Proteintech Group, Inc., Chicago, IL), Catalog number: 10094-1-AP, Lot number: 0010735;
Nrf2 Rabbit Polyclonal antibody (Beyotime Biotechnology, Haimen, China), Catalog number: AF7623;
anti-cTnT rabbit polyclonal antibody, Servicebio, China, Catalog number: GB11364;
HRP-conjugated anti-Rabbit secondary antibody (Beyotime Biotechnology, Haimen, China), Catalog number: A0208;
HRP conjugated Goat anti-Rabbit IgG (H+L), Servicebio, China, Catalog number: GB23303;
Cy3 conjugated Goat anti-Rabbit IgG (H+L), Servicebio, China, Catalog number: GB21303.

Validation

HO-1/HMOX1 Rabbit Polyclonal antibody targets HO-1/HMOX1 in WB, IP, IHC, IF, FC, CoIP, ELISA applications, and showed reactivity with human, mouse, rat samples, which was validated in 604 published research papers.
SOD2 Polyclonal antibody targets SOD2 in IF, IHC, IP, WB, ELISA applications, and showed reactivity with human, mouse, rat samples, which was validated in 231 published research papers.
Beta Tubulin Polyclonal antibody targets Beta Tubulin in WB, IP, IHC, IF, FC, CoIP, ELISA applications, and showed reactivity with human, mouse, rat samples, which was validated in 563 published research papers.
Nrf2 Rabbit Polyclonal antibody targets Nrf2 in WB, IF, IHC applications, and showed reactivity with human, mouse, rat samples, which was validated by the manufacturer. All the representative pictures are displayed on the official website (https://www.beyotime.com/product/AF7623.htm).
Anti-cTnT rabbit polyclonal antibody targets cardic Troponin T in IF and IHC, and showed reactivity in mouse samples, which was validated with 1 published research article（Miao Y, Ding Z, Zou Z, et al. Am J Transl Res. 2020;12(9):5151-5169）.

Eukaryotic cell lines

Policy information about cell lines and Sex and Gender in Research

Cell line source(s)	A549, HepG2 and H9C2 cells were kindly provided by Stem Cell Bank, Chinese Academy of Sciences.
Authentication	The A549, HepG2 and H9C2 cells used were STR-approved by Stem Cell Bank, Chinese Academy of Sciences.
Mycoplasma contamination	All cells used were tested by Stem Cell Bank, Chinese Academy of Sciences for mycoplasma and all results were negative.
Commonly misidentified lines (See ICLAC register)	There is no commonly misidentified lines included in our study.

Animals and other research organisms

Policy information about studies involving animals; ARRIVE guidelines recommended for reporting animal research, and Sex and Gender in Research

Laboratory animals	Male adult C57BL/6 mice (8 weeks old) were obtained from SLAC ANIMAL Company, Shanghai, China. SD rats born at 0-3 d (P0-P3) regardless of gender was provided by Zhejiang Academy of Medical Sciences. The mice were housed in polypropylene cages at a fixed in-house temperature (24 ± 2 °C) and humidity (40 ± 5%), under a 12 h light/dark cycle and well ventilated, with free access to clean water and the commercial rodent chow (Hangzhou Sailuojin Biological Technology Co., Ltd, China).
Wild animals	The study did not involve wild animals.
Reporting on sex	We chose male mice as experimental animals, because basic studies have demonstrated that estrogen treatment prevents apoptosis and necrosis of cardiac and endothelial cells, which may cause unexplained impact on drug efficacy studies. SD rats born at 0-3 days (P0-P3) were used for cardiac myocytes preparation without consideration of gender.

Field-collected samples	There is no field-collected samples used in this study.
Ethics oversight	All the animal experiments were approved by Zhejiang University Laboratory Animal Center. The project approval number is ZJU20220096.

Note that full information on the approval of the study protocol must also be provided in the manuscript.
