# Supplementary material for: 1,2,4,5-Tetrazine-tethered probes for fluorogenically imaging superoxide in live cells with ultrahigh specificity
Source: Nat Commun. 2023 Mar 14;14:1401. doi: 10.1038/s41467-023-37121-8 (PMC10014963; doi:10.1038/s41467-023-37121-8)

1. All the blots of HO-1 and the corresponding β-tubulin

HO-1 (Sample order: 48 (sham), 39 (sham), 37 (I/R), 59 (I/R), 28 (5αCh3, 100 mg/kg), 57 (5αCh3, 100 mg/kg), 26 (5αCh3, 50 mg/kg), 54 (5αCh3, 50 mg/kg))


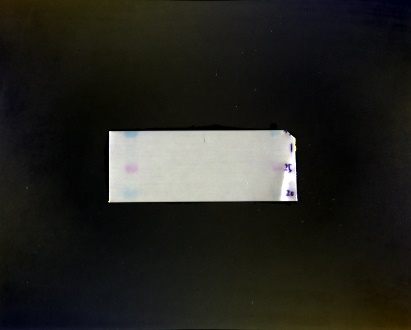

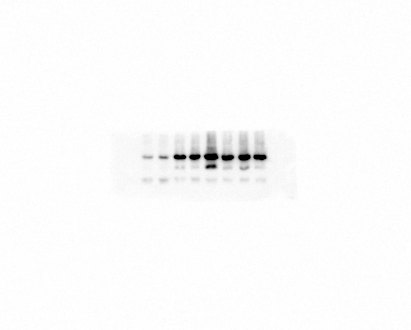

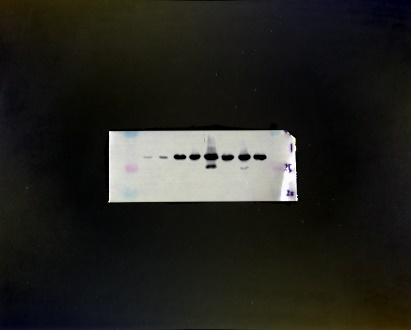


β-tubulin


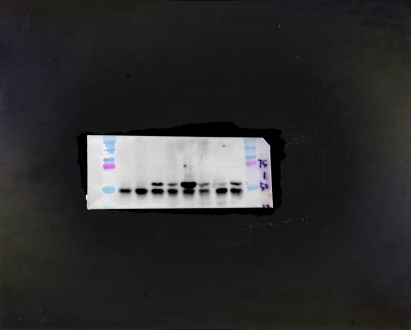

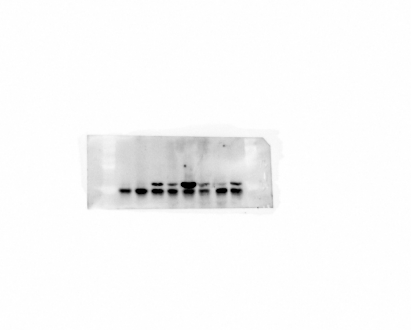

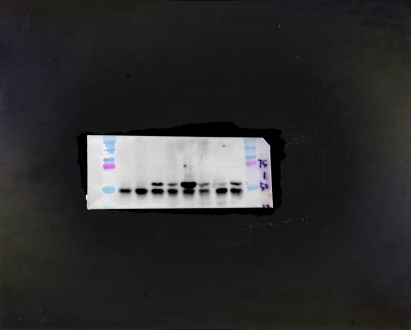


HO-1 (Sampling order: S1 (sham), S2 (sham), 12 (I/R), 30 (I/R), 69 (5αCh3, 100 mg/kg), 72 (5αCh3, 100 mg/kg), G52 (5αCh3, 50 mg/kg), H52 (5αCh3, 50 mg/kg))


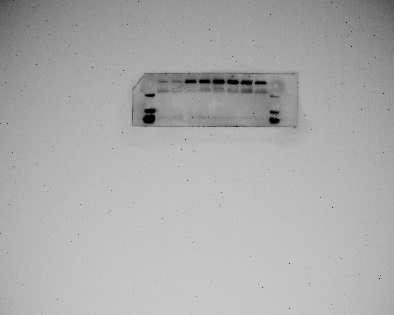

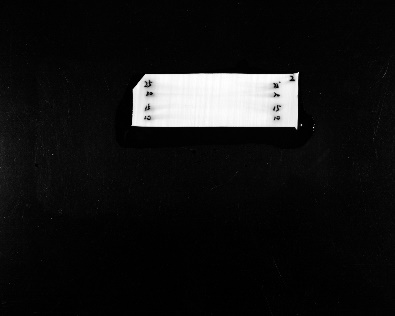

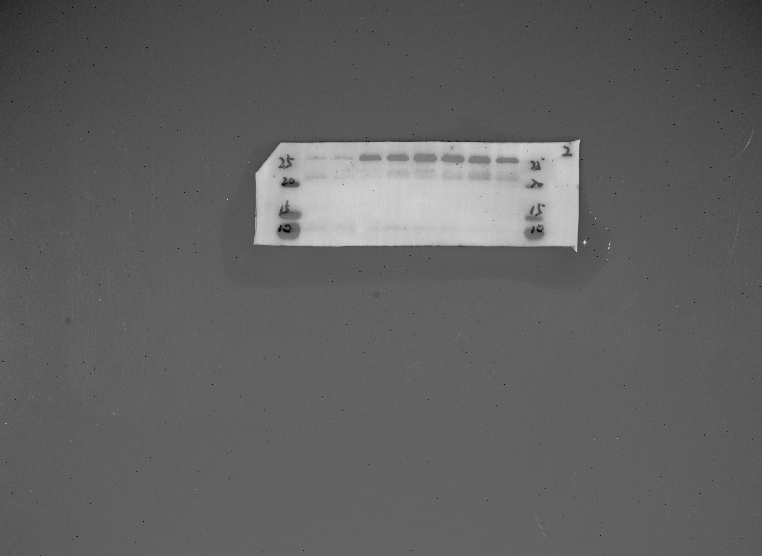


β-tubulin


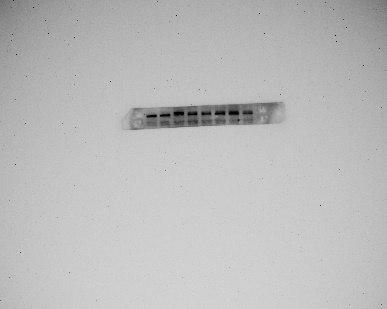

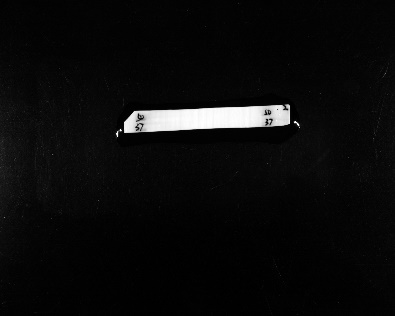

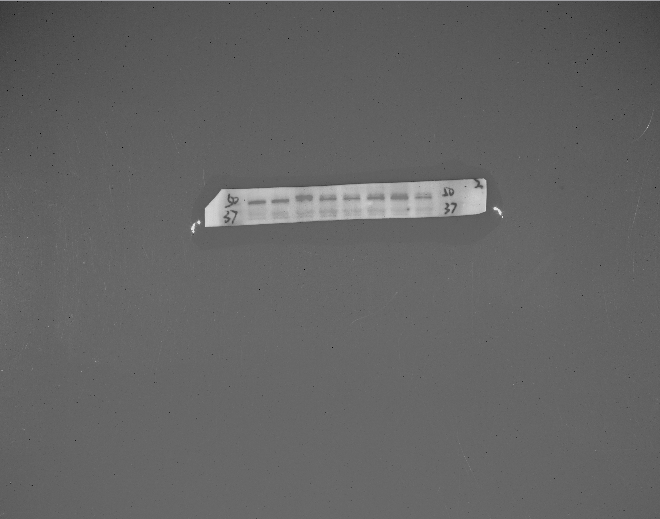


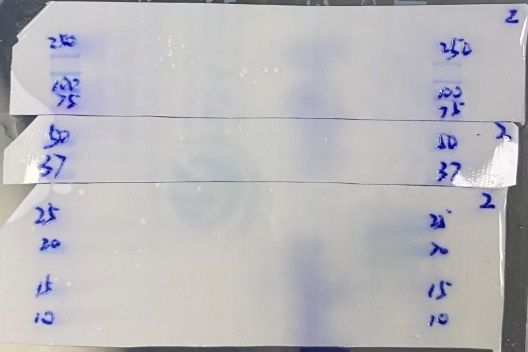


1. All the blots of NRF2 and the corresponding β-tubulin

NRF2 (S2 (sham), S3 (sham), 12 (I/R), 30 (I/R), 69 (5αCh3, 100 mg/kg), 72 (5αCh3, 100 mg/kg), G52 (5αCh3, 50 mg/kg), H52 (5αCh3, 50 mg/kg))

The Sample order of the four lanes after the second marker are S1 (sham), 31(I/R), 50 (5αCh3, 50 mg/kg), 67 (5αCh3, 100 mg/kg))


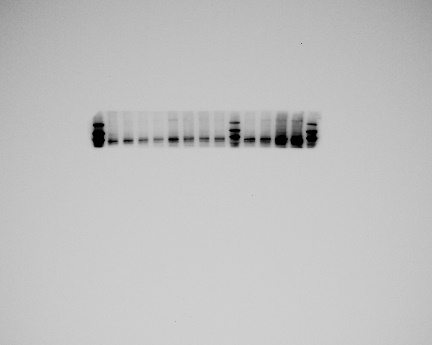


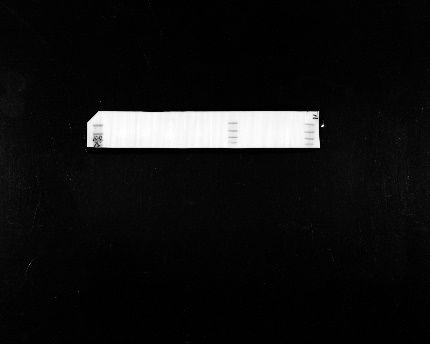

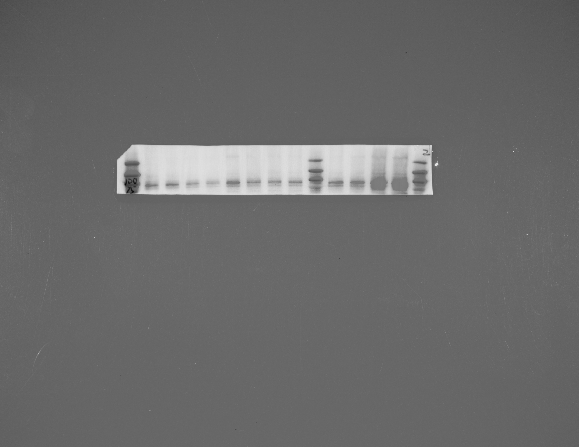


β-tubulin


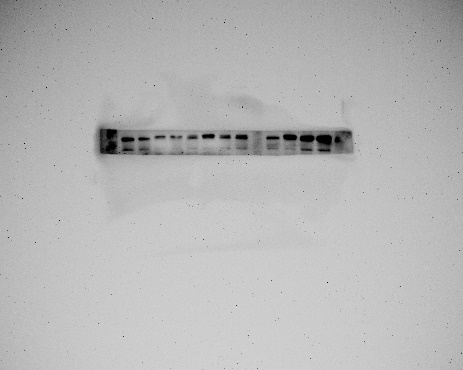


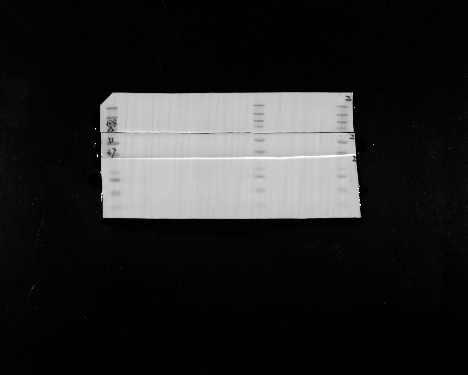

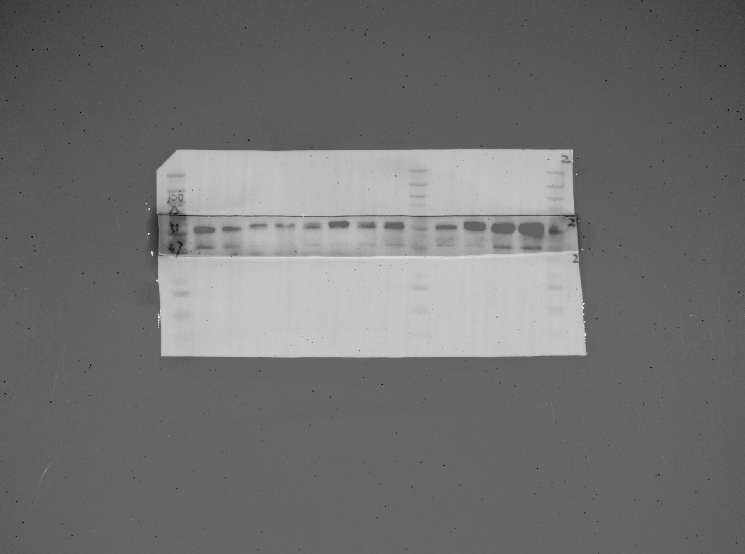


NRF2 (sample order: 46 (sham), 59 (I/R), 55 (5αCh3, 50 mg/kg), 60 (5αCh3, 100 mg/kg))


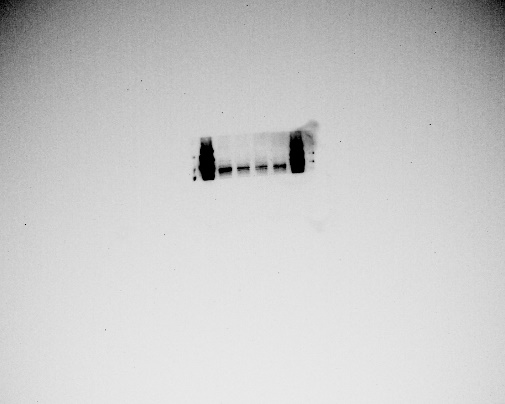

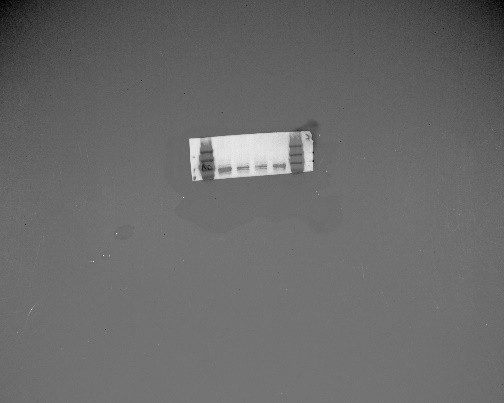

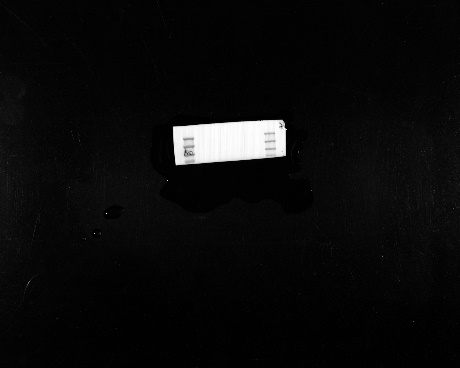


β-tubulin


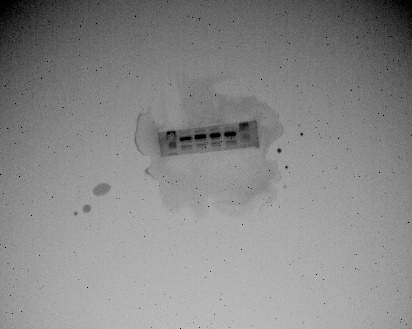

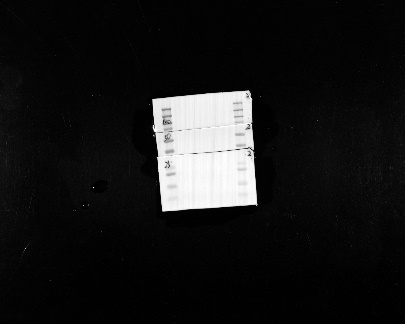

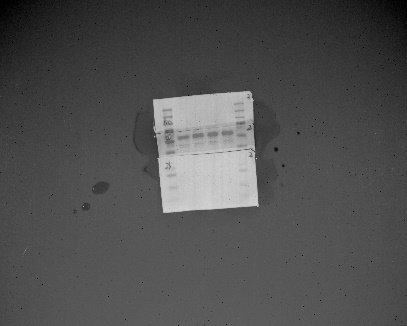


1. All the blots of SOD2 and the corresponding β-tubulin

SOD2 (Sample order: 46 (sham), 23 (sham), 31 (I/R), 49 (I/R), 57 (5αCh3, 100 mg/kg), 60 (5αCh3, 100 mg/kg), 54 (5αCh3, 50 mg/kg), 58 (5αCh3, 50 mg/kg))


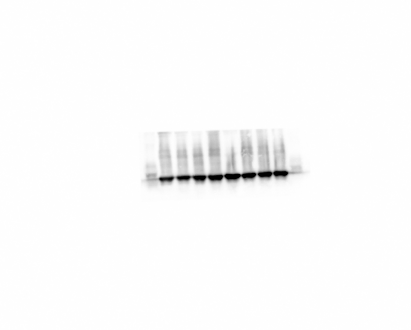

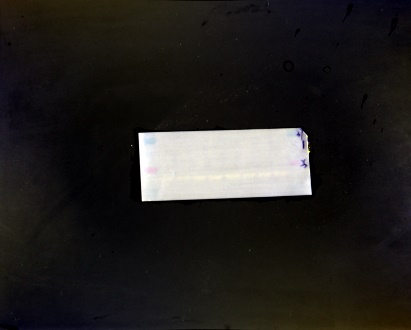

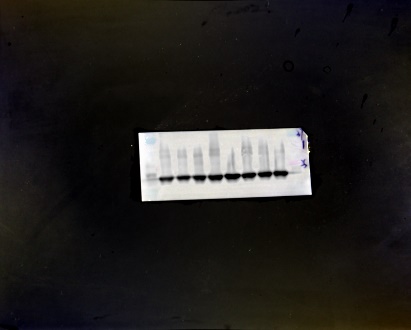


β-tubulin


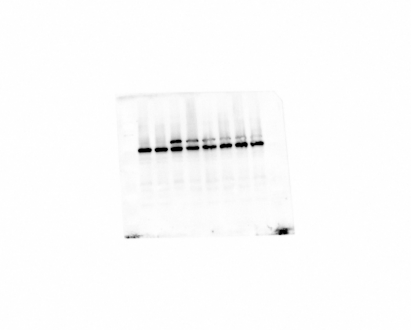

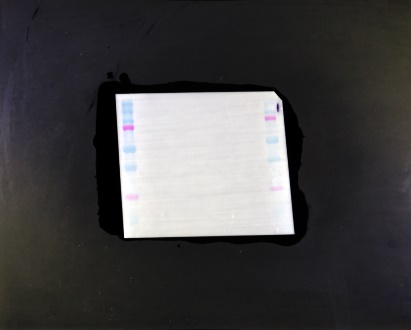

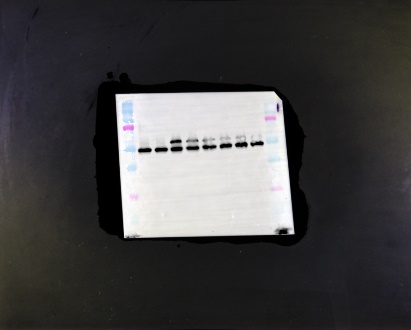


SOD2 (Sample order S3 (sham), 31 (I/R), G52 (5αCh3, 50 mg/kg), 72 (5αCh3, 100 mg/kg))


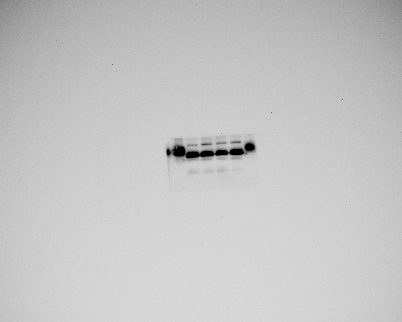

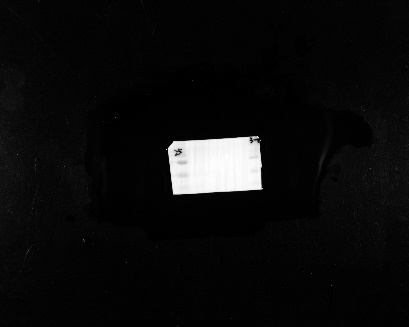

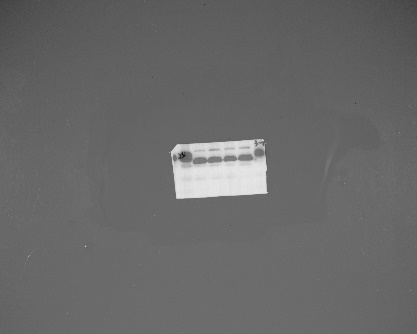


β-tubulin


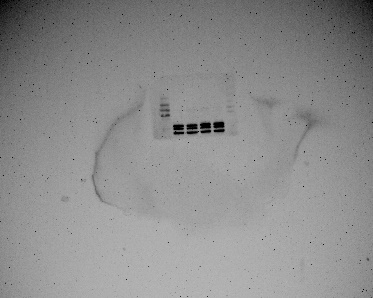

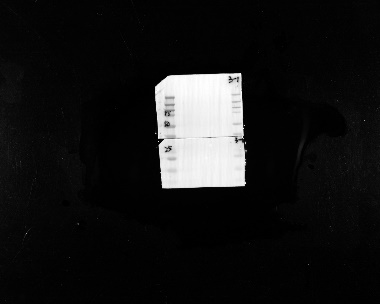

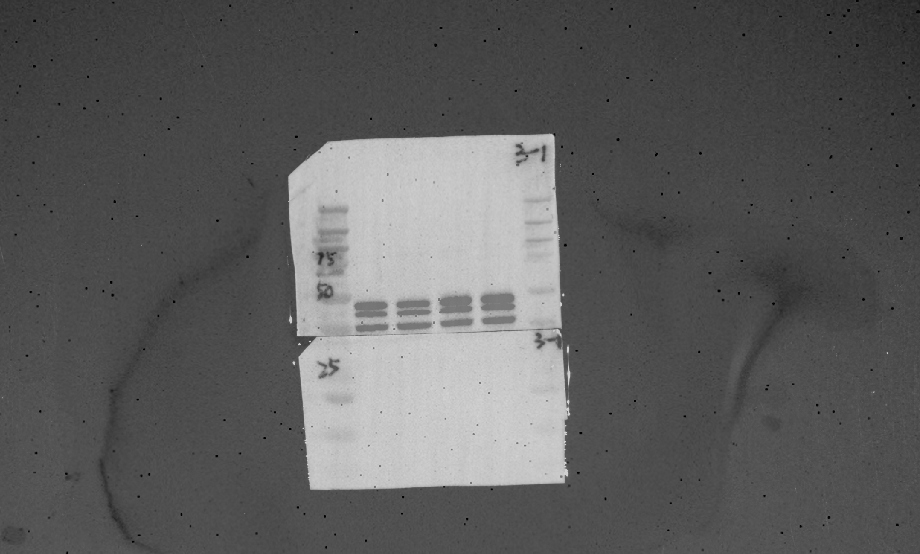


SOD2 (Sample order S2 (sham), 30 (I/R), H52 (5αCh3, 50 mg/kg), 69 (5αCh3, 100 mg/kg))


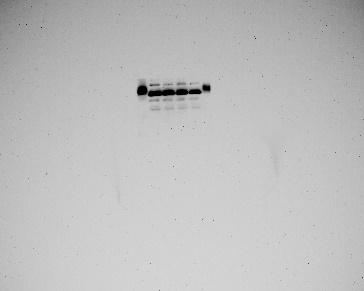

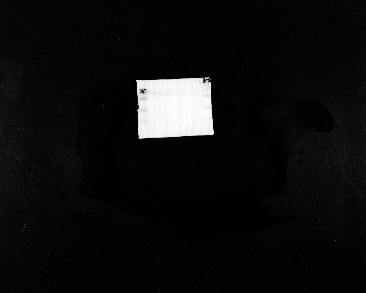

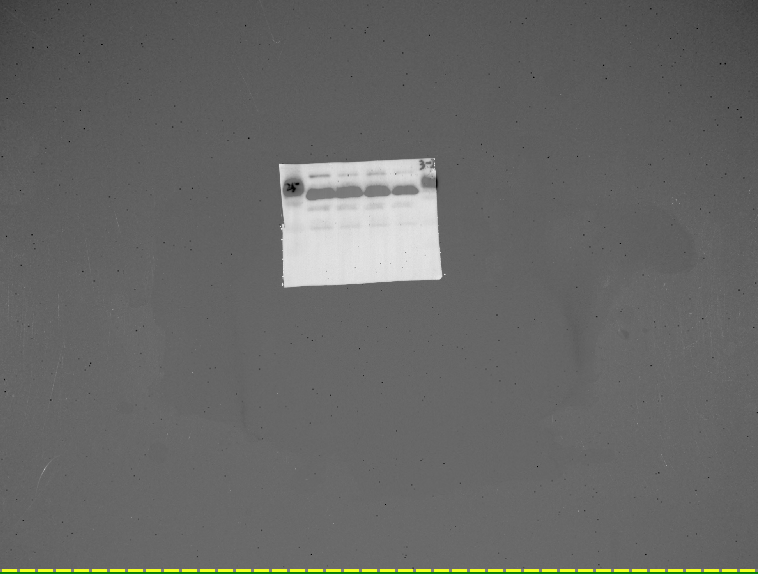


β-tubulin


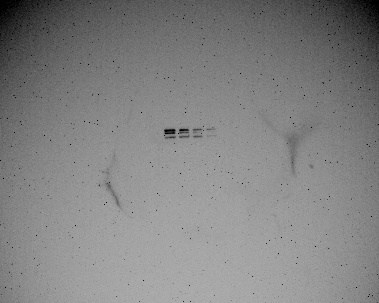

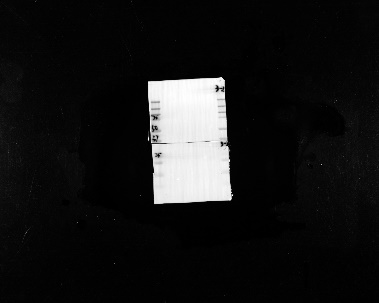

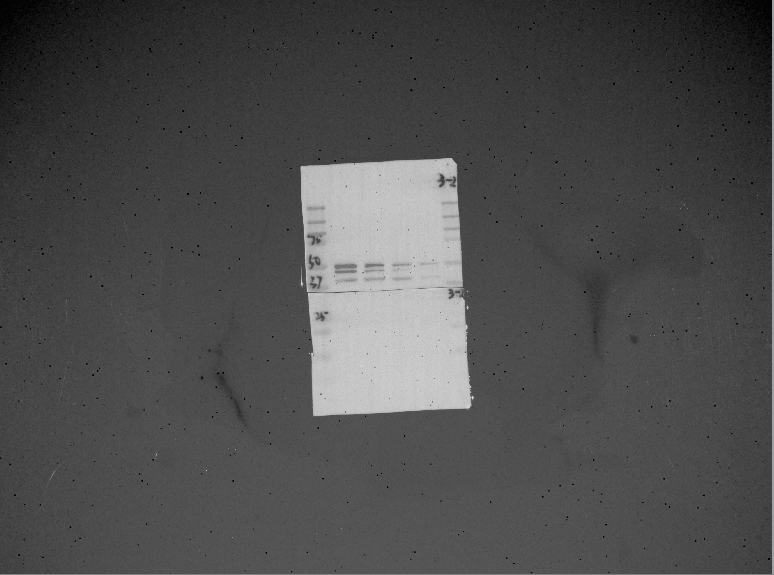

Supplement: Supplementary file 7 — Source Data [file 41467_2023_37121_MOESM7_ESM.zip › Source Data/Source blots.docx]
